# ClustAGE: a tool for clustering and distribution analysis of bacterial accessory genomic elements

**DOI:** 10.1186/s12859-018-2154-x

**Published:** 2018-04-20

**Authors:** Egon A. Ozer

**Affiliations:** Department of Medicine, Division of Infectious Diseases, Northwestern University Feinberg School of Medicine, Chicago, Illinois USA

**Keywords:** Bacteria, Comparative genomics, Accessory genome, Flexible genome

## Abstract

**Background:**

The non-conserved accessory genome of bacteria can be associated with important adaptive characteristics that can contribute to niche specificity or pathogenicity of strains. High degrees of structural and compositional diversity in genomic islands and other elements of the accessory genome can complicate characterization of accessory genome contents among populations of strains. Methods for easily and effectively defining the distributions of discrete elements of the accessory genome among bacterial strains in a population are needed to explore the relationships between the flexible genome and bacterial adaptive traits.

**Results:**

We have developed the open-source software package ClustAGE. This program, written in Perl, uses BLAST to cluster nucleotide accessory genomic elements from the genomes of multiple bacterial strains and to identify their distribution within the study population. The program output can be used in combination with strain phenotype data or other characteristics to detect associations. Optional graphical output is available for visualizing accessory genome gene content and distribution patterns. The capabilities of the software are demonstrated on a collection of 14 *Pseudomonas aeruginosa* genome sequences.

**Conclusions:**

The ClustAGE software and utilities are effective for identifying characteristics and distributions of accessory genomic elements among groups of bacterial genomes. The ability to easily and effectively characterize the accessory genome of a sequence collection may provide a better understanding of the accessory genome’s contribution to a species’ adaptation and pathogenesis. The ClustAGE source code can be downloaded from https://clustage.sourceforge.io and a limited web-based implementation is available at http://vfsmspineagent.fsm.northwestern.edu/cgi-bin/clustage.cgi.

**Electronic supplementary material:**

The online version of this article (10.1186/s12859-018-2154-x) contains supplementary material, which is available to authorized users.

## Background

Gene content can vary greatly between closely related strains of bacteria and between other unicellular organisms [1, 2]. Genes within a species can be divided into a conserved core genome and a flexible accessory genome. The core genome of an organism consists of genetic sequence that is conserved among all or nearly all members of the species. Conversely, the accessory genome represents genetic material that is present in some, but not all members of the species. The total complement of genetic material within a species is known as the pangenome [3]. Among bacteria, modification of gene content can arise from one of three major mechanisms: gene loss, gene gain through duplication, and gene gain through horizontal gene transfer (HGT) [4, 5]. Horizontally transferred genetic elements can include such structures as plasmids, integrative and conjugative elements (ICEs), replacement islands, prophages and phage-like elements, transposons, insertion sequences and integrons [6–8]. Collectively, these horizontally transferred elements, as well as any contiguous stretch of genetic material that is not part of the conserved core genome, regardless of source or structure, can be referred to as accessory genomic elements (AGEs).

The accessory genome of bacteria can be an important source of phenotypic diversity [9]. Genes within the accessory genome can drive environmental niche adaptation or pathogenesis within hosts [10, 11]. For instance, in *Pseudomonas aeruginosa*, genes within the accessory genome have been found to allow the organisms to persist in environments containing heavy metals and toxic organic compounds that would otherwise be unsuitable for *P. aeruginosa* habitation [12, 13]. In *Staphylococcus aureus,* the *S. aureus* pathogenicity islands (SaPIs) are a class of mobile genetic elements that carry genes encoding such virulence factors as TSST1, a toxin important in toxic shock syndrome, or other toxins [14]. Antibiotic resistance genes are frequently found in the accessory genomes of clinically important bacterial pathogens. One example is the carbapenemase-carrying plasmids in *Klebsiella pneumoniae* and other Gram-negative pathogens that contribute to the spread of this phenotype in healthcare settings [15, 16]. Given that bacterial accessory genomes are known to be enriched in virulence factors [17], directed study of the accessory genome contents and distributions within a population could yield new diagnostics and therapeutics for bacterial infections.

Often AGEs are not discrete structures with well-defined borders and gene compositions, but instead can be mosaic and fragmented with insertions of other elements, structural rearrangements, or partial deletions [18]. Mosaic islands have previously been described in *E. coli* [19] and *Streptococcus pneumoniae* [20]. In *Pseudomonas aeruginosa*, a genomic island containing the type 3 secretion system effector gene *exoU* was found to have extensive homology and synteny of genes in this island with genes in other *P. aeruginosa* islands PAPI-1 and pKLC102 [21]. Given the possibly mosaic nature of accessory genomic regions, accessory element characterization is often not as simple as screening genomes for a discrete set of defined genomic islands or other horizontally transferred elements. Therefore, a robust analysis of the pan-accessory genome of a set of bacterial strains must be able to account for potential changes in structure and composition of accessory regions between strains.

With the increase in availability and affordability of whole-genome sequencing, large-scale genomic analyses of populations of isolates have become more feasible. Software packages, such as mga [22] Mauve [23], and Mugsy [24], have been developed to perform segmented alignments of complete genomes for the purposes of aligning shared genomic regions. There are also several bioinformatic tools that exist to characterize the core and pangenome of bacterial species [25–28], but few that specifically examine the accessory genome fraction. To address the accessory genome of bacteria more directly, the previously presented bioinformatic tools Spine and AGEnt [29] were developed to identify the conserved nucleotide core genome sequence in a set of genomic sequences and use this core genome sequence to perform in silico subtractive hybridization to isolate the accessory genome component of each strain. However, software such as Spine and AGEnt or others [30] that characterize the accessory genome of bacterial strains do not focus directly on providing the distribution of accessory elements in a study population. Such distribution analyses are important for answering questions about the contributions of horizontally transferred or subgroup-specific genetic elements that may contribute, for example, to a particular phenotype of interest or to understanding particular niche adaptations.

This report describes ClustAGE, a software package that clusters accessory genomic elements identified by Spine and AGEnt from a set of genomes into discrete AGE units to define the distribution of accessory elements among the analyzed genomes. Several software tools such as DomClust [31], GCQuery [32], PanOCT [33] and OrthoDB [34] have been developed for the purposes of clustering gene sequences into orthologous groups. These programs identify clusters of related genes across bacterial genomes based on gene sequences. The approach to accessory genome characterization taken by ClustAGE differs from these other approaches in that ClustAGE compares the complete nucleotide sequences of the accessory genome rather than just the protein-coding sequences. A nucleotide-sequence-based, gene-agnostic approach offers several advantages in characterizing AGE distributions. First, the identification of shared accessory elements does not depend on annotation techniques, which may differ in technique and results between strains available from public databases or collaborators. Second, intergenic sequence distribution can be studied, allowing distributions of non-protein-coding sequences such as promoter sequences or small RNAs with potential biological relevance in the accessory genome of the population to more easily be analyzed. Third, this approach has the potential to capture variable regions within otherwise conserved genes that may have arisen by homologous recombination or other mechanisms. The data generated by this software allow detailed analysis of the flexible portion of a population’s pangenome.

## Implementation

ClustAGE is a command-line tool built using the Perl programming language for the purpose of analyzing and comparing accessory genomic elements (AGEs) between genomes. The source code is distributed as freeware under the GNU General Public License version 3. The core functionality of ClustAGE requires BLAST+ v2.3.0 [35, 36], of which binaries for OS X or Linux 64-bit are included with the distributions. Optional features require installation of the freeware programs gnuplot v5.0 (http://www.gnuplot.info/) and/or bwa v0.7.13 [37].

**Fig. 1:**
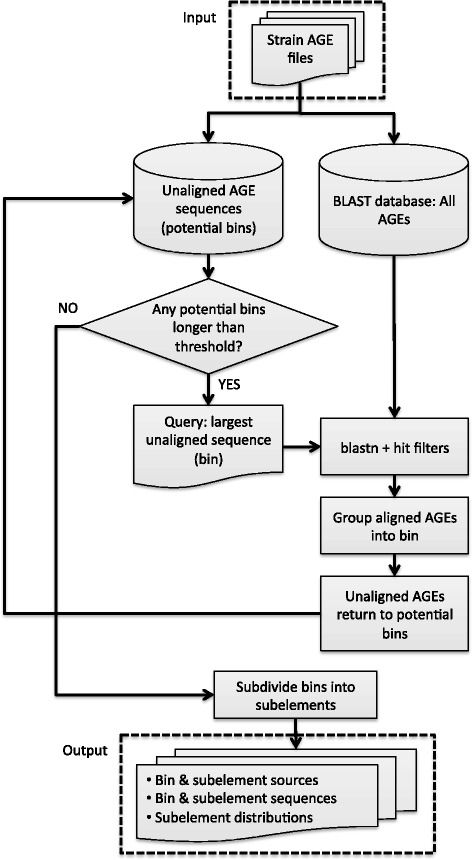
Schematic of ClustAGE clustering algorithm

ClustAGE takes as input sets of AGE nucleotide sequences previously identified from the genome sequences of at least two separate organisms. These AGE sequence sets can be extracted from complete or draft whole-genome nucleotide sequences using the previously-developed software tools Spine and AGEnt [29]. The ClustAGE algorithm identifies representative contiguous AGEs within the input data set and delineates the distribution of discrete AGEs among the strains’ genomes. It consists of two steps: defining “bins” and defining “subelements” (Fig. 1).

In the first step of this process, clustering of similar AGE sequences into “bins” is performed. First, AGE sequences from all genomes input into ClustAGE are pooled together to create a single nucleotide BLAST database. AGE sequences are then sorted by size. In the initial iteration of the clustering algorithm, the longest contiguous AGE in the dataset is chosen as a bin representative. This bin sequence is then used as the query sequence in a blastn alignment against the database of all input AGE sequences. Alignment results are filtered to remove any hits against AGEs from the same genome as the bin representative, as well as hits below user-defined sequence identity and length cutoffs. BLAST hits against AGE sequences that pass these filters are binned with the representative sequence and removed from the pool of potential bin representative sequences in subsequent iterations. Conversely, all non-aligning AGE sequences remain in the pool of potential bin representative sequences. If only part of an AGE sequence aligns to the bin representative, the non-aligning portion of the AGE sequence is isolated and added to the pool of potential bin representatives. Subsequent iterations of clustering select the next-longest complete or partial AGE sequence that was not previously binned with a larger bin representative sequence and uses it as the query sequence for alignment against the AGE sequence pool. Clustering iterations continue in this fashion until no bin representative sequences above a user-defined length threshold remain in the pool.

**Fig. 2:**
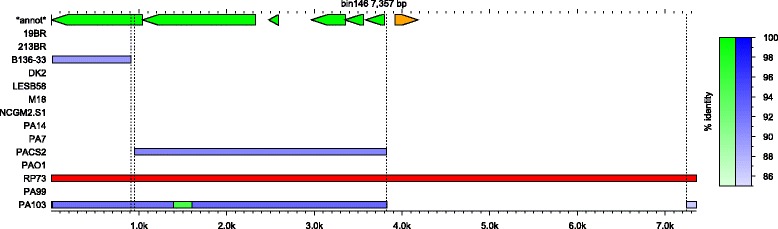
Example of AGE figure generated by ClustAGE. Top row labeled “*annot*” shows coding sequences previously annotated within the bin representative AGE with the strand on which the gene was found indicated by both color and arrow direction. The row with the red box indicates the strain that was the source of the bin representative sequence. Remaining rows are labeled with the source genome name and show the distribution of accessory element alignments against the bin representative AGE in blue. The intensity of color in each of the boxes corresponds to the percent nucleotide identity of the alignment according to the blue gradient in the legend on the right. The green box indicates AGE sequence that was not present in the assembled sequence of the PA103 strain, but for which alignments were found within the whole-genome sequence Illumina read set. Color intensity of the read-aligned portions corresponds to percent nucleotide identity of the alignment according to the green gradient in the legend at right. Vertical dashed lines show subelement borders. Lines dividing subelements smaller than 20 bp are not shown

Once AGE bins are defined, they are further subdivided into discrete units referred to as “subelements”. Bins are divided into subelements between positions on the reference AGE where the set of input genomes aligning to the reference AGE at the base or bases before the division differs from the set of genomes aligning to the reference AGE base or bases after the position (Fig. 2). In other words, a subelement represents the longest stretch of nucleotide sequence within the bin representative that is contiguous in all strains that contain it. By dividing AGEs into discrete subelements, insertions and deletions contributing to the mosaic nature of genomic islands and other horizontally transferred elements can be identified [38, 39].

Output files from the core function of ClustAGE described above include nucleotide sequences of the bin representative and nucleotide sequences of AGE subelements longer than a user-defined cutoff. A file listing positions within the input sequences from which the bin representative AGEs were derived, as well as a file listing the positions of subelements within each AGE and the distributions of each subelement among the input sequences are also output. Optionally, ClustAGE can produce plots of AGE distributions among the input genomes for each of the bin representative AGEs (Fig. 2). This functionality requires gnuplot (http://www.gnuplot.info) to produce the plots.

ClustAGE allows users to include coordinates and descriptions of protein coding sequences (CDS) within accessory elements as input. If provided, information about which coding sequences are contained within bins and subelements is output for each AGE for which annotations in the bin reference sequence were given. If graphical output was requested, annotated gene positions and directionality will be shown in the images (Fig. 2).

One limitation of working with draft genome sequences generated by de novo assembly of short sequencing reads such as those produced by Illumina sequencing technology is that the assembly process can fail to assemble small portions of the genome even when sufficient reads covering these regions are present in the read data set. This in turn can lead to the false presumption that an AGE is absent from a genome when in fact it simply failed to be properly assembled. To try to account for data missing from de novo generated draft genome sequences assembled from Illumina reads, ClustAGE includes an option to identify missing AGE sequences from raw read sequences. After the set of AGEs is identified from accessory genome sequences as detailed above, whole-genome Illumina sequencing data provided to ClustAGE is aligned to the bin reference AGE sequences using the ‘mem’ function of bwa aligner with default settings [37]. To try to minimize false-positive alignments of core genome read sequences to accessory regions, a core genome nucleotide sequence, such as output by Spine [29], can be provided to ClustAGE. Any reads aligning to both the core genome sequence and an AGE bin sequence will be excluded. Reads aligning to AGEs above a user-defined minimum depth of coverage and producing a contiguous alignment exceeding a minimum user-defined sequence similarity will be added to the binned sequence for that genome. Alignment data from Illumina reads are only added in AGE regions that were not found to have alignments against a bin representative AGE in the original input draft sequence for a genome. To minimize false-positive results, read alignment data are also not added unless the alignment region is either at one or both of the bin representative AGE ends or contiguous with accessory genomic sequence previously aligned by BLAST from assembly data. Subelements are then redefined using the added read alignment data and a separate set of “read-corrected” subelement sequence and coordinate files are output. If optional plotting of AGE distributions was chosen, read-aligned AGE regions are plotted using a different color to distinguish them from AGE alignments derived from accessory genomic sequences (Fig. 2).

The ClustAGE results can be used to visualize and compare relative similarity of total accessory genome content among strains in the population studied. The pipeline script subelements_to_tree.pl is provided with ClustAGE for this purpose. The program quantifies relative amount of shared subelement accessory genomic sequence for each pair of genomes by calculating Bray-Curtis distances [40]. Briefly, the Bray-Curtis distance for a pair of genomes is calculated as *d* = 1 – (2 *S*_*ij*_ / (*S*_*ii*_ + *S*_*jj*_)) where *S*_*ij*_ is the total length, in bases, of subelements identified by ClustAGE in both genomes *i* and *j* and *S*_*ii*_ and *S*_*jj*_ are the total accessory genome subelement sizes, in bases, of genomes *i* and *j*, respectively. In order to cluster strains by total accessory genome similarity, a matrix of Bray-Curtis distances for each pair of input strains is used to create a neighbor-joining tree using the ‘neighbor’ function of PHYLIP version 3.696 [41]. Optional bootstrap trees from random re-samplings of the data can be generated using PHYLIP’s ‘seqboot’ and ‘neighbor’ functions. Bootstrap support values can then be calculated for each branch of the neighbor-joining tree using the CompareToBootstrap.pl script developed by Morgan N. Price (http://microbesonline.org/fasttree/treecmp.html). In addition to the neighbor-joining tree, a matrix of Bray-Curtis similarity values (1 – *d*) is output, as well as a file that can be used to add a heatmap of Bray-Curtis similarity values to the neighbor-joining tree in the online tree visualization software Interactive Tree Of Life (http://itol.embl.de) [42].

A utility for visualizing ClustAGE results as a pan-accessory genome figure is also available. ClustAGE Plot (http://vfsmspineagent.fsm.northwestern.edu/cgi-bin/clustage_plot.cgi) uses CGView [43] to produce a representation of ClustAGE results as bins ordered largest to smallest in a circular configuration with concentric rings indicating the distributions of accessory elements for each included strain. Although designed to be flexible, user-friendly, and powerful enough for most users, visualizations with ClustAGE Plot could become less informative with larger (i.e > 100 genomes) and/or high complexity data sets. The xml-formatted file produced by ClustAGE Plot can be downloaded and used to produce higher resolution images on a user’s local version of CGView. Furthermore, the output files generated by ClustAGE provide sufficient data for further processing and can be reformatted to serve as input for other applications capable of visualizations such as R (https://www.r-project.org/), Circos (http://circos.ca/), or other 3rd party programs, depending on the users’ needs and skills.

The ClustAGE scripts and utilities are available for download at https://clustage.sourceforge.io. A web-based implementation of ClustAGE is also available at http://vfsmspineagent.fsm.northwestern.edu/cgi-bin/clustage.cgi. The web version is limited to a maximum of 15 accessory genome sequence sets and does not support read-correction of AGEs.

## Results and discussion

### Data set

To demonstrate the functionality of ClustAGE, Spine v0.2.1 was used to identify the core and accessory genomic sequences of a set of 12 *Pseudomonas aeruginosa* strains, as described previously [29]. The 12 strain sequences used and their NCBI accession numbers were 19BR (AFXJ01000001.1), 213BR (AFXK01000001.1), B136-33 (CP004061.1), DK2 (CP003149.1), LESB58 (FM209186.1), M18 (CP002496.1), NCGM2.S1 (AP012280.1), PA7 (CP000744.1), UCBPP-PA14 (CP000438.1), PACS2 (NZ_AAQW01000001.1), PAO1 (AE004091.2), and RP73 (CP006245.1). Using a core genome definition of sequences present in at least 11 of the 12 reference genomes, the reference core genome size was 5844 kbp. AGEnt v0.2.1 was then used to determine the accessory genomic sequences of these 12 strains as well as of two draft genome assemblies of *P. aeruginosa* strains, PA99 (JARJ01000000) and PA103 (JARI01000000). The average total size of the accessory genomic fraction of a strain was 735 kbp (range 428 kbp - 1177 kbp) with an average of 208 contiguous accessory elements (range 170 - 435). Output files from the Spine and AGEnt analyses are available in Additional file 1. Sequencing read sets for PA99 and PA103 consisting of 100 bp paired-end Illumina reads generated by the HiSeq 2000 platform are available from the NCBI short read archive (SRR5447413 and SRR5447414). For more detail on the derivation and characteristics of the core and accessory genomes of this sequence set, see previous publication on Spine and AGEnt [29].

### Performance

ClustAGE was first run on this dataset using the default settings of a minimum of 85% nucleotide sequence identity across a blast hit, a minimum hit length of 100 bp, and a minimum bin representative size of 200 bp. ClustAGE output files are provided in Additional file 2. A total of 2907 individual sequences were present among the accessory genomes of the 14 input genomes ranging in size from 10 bp to 127,886 bp. Among these elements, 1959 were at least 200 bp in length. After the BLAST clustering step, 952 bin representative sequences were identified. As represented by these AGE sequences, the total unique accessory sequence at least 200 bp in length among these 14 genomes was 4,207,472 bp with an average bin length of 4420 bp (Table 1). An average of 68 AGEs or partial AGEs from each genome served as bin representative AGEs (range 17 – 270 AGEs) with an average cumulative bin size of 300,534 bp per strain (range 39,720 – 897,494 bp). At the conclusion of the binning step, 99.01% of the total input accessory sequence of all 14 strains was aligned within one of the 952 bin representatives (Additional file 3). Among those sequences that were not binned, the median length of an unbinned segment was 41 bp with a range of 1 to 196 bp. This indicates that sequences excluded from binning were primarily short regions that were unable to be properly aligned by BLAST and/or unique regions that were smaller than the 200 bp minimum bin size cutoff.

**Table 1:** AGE bin representative characteristics

Strain	# bin representatives	Total size of bin representatives, in bp	Average bin representative size, in bp (min - max)
19BR	109	377,039	3459 (216 - 50,833)
213BR	24	129,277	5387 (268 - 54,765)
B136-33	67	224,291	3348 (227 - 15,557)
DK2	56	241,012	4304 (226 - 81,418)
LESB58	58	353,435	6094 (206 - 50,121)
M18	53	170,952	3226 (206 - 31,798)
NCGM2.S1	82	470,335	5736 (270 - 40,043)
PA14	46	245,206	5331 (209 - 127,886)
PA7	270	897,494	3324 (208 - 21,861)
PACS2	49	228,811	4670 (200 - 55,310)
PAO1	17	39,720	2336 (229 - 63,512)
RP73	29	136,895	4721 (227 - 32,463)
PA99	46	279,864	6084 (217 - 10,474)
PA103	46	413,141	8981 (212 - 46,125)
Total	952	4,207,472	–
Average	68	300,534	–

**Fig. 3:**
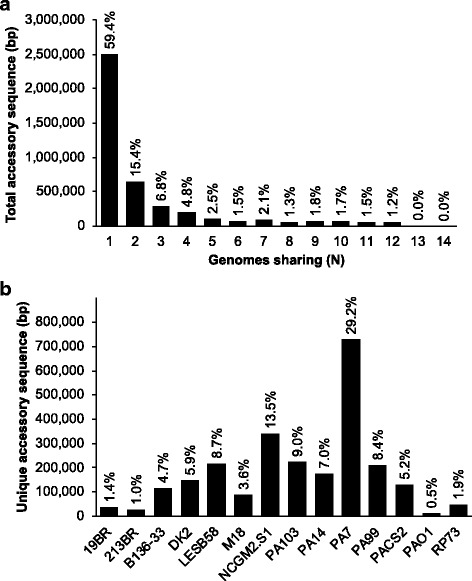
AGE subelement sequence distribution. **a** Amount of total subelement sequence, in bp, shared among the number of genomes indicated along the x-axis. Bars are labeled with the percent of the total subelement sequence among all input strains shared by the given number of strains. **b** Amount of total unique subelement sequence, in bp, found in only one of the fourteen genomes by genome ID. Bars are labeled with the percent of total unique subelement sequence among all input strains found within the indicated strain

Alignments against bin representative AGEs were further subdivided at positions where the set of genomes with elements aligning to the bin representative before the position differed from the set aligning after the position. In this fashion, the 952 bin representative AGEs were subdivided into 2346 discrete subelements with an average of 2.5 subelements per AGE (range 1 – 120 subelements per AGE). The average subelement size was 1793 bp (range 1 – 40,966 bp). This demonstrates the mosaic nature of many *P. aeruginosa* AGEs with horizontal transfer of sections of AGEs rather than as discrete islands or interruption of AGEs in the genome with newly-acquired AGEs. Among the accessory genomes of these 14 strains, the majority of the sequence was unique with 59.5% of all subelement sequence found in only one genome (Fig. 3a). Strain PA7 had the largest share of unique AGE sequence with 29.2% of all unique subelement sequence (Fig. 3b). It has been previously shown that PA7 is an outlier strain among *P. aeruginosa* species based on comparisons of multi-locus sequence type (MLST) gene sequences and syntenous regions of other strains [44]. These results suggest that the accessory genome composition of PA7 is also dissimilar compared to other *P. aeruginosa* strains.

**Fig. 4:**
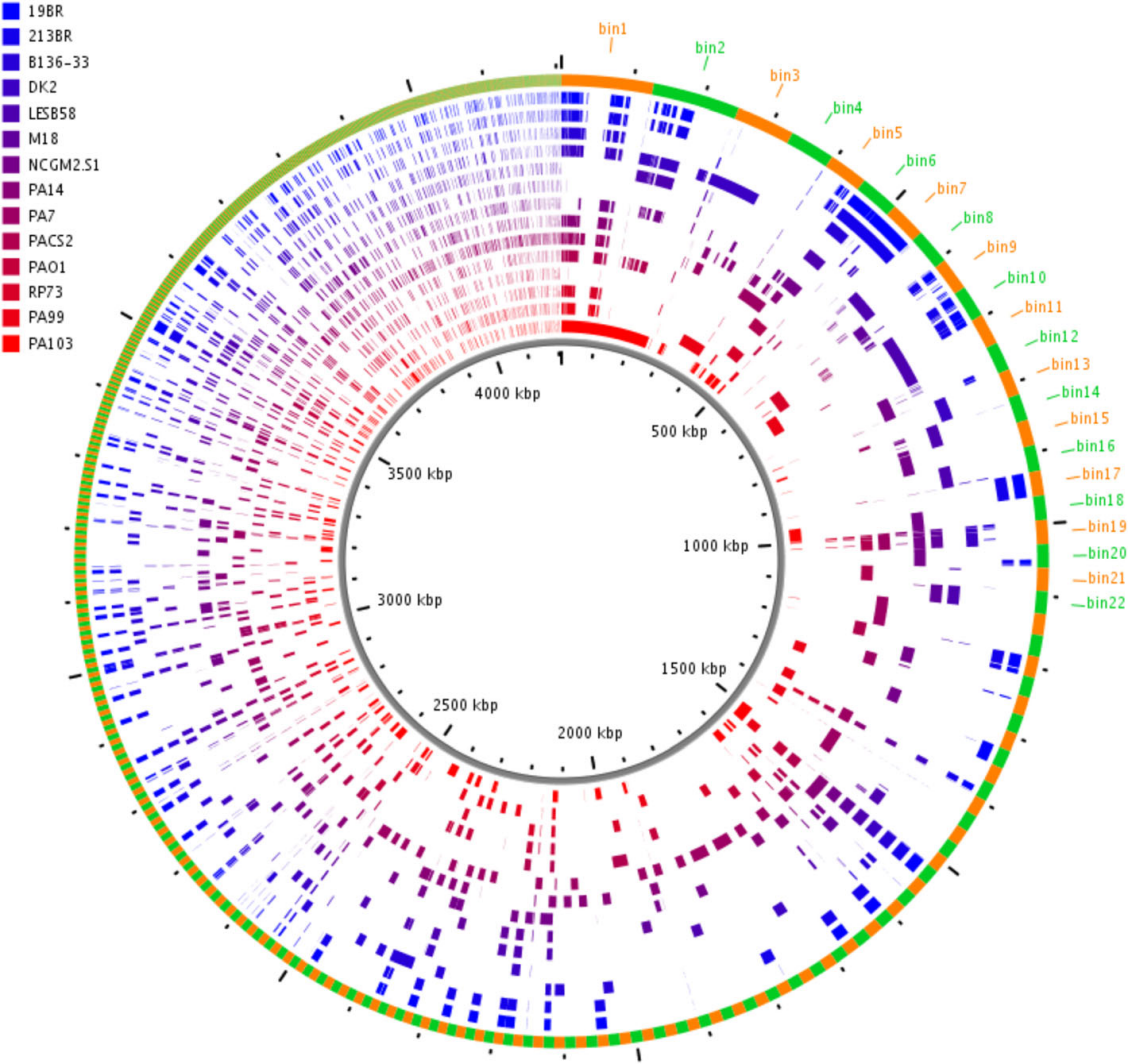
Pan accessory genome distribution among 14 *P. aeruginosa* isolates. Outer ring shows ClustAGE bins at least 200 bp in size ordered clockwise from largest to smallest with alternating orange and green colors to indicate bin borders. Concentric inner rings show distributions of accessory elements within each strain. Ruler in the center of the figure indicates the cumulative size of the accessory genome bin representatives in kilobases. Figure generated using ClustAGE Plot utility available at http://vfsmspineagent.fsm.northwestern.edu/cgi-bin/clustage_plot.cgi

Illumina short sequencing reads were used to extend AGEs for the two draft genome sequences of PA99 and PA103. This added 2080 bp of sequence to the 722,954 bp of subelement sequence in the draft genome sequence of PA99 for an increase of 0.3% and added 5306 bp of sequence to the 944,716 bp of subelement sequence in the draft genome sequence of PA103 for an increase of 0.6% (Table 2). In total, sequence derived from strain PA99 read alignments was added to 45 bins with an average of 46 bp of sequence added per bin (range 1 – 350 bp) and sequence derived from PA103 read alignments was added to 60 bins with an average of 100 bp of sequence added per bin (range 1 – 1247 bp). With the additional sequence extension of the AGEs for strains PA99 and PA103, the 952 AGE bins were divided into 2382 discrete subelements. Subelement characteristics were similar to non-read-corrected subelements with an average of 2.5 subelements per AGE bin (range 1 – 122) and an average subelement size of 1766 bp (range 1 – 40,966 bp). A representation of distribution of the total accessory genome of the 14 strains in bins at least 200 bp in length is shown in Fig. 4.

**Table 2:** AGE read correction results per strain

	PA99	PA103
Total added accessory genome sequence (bp)	2080	5306
% increase in total accessory genome length	0.30%	0.60%
# bins with added sequence	45	60
Average bp added per bin (min - max)	46 (1 - 350)	100 (1 - 1247)

To examine the effect of modifications to the default settings of ClustAGE on output, the analysis was repeated with a more permissive minimum sequence identity of 80%, as well as a more restrictive minimum sequence identity of 90%. See Additional file 4 for a table comparing ClustAGE results at the different cutoffs. Using a setting of 80% minimum sequence identity, there were more bin representatives identified comprising less total sequence and more subelement divisions of the bin representatives compared to when the default setting of 85% was used. The lower sequence identity threshold results in more alignments against bin representatives being preserved. This causes more binning of portions of AGEs within the potential bin representative pool leaving more unbinnned AGE fragments to serve as bin representatives. This is reflected in the decreased average length of the bin representatives compared to the results of the 85% cutoff. This also leads to greater fragmentation of the AGEs into subelements as more potentially nonspecific BLAST alignments escape filtering. Conversely, the more restrictive 90% sequence identity cutoff resulted in fewer AGE representatives of longer average length divided into fewer total subelements. Further comparisons of ClustAGE results after read correction can be seen in the table in Additional file 4.

**Fig. 5:**
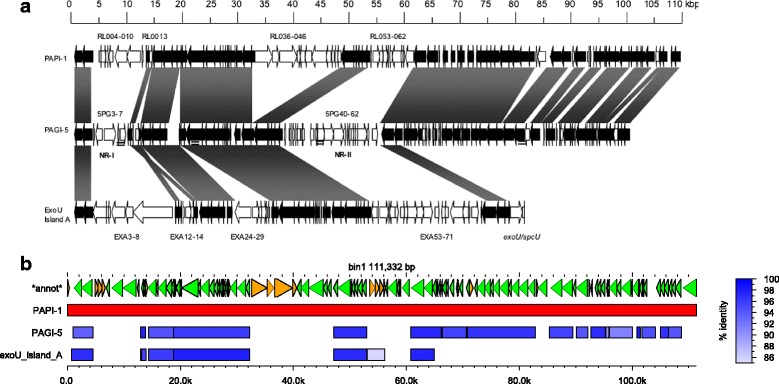
Genomic island variability. **a** Alignment of *P. aeruginosa* genomic islands PAGI-5, PAPI-1, and ExoU Island A. Dark bands and ORFs represent conserved nucleotide sequences. Figure reproduced from Battle et al. (10.1128/JB.00785-08) [45] with permission from the American Society for Microbiology. **b** Graphical output from ClustAGE analysis of the same three genomic islands. Arrows in the top row correspond to coding sequences on the forward (orange) and reverse (green) strands of the bin representative PAPI-1. PAPI-1, as the longest of the three genomic island sequences, is shown in red in the next row. Alignments of PAGI-5 and exoU Island A against PAPI-1 are shown in blue. Percent sequence similarity of the alignments is represented by shade of blue according to the legend at right

The ability of ClustAGE to identify mosaicism in AGEs, i.e. insertions and/or deletions within larger AGE structures, was tested using a set of previously-described related genomic islands in *P. aeruginosa*. Sequences of genomic islands PAPI-1 (Genbank accession AY273869.1), ExoU island A (accession DQ437742.1), and PAGI-5 (accession EF611301.1) were downloaded from NCBI GenBank. These AGEs have been previously identified as related hybrid genomic islands [45]. ClustAGE analysis of these three AGEs recaptured the previously-described mosaic nature the genomic islands (Fig. 5). Similar to what has been previously reported, ClustAGE again showed that PAGI-5 is missing three large genomic regions relative to PAPI-1 as well as several smaller regions. Moreover, ClustAGE was also able to identify a region spanning bases 53,059 – 56,162 in PAPI-1 that contains 4 genes with sequence similarity to a region in exoU island A that is not present in PAGI-5. These results demonstrate that ClustAGE is able to accurately identify insertions and deletions in AGEs that are consistent with the mosaic nature of the accessory genome in *P. aeruginosa*.

### ClustAGE gene distribution

ClustAGE differs from gene-based approaches to accessory genome characterization in that it identifies the distribution of nucleotide accessory genomic element regions independent of the presence or absence of discrete coding regions within those elements. To evaluate the performance of ClustAGE in determining the presence or absence of accessory elements among the included strains, ClustAGE output was compared with ortholog determinations between coding sequences in the annotated accessory genomes using reciprocal best BLAST hit (RBB) analysis [46, 47] (Methods in Additional file 5). Briefly, for each previously-annotated gene in each ClustAGE bin reference sequence, if an accessory nucleotide sequence for one of the 13 query genomes was aligned to the region of the bin reference sequence in which the gene was annotated, for the purposes of comparison that gene was considered present in the query genome. Conversely, genes in the bin reference not covered by an alignment were considered absent. These results were compared to RBB analysis results between annotated genes in the accessory genome fractions of all 14 included strains. To account for potential differences in annotation approaches between the genomes that could have resulted in either over-calling or under-calling potential coding sequences in some genomes, instances where a bin reference gene was identified as present in a query genome by ClustAGE, but no RBB ortholog was present in the query genome were confirmed by translated blast analysis (tblastn) of the bin reference protein sequence against the nucleotide sequence of the accessory genome fraction of the query genome. Results showed 98.18% concordance between ClustAGE results and RBB results (Table 3). These findings indicate that ClustAGE is effective and accurate in identifying the presence or absence of regions containing gene orthologs. Further discussion of methods and results can be found in Additional file 5 and the detailed results can be seen in the table in Additional file 6.

**Table 3:** ClustAGE annotation vs. gene ortholog analysis

	# comparisons	% of comparisons
Concordant	41,609	98.18%
ClustAGE+ / Ortholog-^a^	328	0.77%
ClustAGE- / Ortholog+	443	1.05%

Comparisons of determinations of gene presence or absence based on ClustAGE alignments to determination of orthologous genes based on reciprocal best blast hit (RBB) analysis of annotated genes in the accessory genomes of each strain. Minimum ClustAGE nucleotide alignment percent identity = 85%. Minimum RBB percent identity = 85%

^a^Genes identified by ClustAGE but not by RBB were counted as present if tblastn analysis identified the gene in accessory genome sequence with at least 50% coverage by length and 85% sequence identity

### Accessory genome similarity

**Fig. 6:**
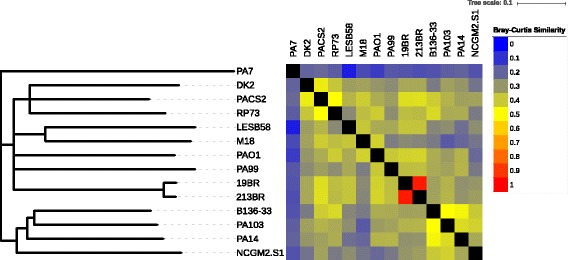
Relative amount of shared accessory genome content among 14 *P. aeruginosa* isolates. Bray-Curtis distances (*d*) were calculated for every pair-wise comparison of shared AGE content between strains. Neighbor-joining tree (left) is a consensus across 1000 bootstrap resamplings of AGE distributions. Branches with support < 500 were collapsed. Heatmap (right) shows relative pairwise AGE content similarity (1 - *d*) between strains

Using the sublement_to_tree.pl utility included with ClustAGE, the similarity of accessory element contents between strains in the dataset was evaluated. Bray-Curtis distances based on presence of subelements at least 100 bp in length were calculated for each pair of genomes and used to produce a neighbor-joining tree with 1000 bootstrap replicates (Fig. 6). The relative amount of shared accessory genome sequence between pairs of strains was calculated from Bray-Curtis distances and used to generate a heat map of relative accessory genome similarity. This analysis showed that the accessory genomes of strains 19BR and 213BR were nearly identical. It also showed that the genome of PA7 shared little accessory genome with the other genomes studied here, consistent with its status as a taxonomic outlier strain [44].

### Scalability and computational efficiency

As the cost of microbial whole-genome sequencing has decreased and availability of sequencing resources has increased, computational requirements for analyzing the resulting genomic data sets can become a limiting factor. Processing time and memory requirements of ClustAGE analyses were evaluated using AGE data sets from increasing numbers of genomes. The figure in Additional file 7 shows the average analysis time and average maximum memory requirements for ClustAGE analyses. Five replicate analyses of each number of input genomes were conducted on both a server platform running Ubuntu Linux as well as a desktop computer running Mac OS X. For more details, see Additional Methods in Additional file 5. On both computing platforms the ClustAGE processing times increased linearly up to 200 genomes, with r-squared values of 0.9906 and 0.9925 on the Linux and OS X platforms, respectively. The average time required to analyze 200 accessory genomes was less than 70 min on both computers. Peak memory use also increased linearly up to 200 genomes analyzed with a maximum average RAM use of 1.6 Gb on the Ubuntu Linux server and 1.2 Gb on the Mac OS X desktop computer. It is expected that processing time and memory use requirements are likely to vary further depending on average accessory genome size of the analyzed strains. Nonetheless, these results indicate that ClustAGE analysis is scalable to larger genome data sets and suggest that users without access to high-memory and/or multiple processor computing resources can still perform ClustAGE analyses on AGEs derived from 10s or 100 s of genomic sequences using standard desktop or even laptop computers.

## Conclusions

ClustAGE, in combination with the core and accessory genome identification packages Spine and AGEnt [29], is an easy-to-use and accurate software tool to characterize the distribution of accessory genomic elements (AGEs) within a collection of bacterial whole-genome sequences. It includes utilities for visualizing AGE distributions and comparing and classifying relative accessory genome similarity among strains in the studied population. Taken together, the analysis output provided by ClustAGE can offer researchers a powerful new tool to study the relationships of discrete strain characteristics with flexible genome content in large genomic data sets to gain insight into bacterial evolution and adaptation.

## Availability and requirements

Project name: ClustAGE.

Project home page: https://sourceforge.net/projects/clustage and http://vfsmspineagent.fsm.northwestern.edu/cgi-bin/clustage.cgi.

Operating system(s): Platform independent.

Programming language: Perl.

Other requirements: Perl 5.10 or higher, BLAST+ 2.3.0 or higher. For optional functions, gnuplot 5.0 or higher, bwa 0.7.13 or higher, and/or phylip 3.695 or higher are necessary.

License: GNU GPL v3.

Any restrictions to use by non-academics: None.

## Additional files

Additional file 1: Archive containing relevant output files from the Spine and AGEnt analyses of the reference genomes. (ZIP 4849 kb)

Additional file 2: Archive containing output files from ClustAGE analysis of accessory genome sequence files found in Additional file 1. (ZIP 18100 kb)

Additional file 3: Unbinned accessory sequences. (XLSX 52 kb)

Additional file 4: ClustAGE output characteristics. (XLSX 58 kb)

Additional file 5: ClustAGE gene distribution analysis. (DOCX 33 kb)

Additional file 6: Comparison of ClustAGE results with pairwise gene ortholog analysis. (XLSX 54 kb)

Additional file 7: ClustAGE computational performance. Randomly selected sets of accessory genomic elements from identified from *Pseudomonas aeruginosa* whole genome sequences were analyzed by ClustAGE. Analyses were performed on a server platform running Ubuntu (Linux, blue) and on a desktop computer running OS X (Mac, orange). Time to completion of ClustAGE analysis (solid lines) and maximum memory usage (dashed lines) were measured for each analysis. Each point represents the average of 5 replicate analyses at each number of input genomes. Error bars represent the standard error of the mean. (PDF 72 kb)
