# Supplementary figures and images for: ClustAGE: a tool for clustering and distribution analysis of bacterial accessory genomic elements (part 2 of 2)
Source: BMC Bioinformatics. 2018 Apr 20;19:150. doi: 10.1186/s12859-018-2154-x (PMC5910555; doi:10.1186/s12859-018-2154-x)

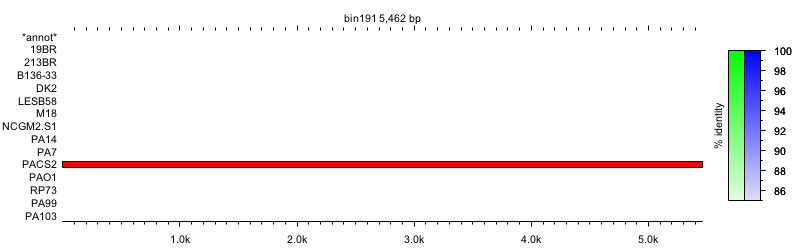

Supplement: Supplementary file 2 — Archive containing output files from ClustAGE analysis of accessory genome sequence files found in Additional file 1. (ZIP 18100 kb) [file 12859_2018_2154_MOESM2_ESM.zip › PA_14genomes_clustage_graphs/bin191_PACS2.png]

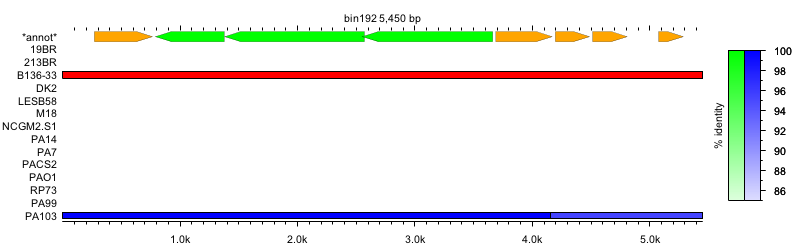

Supplement: Supplementary file 2 — Archive containing output files from ClustAGE analysis of accessory genome sequence files found in Additional file 1. (ZIP 18100 kb) [file 12859_2018_2154_MOESM2_ESM.zip › PA_14genomes_clustage_graphs/bin192_B136-33.png]

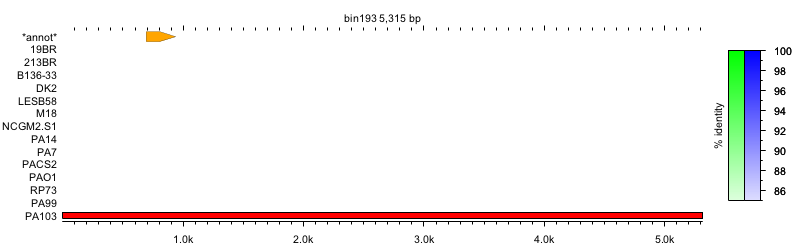

Supplement: Supplementary file 2 — Archive containing output files from ClustAGE analysis of accessory genome sequence files found in Additional file 1. (ZIP 18100 kb) [file 12859_2018_2154_MOESM2_ESM.zip › PA_14genomes_clustage_graphs/bin193_PA103.png]

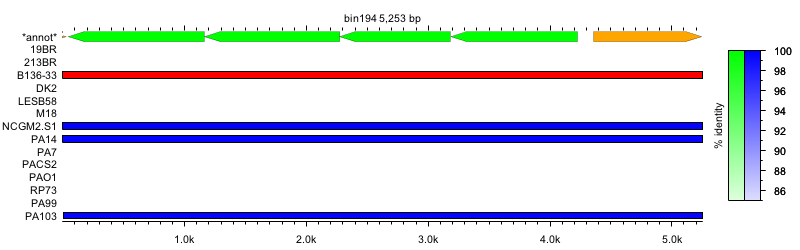

Supplement: Supplementary file 2 — Archive containing output files from ClustAGE analysis of accessory genome sequence files found in Additional file 1. (ZIP 18100 kb) [file 12859_2018_2154_MOESM2_ESM.zip › PA_14genomes_clustage_graphs/bin194_B136-33.png]

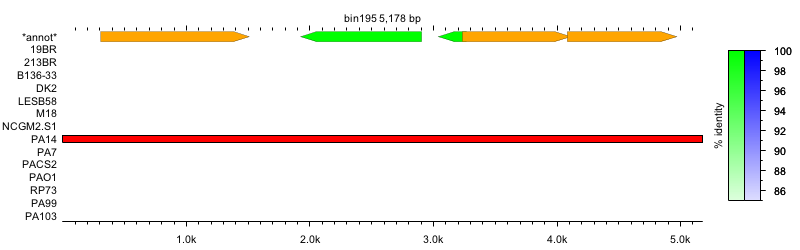

Supplement: Supplementary file 2 — Archive containing output files from ClustAGE analysis of accessory genome sequence files found in Additional file 1. (ZIP 18100 kb) [file 12859_2018_2154_MOESM2_ESM.zip › PA_14genomes_clustage_graphs/bin195_PA14.png]

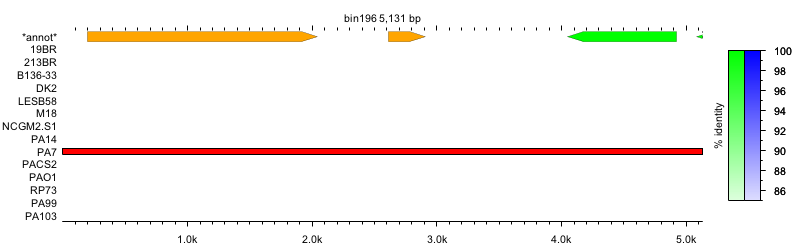

Supplement: Supplementary file 2 — Archive containing output files from ClustAGE analysis of accessory genome sequence files found in Additional file 1. (ZIP 18100 kb) [file 12859_2018_2154_MOESM2_ESM.zip › PA_14genomes_clustage_graphs/bin196_PA7.png]

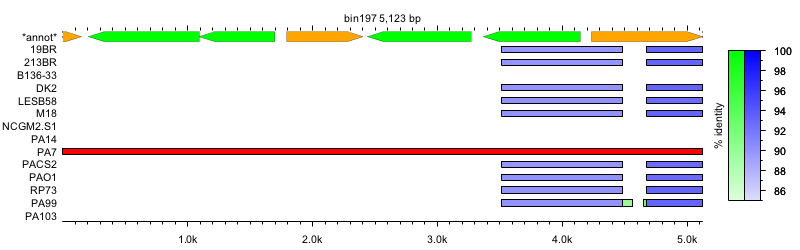

Supplement: Supplementary file 2 — Archive containing output files from ClustAGE analysis of accessory genome sequence files found in Additional file 1. (ZIP 18100 kb) [file 12859_2018_2154_MOESM2_ESM.zip › PA_14genomes_clustage_graphs/bin197_PA7.png]

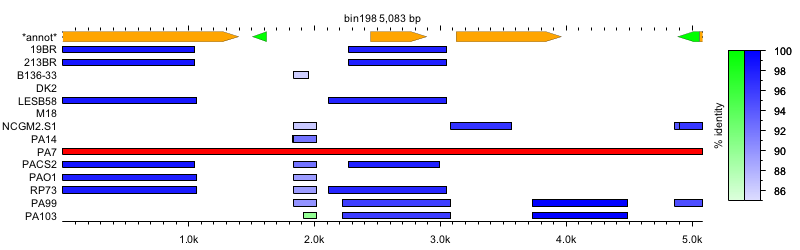

Supplement: Supplementary file 2 — Archive containing output files from ClustAGE analysis of accessory genome sequence files found in Additional file 1. (ZIP 18100 kb) [file 12859_2018_2154_MOESM2_ESM.zip › PA_14genomes_clustage_graphs/bin198_PA7.png]

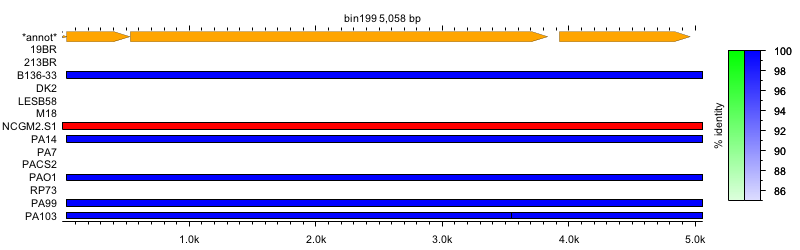

Supplement: Supplementary file 2 — Archive containing output files from ClustAGE analysis of accessory genome sequence files found in Additional file 1. (ZIP 18100 kb) [file 12859_2018_2154_MOESM2_ESM.zip › PA_14genomes_clustage_graphs/bin199_NCGM2.S1.png]

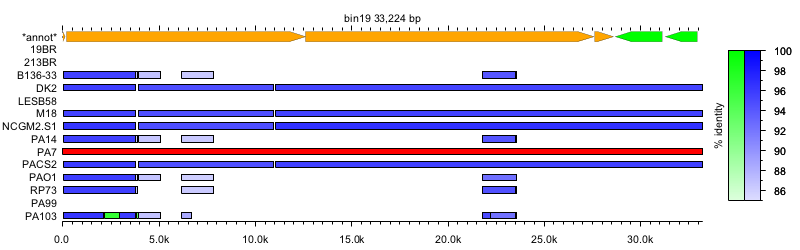

Supplement: Supplementary file 2 — Archive containing output files from ClustAGE analysis of accessory genome sequence files found in Additional file 1. (ZIP 18100 kb) [file 12859_2018_2154_MOESM2_ESM.zip › PA_14genomes_clustage_graphs/bin19_PA7.png]

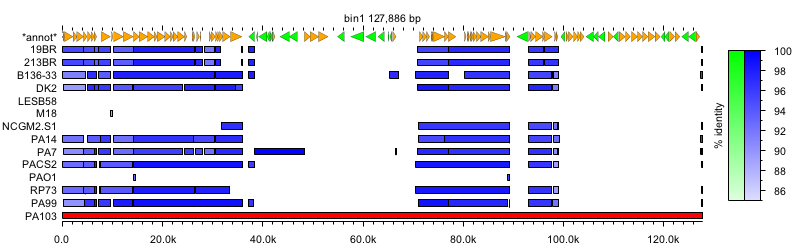

Supplement: Supplementary file 2 — Archive containing output files from ClustAGE analysis of accessory genome sequence files found in Additional file 1. (ZIP 18100 kb) [file 12859_2018_2154_MOESM2_ESM.zip › PA_14genomes_clustage_graphs/bin1_PA103.png]

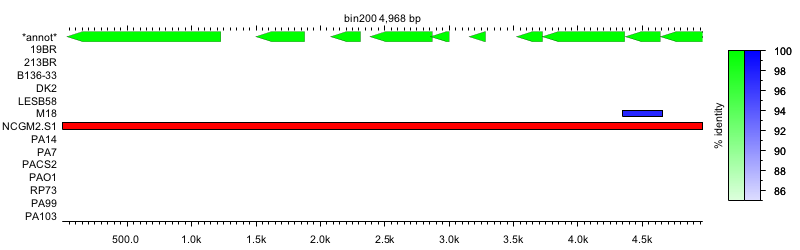

Supplement: Supplementary file 2 — Archive containing output files from ClustAGE analysis of accessory genome sequence files found in Additional file 1. (ZIP 18100 kb) [file 12859_2018_2154_MOESM2_ESM.zip › PA_14genomes_clustage_graphs/bin200_NCGM2.S1.png]

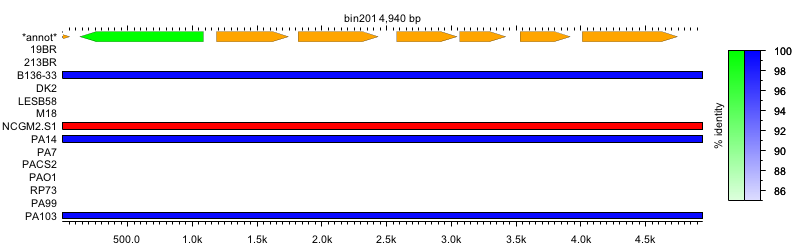

Supplement: Supplementary file 2 — Archive containing output files from ClustAGE analysis of accessory genome sequence files found in Additional file 1. (ZIP 18100 kb) [file 12859_2018_2154_MOESM2_ESM.zip › PA_14genomes_clustage_graphs/bin201_NCGM2.S1.png]

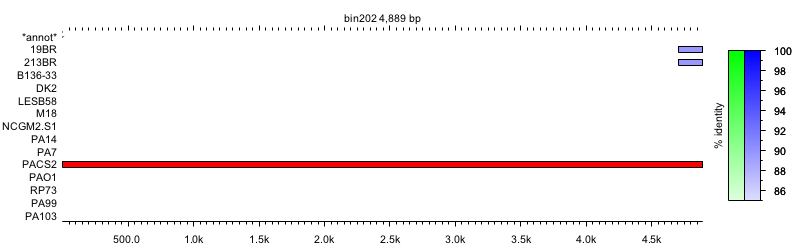

Supplement: Supplementary file 2 — Archive containing output files from ClustAGE analysis of accessory genome sequence files found in Additional file 1. (ZIP 18100 kb) [file 12859_2018_2154_MOESM2_ESM.zip › PA_14genomes_clustage_graphs/bin202_PACS2.png]

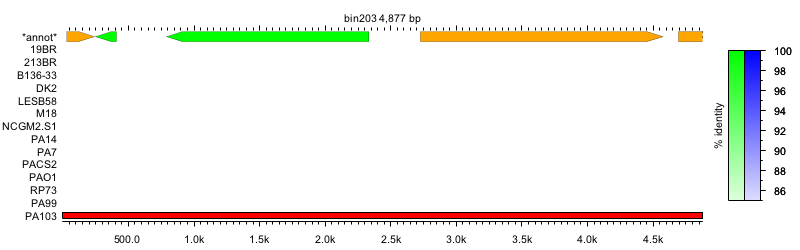

Supplement: Supplementary file 2 — Archive containing output files from ClustAGE analysis of accessory genome sequence files found in Additional file 1. (ZIP 18100 kb) [file 12859_2018_2154_MOESM2_ESM.zip › PA_14genomes_clustage_graphs/bin203_PA103.png]

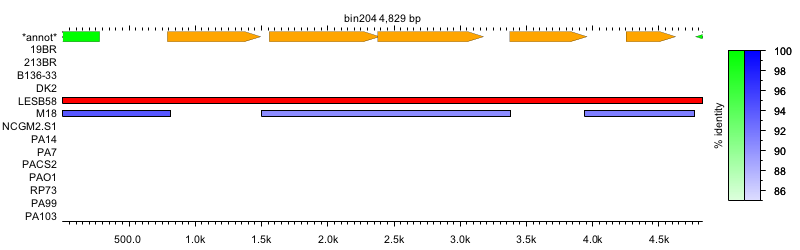

Supplement: Supplementary file 2 — Archive containing output files from ClustAGE analysis of accessory genome sequence files found in Additional file 1. (ZIP 18100 kb) [file 12859_2018_2154_MOESM2_ESM.zip › PA_14genomes_clustage_graphs/bin204_LESB58.png]

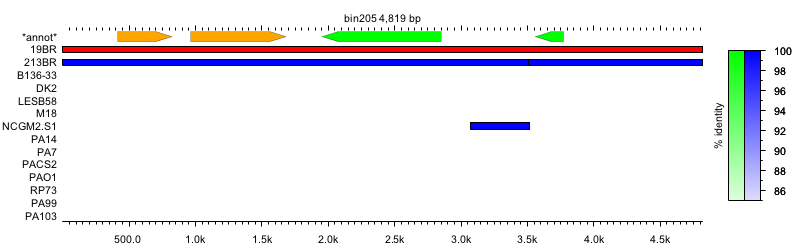

Supplement: Supplementary file 2 — Archive containing output files from ClustAGE analysis of accessory genome sequence files found in Additional file 1. (ZIP 18100 kb) [file 12859_2018_2154_MOESM2_ESM.zip › PA_14genomes_clustage_graphs/bin205_19BR.png]

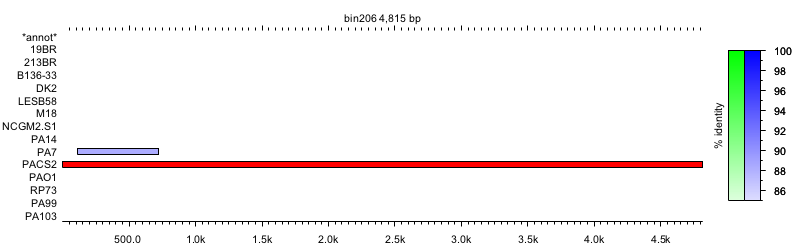

Supplement: Supplementary file 2 — Archive containing output files from ClustAGE analysis of accessory genome sequence files found in Additional file 1. (ZIP 18100 kb) [file 12859_2018_2154_MOESM2_ESM.zip › PA_14genomes_clustage_graphs/bin206_PACS2.png]

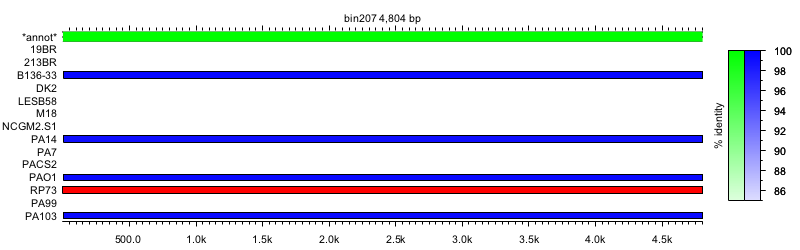

Supplement: Supplementary file 2 — Archive containing output files from ClustAGE analysis of accessory genome sequence files found in Additional file 1. (ZIP 18100 kb) [file 12859_2018_2154_MOESM2_ESM.zip › PA_14genomes_clustage_graphs/bin207_RP73.png]

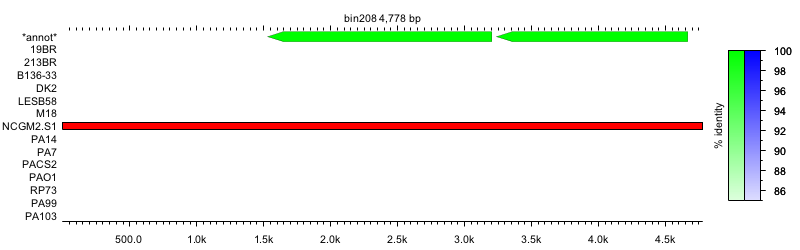

Supplement: Supplementary file 2 — Archive containing output files from ClustAGE analysis of accessory genome sequence files found in Additional file 1. (ZIP 18100 kb) [file 12859_2018_2154_MOESM2_ESM.zip › PA_14genomes_clustage_graphs/bin208_NCGM2.S1.png]

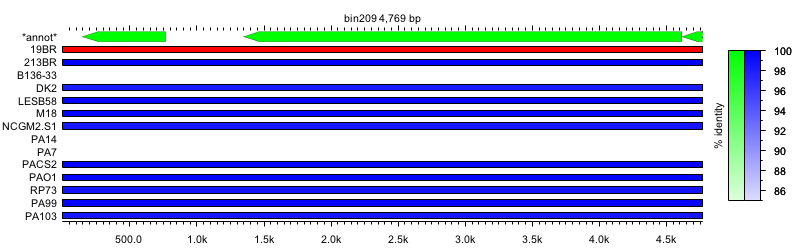

Supplement: Supplementary file 2 — Archive containing output files from ClustAGE analysis of accessory genome sequence files found in Additional file 1. (ZIP 18100 kb) [file 12859_2018_2154_MOESM2_ESM.zip › PA_14genomes_clustage_graphs/bin209_19BR.png]

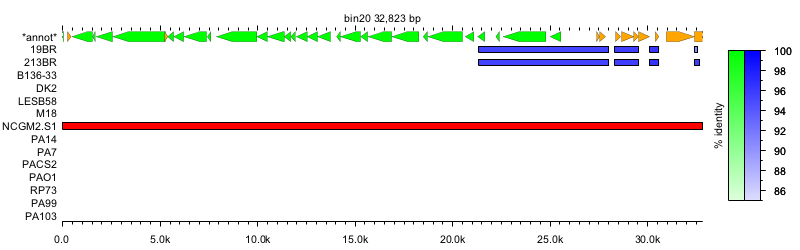

Supplement: Supplementary file 2 — Archive containing output files from ClustAGE analysis of accessory genome sequence files found in Additional file 1. (ZIP 18100 kb) [file 12859_2018_2154_MOESM2_ESM.zip › PA_14genomes_clustage_graphs/bin20_NCGM2.S1.png]

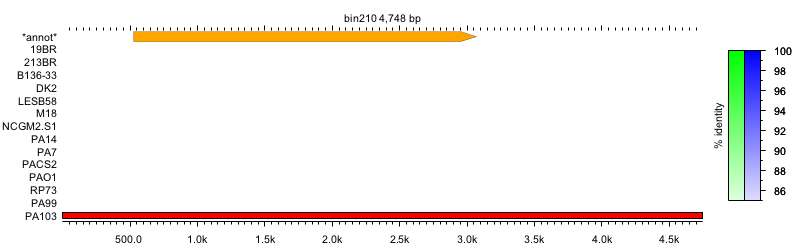

Supplement: Supplementary file 2 — Archive containing output files from ClustAGE analysis of accessory genome sequence files found in Additional file 1. (ZIP 18100 kb) [file 12859_2018_2154_MOESM2_ESM.zip › PA_14genomes_clustage_graphs/bin210_PA103.png]

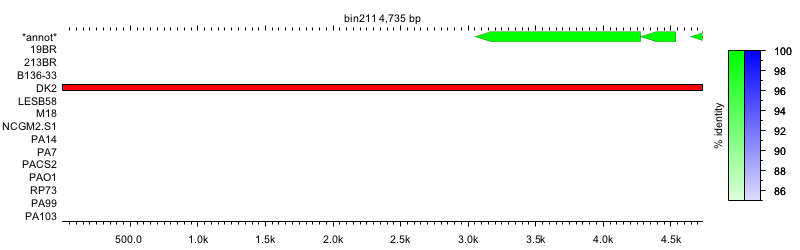

Supplement: Supplementary file 2 — Archive containing output files from ClustAGE analysis of accessory genome sequence files found in Additional file 1. (ZIP 18100 kb) [file 12859_2018_2154_MOESM2_ESM.zip › PA_14genomes_clustage_graphs/bin211_DK2.png]

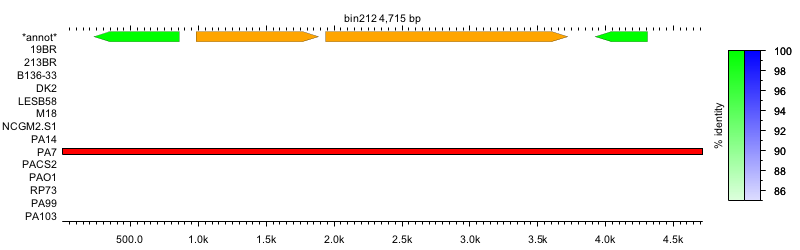

Supplement: Supplementary file 2 — Archive containing output files from ClustAGE analysis of accessory genome sequence files found in Additional file 1. (ZIP 18100 kb) [file 12859_2018_2154_MOESM2_ESM.zip › PA_14genomes_clustage_graphs/bin212_PA7.png]

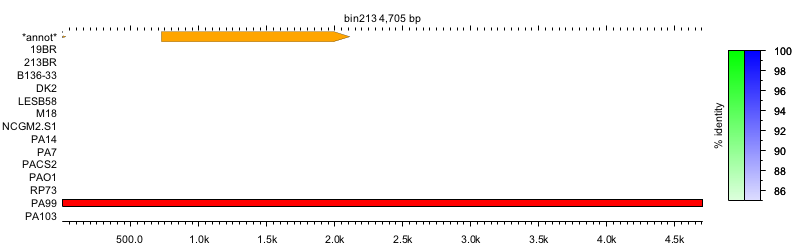

Supplement: Supplementary file 2 — Archive containing output files from ClustAGE analysis of accessory genome sequence files found in Additional file 1. (ZIP 18100 kb) [file 12859_2018_2154_MOESM2_ESM.zip › PA_14genomes_clustage_graphs/bin213_PA99.png]

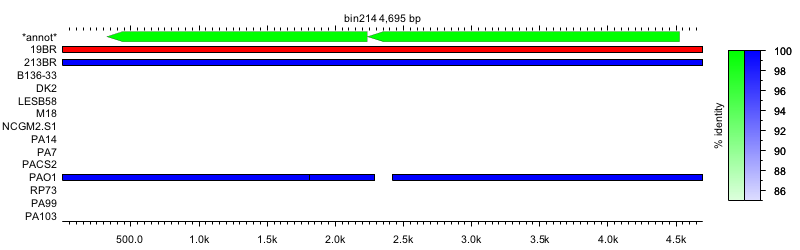

Supplement: Supplementary file 2 — Archive containing output files from ClustAGE analysis of accessory genome sequence files found in Additional file 1. (ZIP 18100 kb) [file 12859_2018_2154_MOESM2_ESM.zip › PA_14genomes_clustage_graphs/bin214_19BR.png]

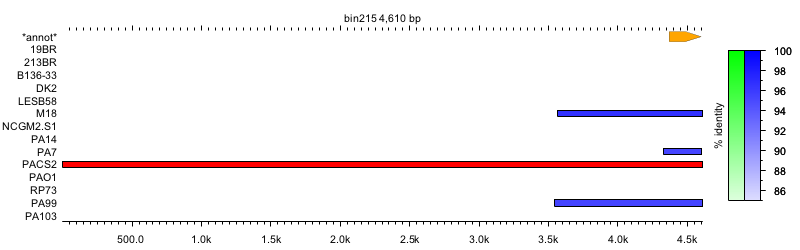

Supplement: Supplementary file 2 — Archive containing output files from ClustAGE analysis of accessory genome sequence files found in Additional file 1. (ZIP 18100 kb) [file 12859_2018_2154_MOESM2_ESM.zip › PA_14genomes_clustage_graphs/bin215_PACS2.png]

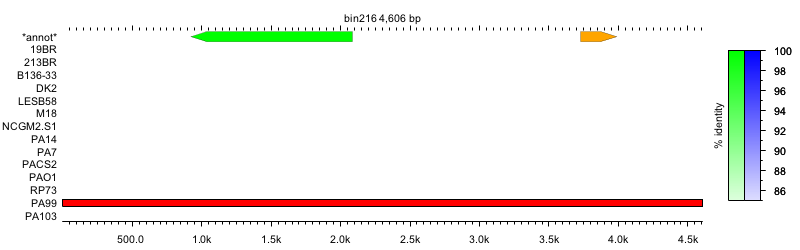

Supplement: Supplementary file 2 — Archive containing output files from ClustAGE analysis of accessory genome sequence files found in Additional file 1. (ZIP 18100 kb) [file 12859_2018_2154_MOESM2_ESM.zip › PA_14genomes_clustage_graphs/bin216_PA99.png]

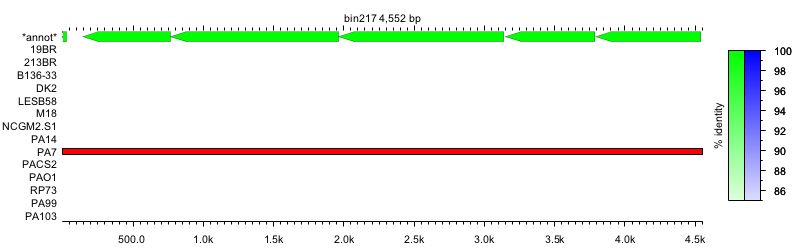

Supplement: Supplementary file 2 — Archive containing output files from ClustAGE analysis of accessory genome sequence files found in Additional file 1. (ZIP 18100 kb) [file 12859_2018_2154_MOESM2_ESM.zip › PA_14genomes_clustage_graphs/bin217_PA7.png]

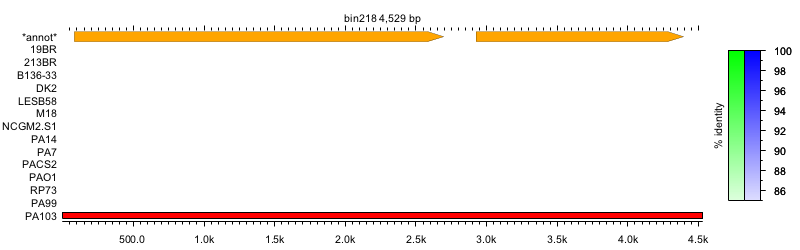

Supplement: Supplementary file 2 — Archive containing output files from ClustAGE analysis of accessory genome sequence files found in Additional file 1. (ZIP 18100 kb) [file 12859_2018_2154_MOESM2_ESM.zip › PA_14genomes_clustage_graphs/bin218_PA103.png]

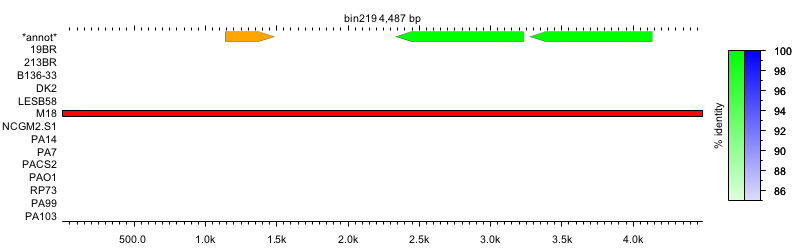

Supplement: Supplementary file 2 — Archive containing output files from ClustAGE analysis of accessory genome sequence files found in Additional file 1. (ZIP 18100 kb) [file 12859_2018_2154_MOESM2_ESM.zip › PA_14genomes_clustage_graphs/bin219_M18.png]

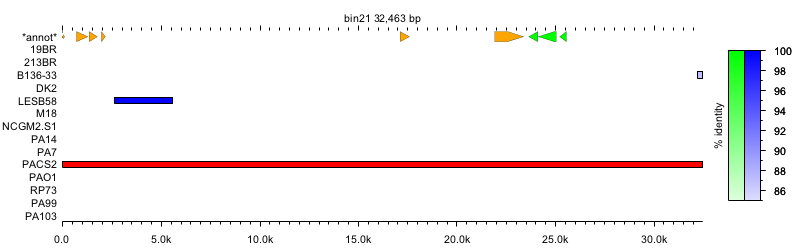

Supplement: Supplementary file 2 — Archive containing output files from ClustAGE analysis of accessory genome sequence files found in Additional file 1. (ZIP 18100 kb) [file 12859_2018_2154_MOESM2_ESM.zip › PA_14genomes_clustage_graphs/bin21_PACS2.png]

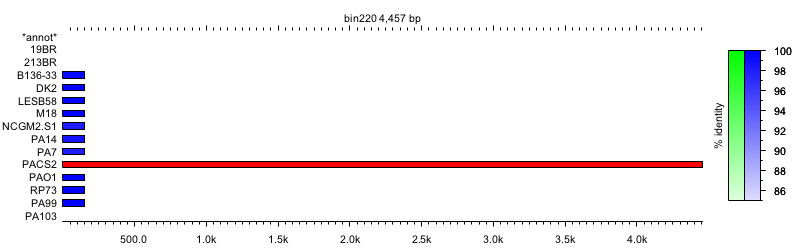

Supplement: Supplementary file 2 — Archive containing output files from ClustAGE analysis of accessory genome sequence files found in Additional file 1. (ZIP 18100 kb) [file 12859_2018_2154_MOESM2_ESM.zip › PA_14genomes_clustage_graphs/bin220_PACS2.png]

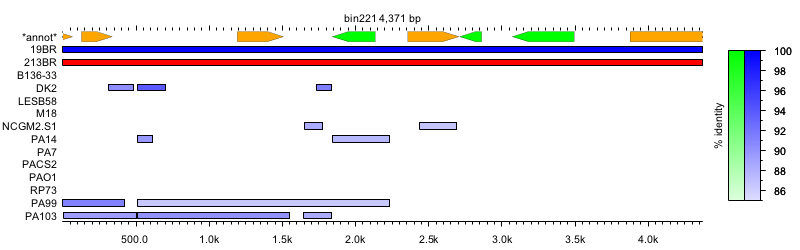

Supplement: Supplementary file 2 — Archive containing output files from ClustAGE analysis of accessory genome sequence files found in Additional file 1. (ZIP 18100 kb) [file 12859_2018_2154_MOESM2_ESM.zip › PA_14genomes_clustage_graphs/bin221_213BR.png]

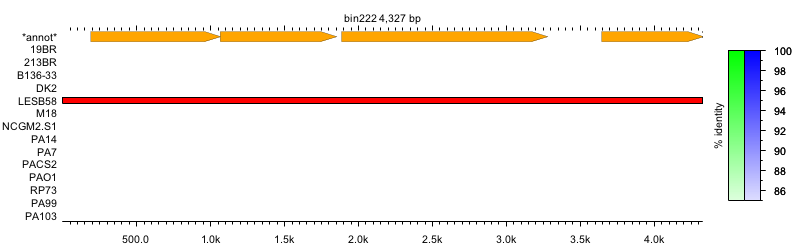

Supplement: Supplementary file 2 — Archive containing output files from ClustAGE analysis of accessory genome sequence files found in Additional file 1. (ZIP 18100 kb) [file 12859_2018_2154_MOESM2_ESM.zip › PA_14genomes_clustage_graphs/bin222_LESB58.png]

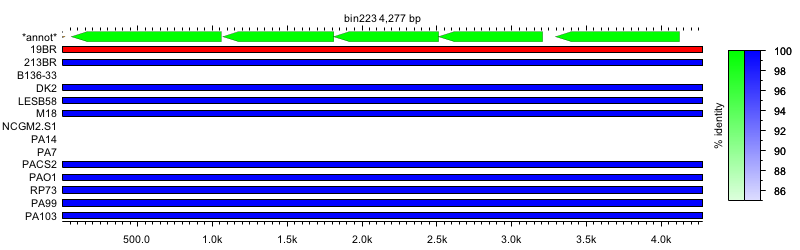

Supplement: Supplementary file 2 — Archive containing output files from ClustAGE analysis of accessory genome sequence files found in Additional file 1. (ZIP 18100 kb) [file 12859_2018_2154_MOESM2_ESM.zip › PA_14genomes_clustage_graphs/bin223_19BR.png]

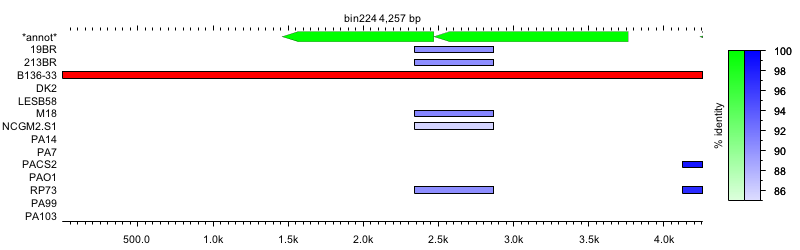

Supplement: Supplementary file 2 — Archive containing output files from ClustAGE analysis of accessory genome sequence files found in Additional file 1. (ZIP 18100 kb) [file 12859_2018_2154_MOESM2_ESM.zip › PA_14genomes_clustage_graphs/bin224_B136-33.png]

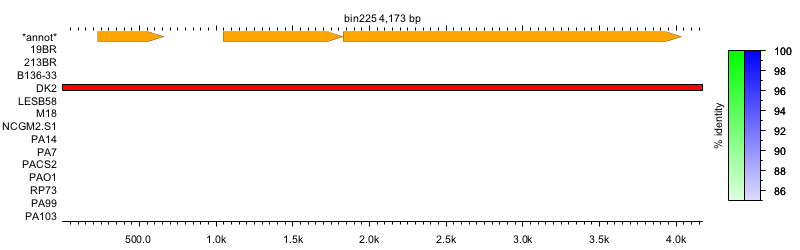

Supplement: Supplementary file 2 — Archive containing output files from ClustAGE analysis of accessory genome sequence files found in Additional file 1. (ZIP 18100 kb) [file 12859_2018_2154_MOESM2_ESM.zip › PA_14genomes_clustage_graphs/bin225_DK2.png]

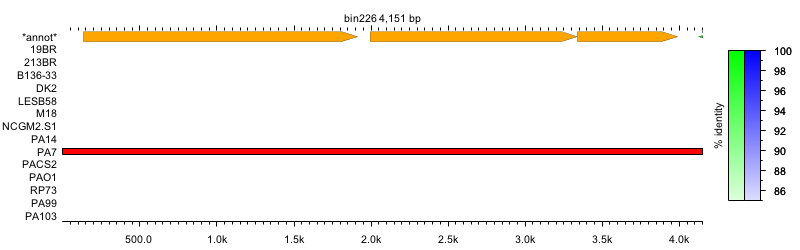

Supplement: Supplementary file 2 — Archive containing output files from ClustAGE analysis of accessory genome sequence files found in Additional file 1. (ZIP 18100 kb) [file 12859_2018_2154_MOESM2_ESM.zip › PA_14genomes_clustage_graphs/bin226_PA7.png]

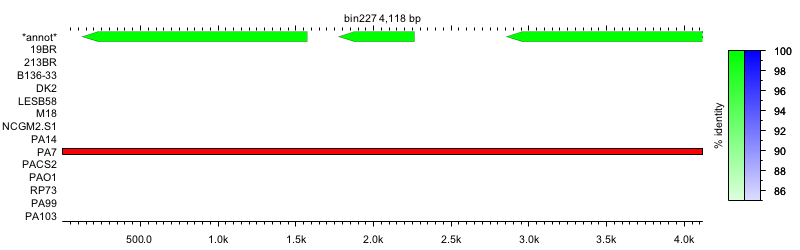

Supplement: Supplementary file 2 — Archive containing output files from ClustAGE analysis of accessory genome sequence files found in Additional file 1. (ZIP 18100 kb) [file 12859_2018_2154_MOESM2_ESM.zip › PA_14genomes_clustage_graphs/bin227_PA7.png]

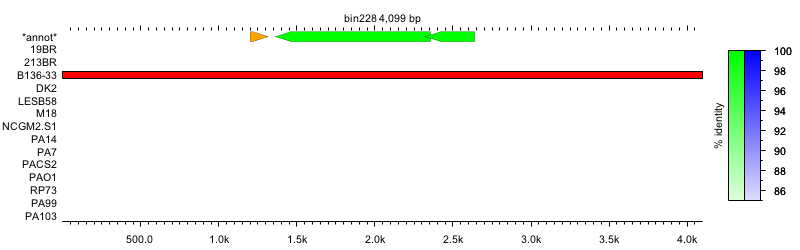

Supplement: Supplementary file 2 — Archive containing output files from ClustAGE analysis of accessory genome sequence files found in Additional file 1. (ZIP 18100 kb) [file 12859_2018_2154_MOESM2_ESM.zip › PA_14genomes_clustage_graphs/bin228_B136-33.png]

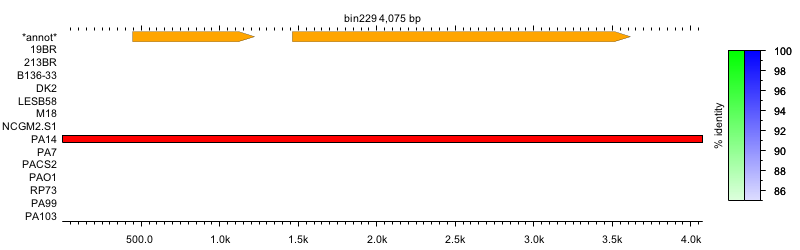

Supplement: Supplementary file 2 — Archive containing output files from ClustAGE analysis of accessory genome sequence files found in Additional file 1. (ZIP 18100 kb) [file 12859_2018_2154_MOESM2_ESM.zip › PA_14genomes_clustage_graphs/bin229_PA14.png]

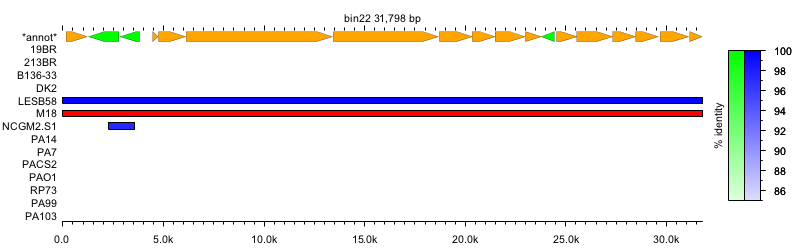

Supplement: Supplementary file 2 — Archive containing output files from ClustAGE analysis of accessory genome sequence files found in Additional file 1. (ZIP 18100 kb) [file 12859_2018_2154_MOESM2_ESM.zip › PA_14genomes_clustage_graphs/bin22_M18.png]

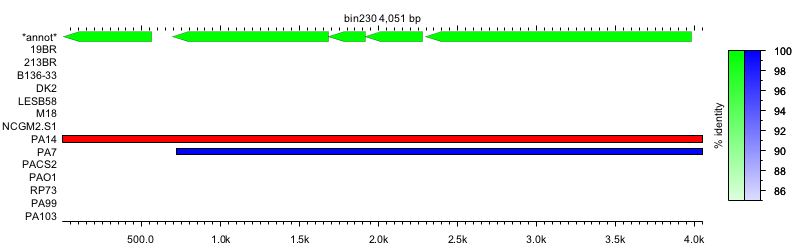

Supplement: Supplementary file 2 — Archive containing output files from ClustAGE analysis of accessory genome sequence files found in Additional file 1. (ZIP 18100 kb) [file 12859_2018_2154_MOESM2_ESM.zip › PA_14genomes_clustage_graphs/bin230_PA14.png]

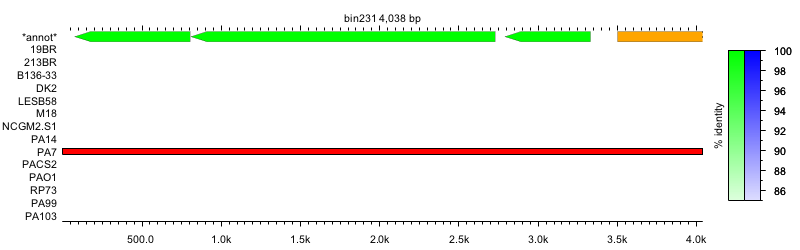

Supplement: Supplementary file 2 — Archive containing output files from ClustAGE analysis of accessory genome sequence files found in Additional file 1. (ZIP 18100 kb) [file 12859_2018_2154_MOESM2_ESM.zip › PA_14genomes_clustage_graphs/bin231_PA7.png]

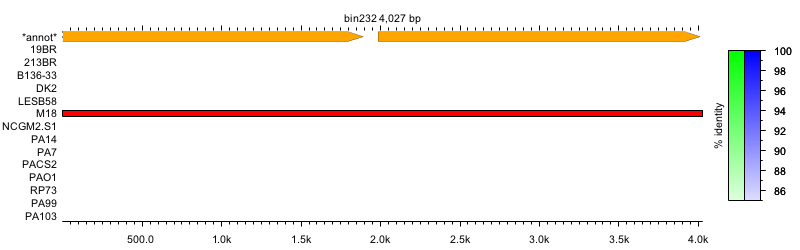

Supplement: Supplementary file 2 — Archive containing output files from ClustAGE analysis of accessory genome sequence files found in Additional file 1. (ZIP 18100 kb) [file 12859_2018_2154_MOESM2_ESM.zip › PA_14genomes_clustage_graphs/bin232_M18.png]

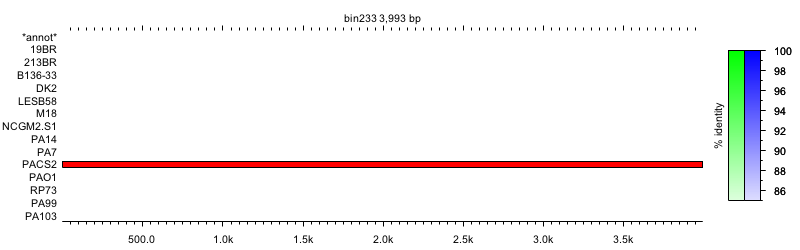

Supplement: Supplementary file 2 — Archive containing output files from ClustAGE analysis of accessory genome sequence files found in Additional file 1. (ZIP 18100 kb) [file 12859_2018_2154_MOESM2_ESM.zip › PA_14genomes_clustage_graphs/bin233_PACS2.png]

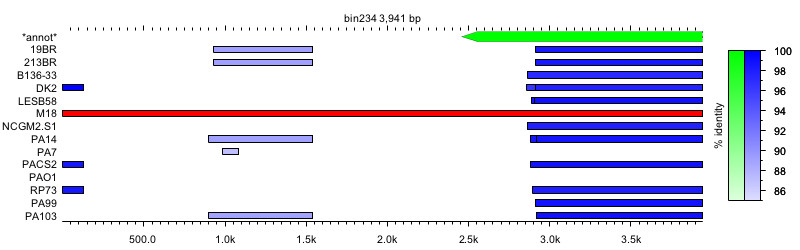

Supplement: Supplementary file 2 — Archive containing output files from ClustAGE analysis of accessory genome sequence files found in Additional file 1. (ZIP 18100 kb) [file 12859_2018_2154_MOESM2_ESM.zip › PA_14genomes_clustage_graphs/bin234_M18.png]

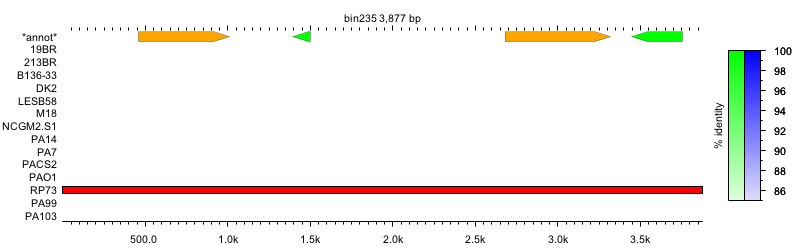

Supplement: Supplementary file 2 — Archive containing output files from ClustAGE analysis of accessory genome sequence files found in Additional file 1. (ZIP 18100 kb) [file 12859_2018_2154_MOESM2_ESM.zip › PA_14genomes_clustage_graphs/bin235_RP73.png]

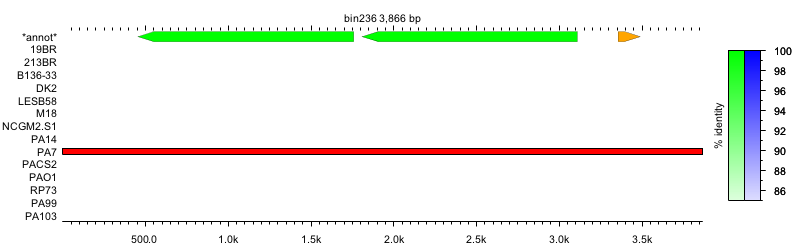

Supplement: Supplementary file 2 — Archive containing output files from ClustAGE analysis of accessory genome sequence files found in Additional file 1. (ZIP 18100 kb) [file 12859_2018_2154_MOESM2_ESM.zip › PA_14genomes_clustage_graphs/bin236_PA7.png]

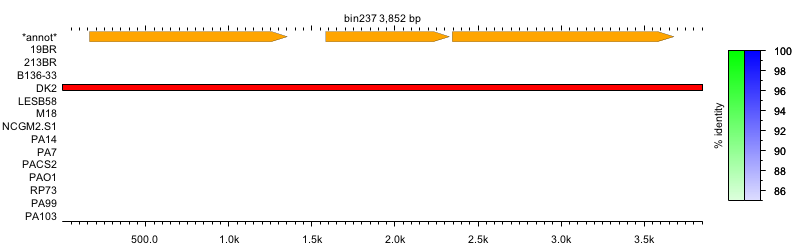

Supplement: Supplementary file 2 — Archive containing output files from ClustAGE analysis of accessory genome sequence files found in Additional file 1. (ZIP 18100 kb) [file 12859_2018_2154_MOESM2_ESM.zip › PA_14genomes_clustage_graphs/bin237_DK2.png]

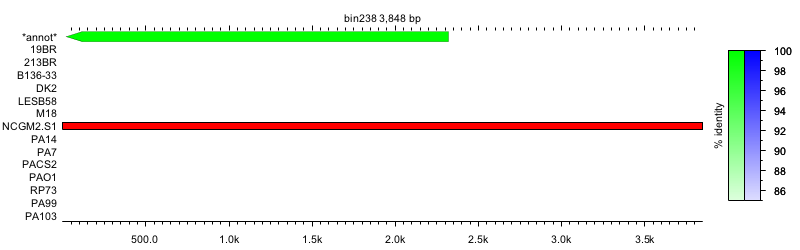

Supplement: Supplementary file 2 — Archive containing output files from ClustAGE analysis of accessory genome sequence files found in Additional file 1. (ZIP 18100 kb) [file 12859_2018_2154_MOESM2_ESM.zip › PA_14genomes_clustage_graphs/bin238_NCGM2.S1.png]

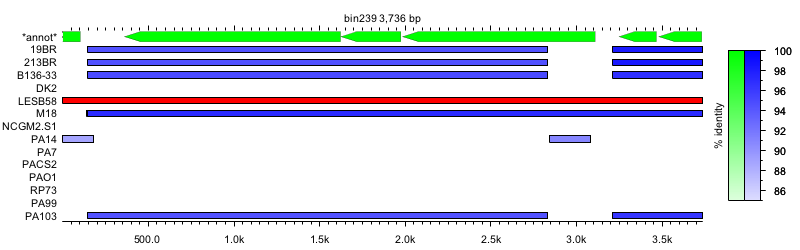

Supplement: Supplementary file 2 — Archive containing output files from ClustAGE analysis of accessory genome sequence files found in Additional file 1. (ZIP 18100 kb) [file 12859_2018_2154_MOESM2_ESM.zip › PA_14genomes_clustage_graphs/bin239_LESB58.png]

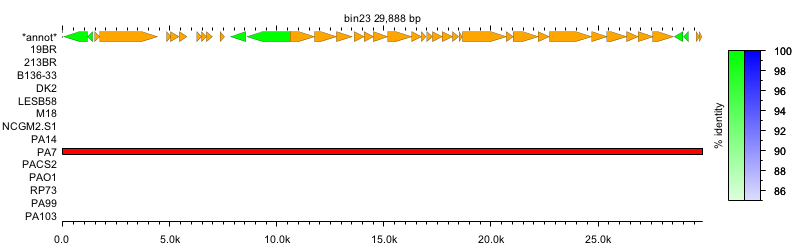

Supplement: Supplementary file 2 — Archive containing output files from ClustAGE analysis of accessory genome sequence files found in Additional file 1. (ZIP 18100 kb) [file 12859_2018_2154_MOESM2_ESM.zip › PA_14genomes_clustage_graphs/bin23_PA7.png]

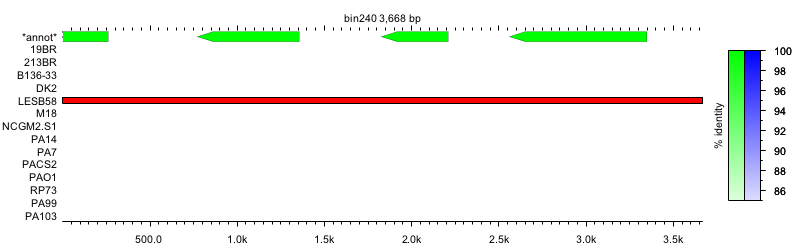

Supplement: Supplementary file 2 — Archive containing output files from ClustAGE analysis of accessory genome sequence files found in Additional file 1. (ZIP 18100 kb) [file 12859_2018_2154_MOESM2_ESM.zip › PA_14genomes_clustage_graphs/bin240_LESB58.png]

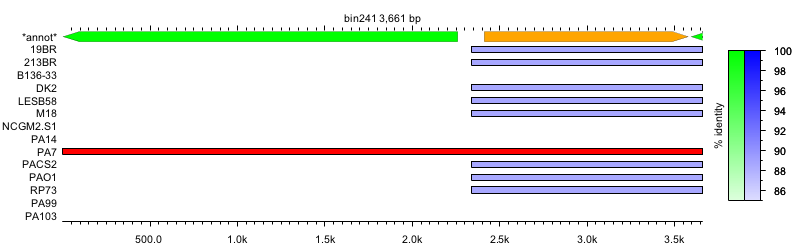

Supplement: Supplementary file 2 — Archive containing output files from ClustAGE analysis of accessory genome sequence files found in Additional file 1. (ZIP 18100 kb) [file 12859_2018_2154_MOESM2_ESM.zip › PA_14genomes_clustage_graphs/bin241_PA7.png]

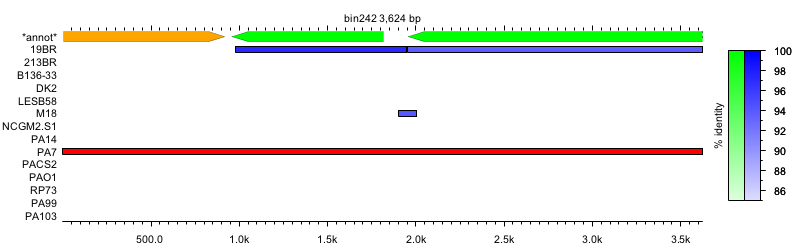

Supplement: Supplementary file 2 — Archive containing output files from ClustAGE analysis of accessory genome sequence files found in Additional file 1. (ZIP 18100 kb) [file 12859_2018_2154_MOESM2_ESM.zip › PA_14genomes_clustage_graphs/bin242_PA7.png]

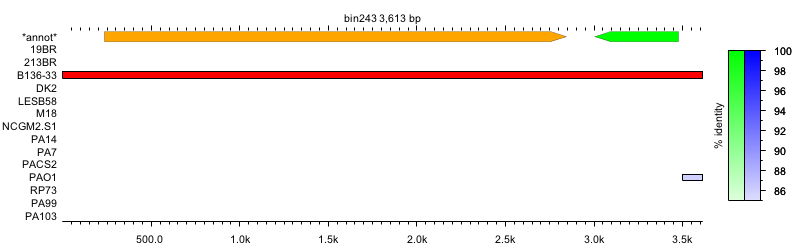

Supplement: Supplementary file 2 — Archive containing output files from ClustAGE analysis of accessory genome sequence files found in Additional file 1. (ZIP 18100 kb) [file 12859_2018_2154_MOESM2_ESM.zip › PA_14genomes_clustage_graphs/bin243_B136-33.png]

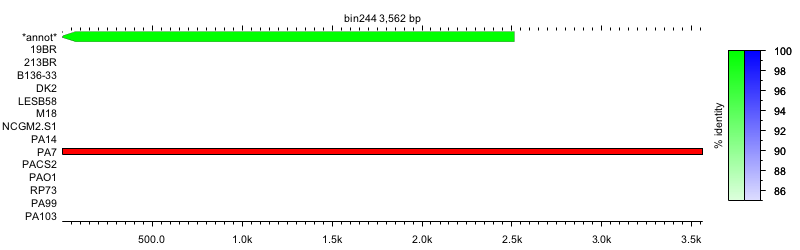

Supplement: Supplementary file 2 — Archive containing output files from ClustAGE analysis of accessory genome sequence files found in Additional file 1. (ZIP 18100 kb) [file 12859_2018_2154_MOESM2_ESM.zip › PA_14genomes_clustage_graphs/bin244_PA7.png]

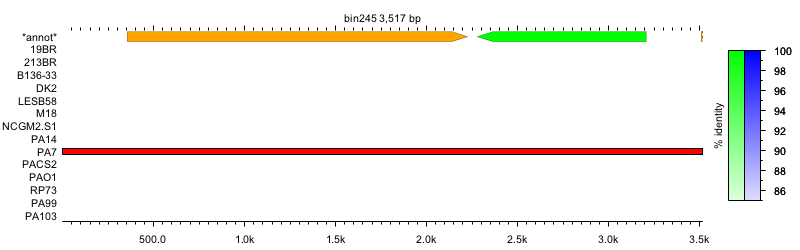

Supplement: Supplementary file 2 — Archive containing output files from ClustAGE analysis of accessory genome sequence files found in Additional file 1. (ZIP 18100 kb) [file 12859_2018_2154_MOESM2_ESM.zip › PA_14genomes_clustage_graphs/bin245_PA7.png]

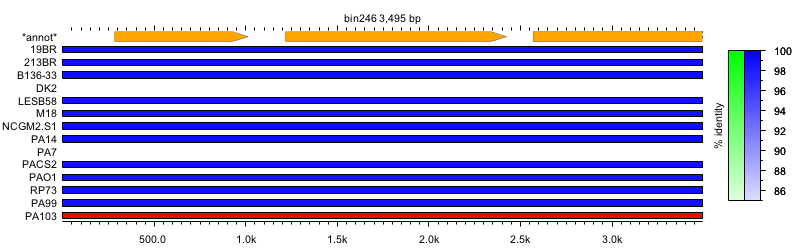

Supplement: Supplementary file 2 — Archive containing output files from ClustAGE analysis of accessory genome sequence files found in Additional file 1. (ZIP 18100 kb) [file 12859_2018_2154_MOESM2_ESM.zip › PA_14genomes_clustage_graphs/bin246_PA103.png]

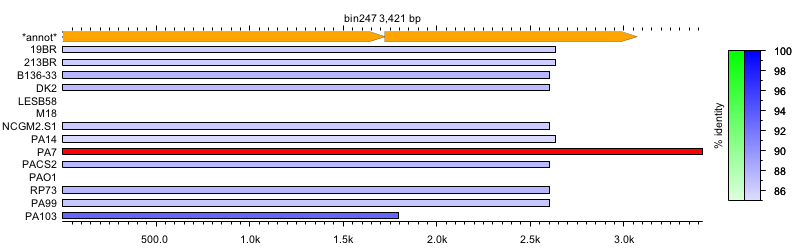

Supplement: Supplementary file 2 — Archive containing output files from ClustAGE analysis of accessory genome sequence files found in Additional file 1. (ZIP 18100 kb) [file 12859_2018_2154_MOESM2_ESM.zip › PA_14genomes_clustage_graphs/bin247_PA7.png]

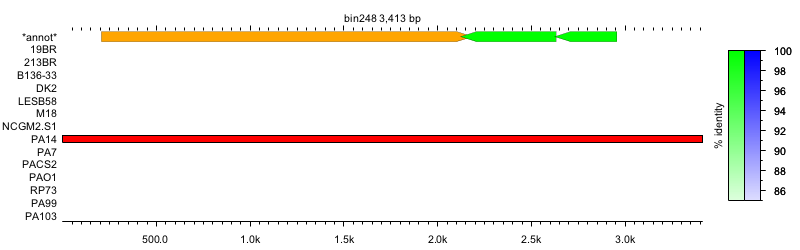

Supplement: Supplementary file 2 — Archive containing output files from ClustAGE analysis of accessory genome sequence files found in Additional file 1. (ZIP 18100 kb) [file 12859_2018_2154_MOESM2_ESM.zip › PA_14genomes_clustage_graphs/bin248_PA14.png]

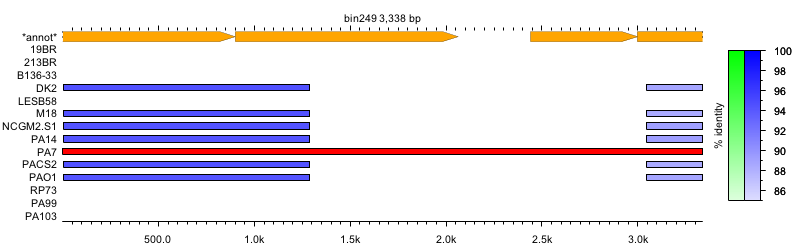

Supplement: Supplementary file 2 — Archive containing output files from ClustAGE analysis of accessory genome sequence files found in Additional file 1. (ZIP 18100 kb) [file 12859_2018_2154_MOESM2_ESM.zip › PA_14genomes_clustage_graphs/bin249_PA7.png]

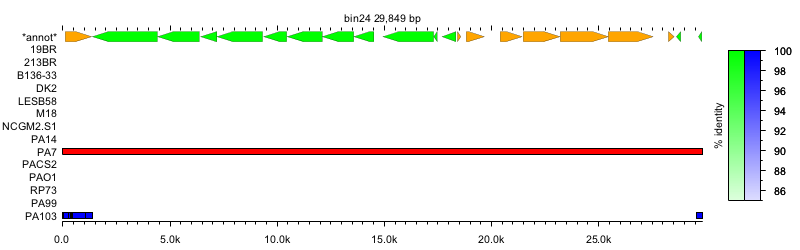

Supplement: Supplementary file 2 — Archive containing output files from ClustAGE analysis of accessory genome sequence files found in Additional file 1. (ZIP 18100 kb) [file 12859_2018_2154_MOESM2_ESM.zip › PA_14genomes_clustage_graphs/bin24_PA7.png]

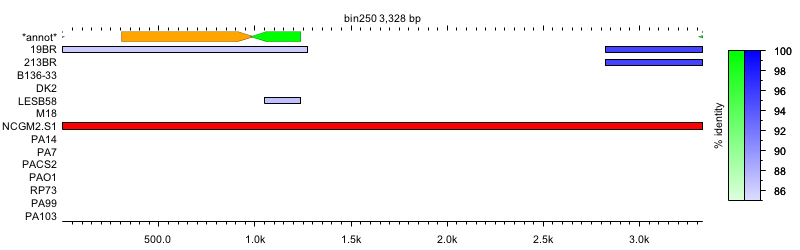

Supplement: Supplementary file 2 — Archive containing output files from ClustAGE analysis of accessory genome sequence files found in Additional file 1. (ZIP 18100 kb) [file 12859_2018_2154_MOESM2_ESM.zip › PA_14genomes_clustage_graphs/bin250_NCGM2.S1.png]

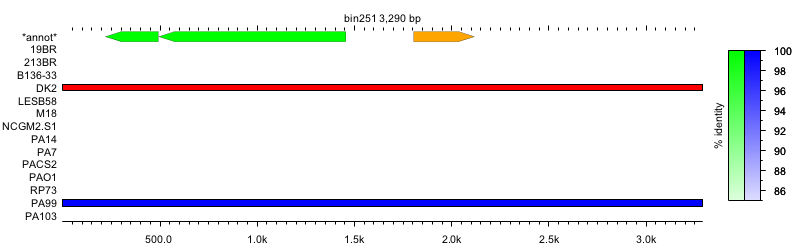

Supplement: Supplementary file 2 — Archive containing output files from ClustAGE analysis of accessory genome sequence files found in Additional file 1. (ZIP 18100 kb) [file 12859_2018_2154_MOESM2_ESM.zip › PA_14genomes_clustage_graphs/bin251_DK2.png]

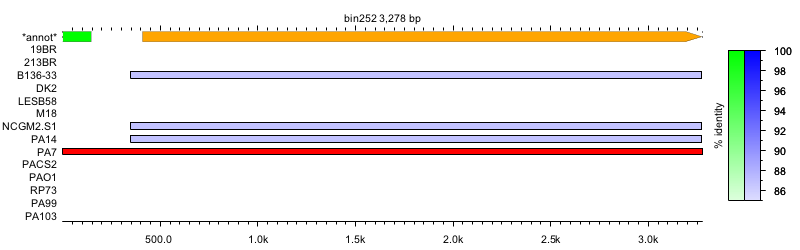

Supplement: Supplementary file 2 — Archive containing output files from ClustAGE analysis of accessory genome sequence files found in Additional file 1. (ZIP 18100 kb) [file 12859_2018_2154_MOESM2_ESM.zip › PA_14genomes_clustage_graphs/bin252_PA7.png]

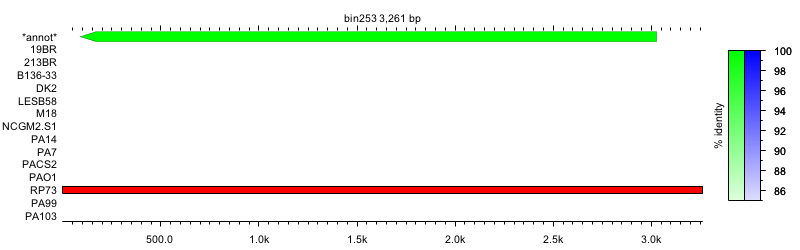

Supplement: Supplementary file 2 — Archive containing output files from ClustAGE analysis of accessory genome sequence files found in Additional file 1. (ZIP 18100 kb) [file 12859_2018_2154_MOESM2_ESM.zip › PA_14genomes_clustage_graphs/bin253_RP73.png]

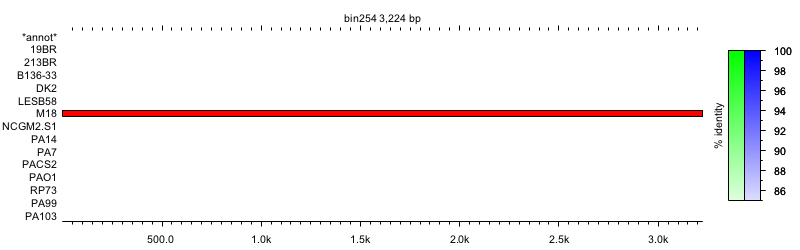

Supplement: Supplementary file 2 — Archive containing output files from ClustAGE analysis of accessory genome sequence files found in Additional file 1. (ZIP 18100 kb) [file 12859_2018_2154_MOESM2_ESM.zip › PA_14genomes_clustage_graphs/bin254_M18.png]

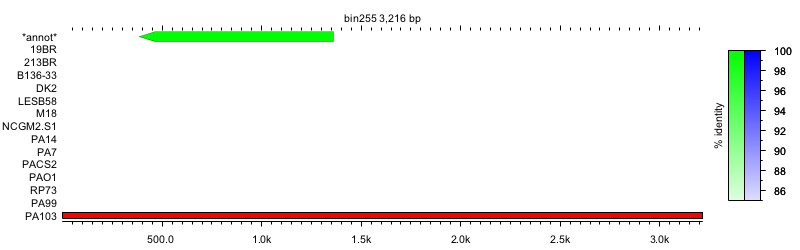

Supplement: Supplementary file 2 — Archive containing output files from ClustAGE analysis of accessory genome sequence files found in Additional file 1. (ZIP 18100 kb) [file 12859_2018_2154_MOESM2_ESM.zip › PA_14genomes_clustage_graphs/bin255_PA103.png]

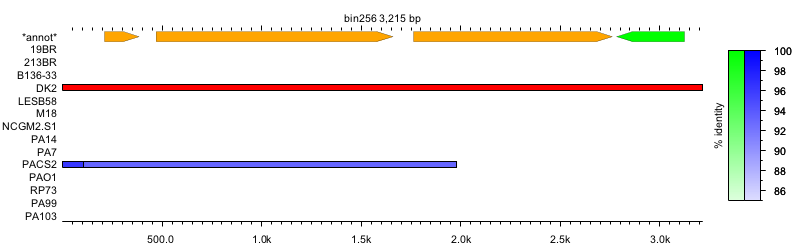

Supplement: Supplementary file 2 — Archive containing output files from ClustAGE analysis of accessory genome sequence files found in Additional file 1. (ZIP 18100 kb) [file 12859_2018_2154_MOESM2_ESM.zip › PA_14genomes_clustage_graphs/bin256_DK2.png]

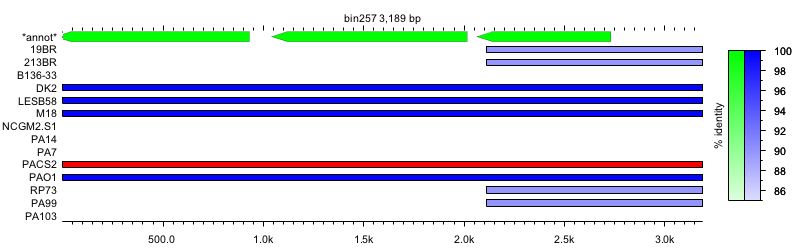

Supplement: Supplementary file 2 — Archive containing output files from ClustAGE analysis of accessory genome sequence files found in Additional file 1. (ZIP 18100 kb) [file 12859_2018_2154_MOESM2_ESM.zip › PA_14genomes_clustage_graphs/bin257_PACS2.png]

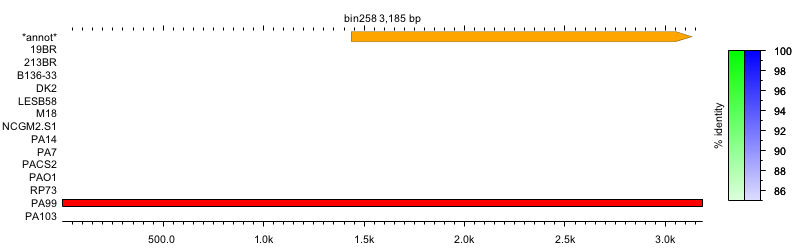

Supplement: Supplementary file 2 — Archive containing output files from ClustAGE analysis of accessory genome sequence files found in Additional file 1. (ZIP 18100 kb) [file 12859_2018_2154_MOESM2_ESM.zip › PA_14genomes_clustage_graphs/bin258_PA99.png]

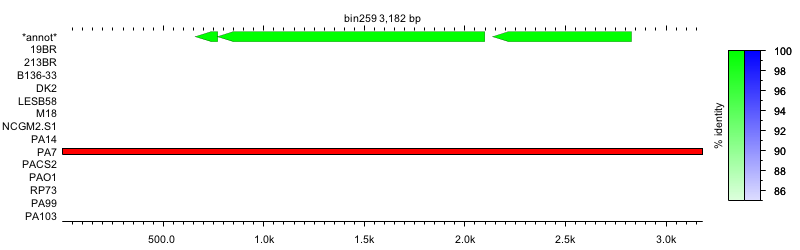

Supplement: Supplementary file 2 — Archive containing output files from ClustAGE analysis of accessory genome sequence files found in Additional file 1. (ZIP 18100 kb) [file 12859_2018_2154_MOESM2_ESM.zip › PA_14genomes_clustage_graphs/bin259_PA7.png]

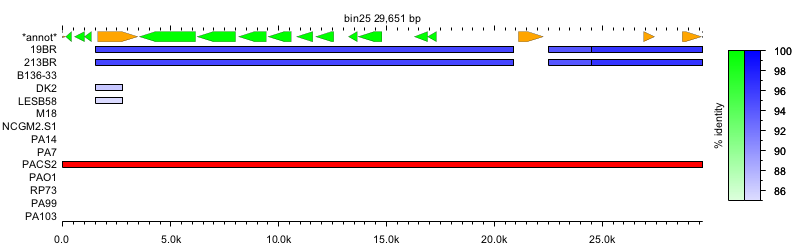

Supplement: Supplementary file 2 — Archive containing output files from ClustAGE analysis of accessory genome sequence files found in Additional file 1. (ZIP 18100 kb) [file 12859_2018_2154_MOESM2_ESM.zip › PA_14genomes_clustage_graphs/bin25_PACS2.png]

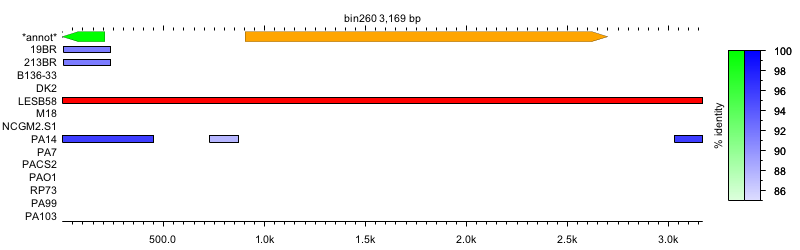

Supplement: Supplementary file 2 — Archive containing output files from ClustAGE analysis of accessory genome sequence files found in Additional file 1. (ZIP 18100 kb) [file 12859_2018_2154_MOESM2_ESM.zip › PA_14genomes_clustage_graphs/bin260_LESB58.png]

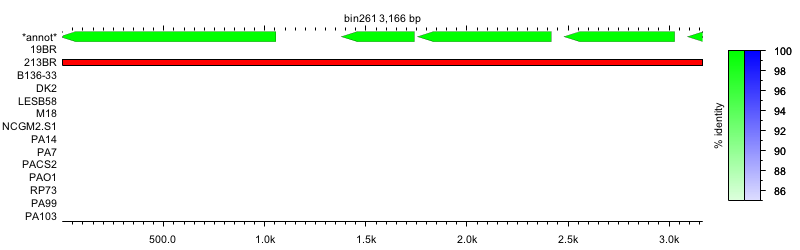

Supplement: Supplementary file 2 — Archive containing output files from ClustAGE analysis of accessory genome sequence files found in Additional file 1. (ZIP 18100 kb) [file 12859_2018_2154_MOESM2_ESM.zip › PA_14genomes_clustage_graphs/bin261_213BR.png]

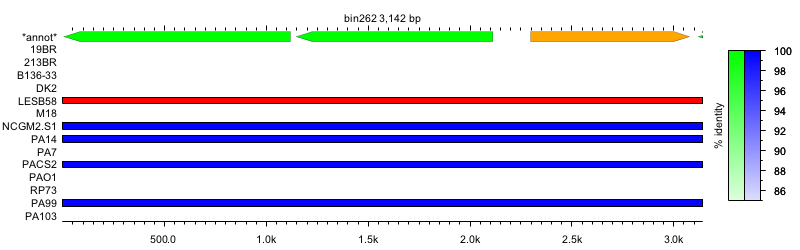

Supplement: Supplementary file 2 — Archive containing output files from ClustAGE analysis of accessory genome sequence files found in Additional file 1. (ZIP 18100 kb) [file 12859_2018_2154_MOESM2_ESM.zip › PA_14genomes_clustage_graphs/bin262_LESB58.png]

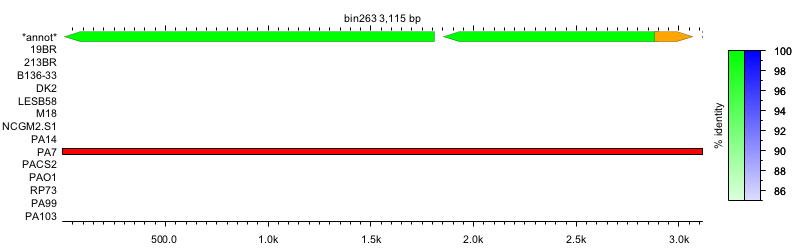

Supplement: Supplementary file 2 — Archive containing output files from ClustAGE analysis of accessory genome sequence files found in Additional file 1. (ZIP 18100 kb) [file 12859_2018_2154_MOESM2_ESM.zip › PA_14genomes_clustage_graphs/bin263_PA7.png]

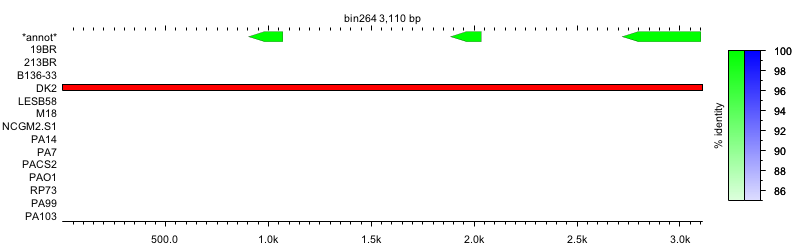

Supplement: Supplementary file 2 — Archive containing output files from ClustAGE analysis of accessory genome sequence files found in Additional file 1. (ZIP 18100 kb) [file 12859_2018_2154_MOESM2_ESM.zip › PA_14genomes_clustage_graphs/bin264_DK2.png]

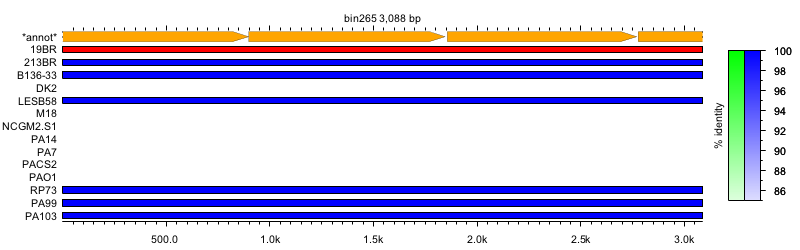

Supplement: Supplementary file 2 — Archive containing output files from ClustAGE analysis of accessory genome sequence files found in Additional file 1. (ZIP 18100 kb) [file 12859_2018_2154_MOESM2_ESM.zip › PA_14genomes_clustage_graphs/bin265_19BR.png]

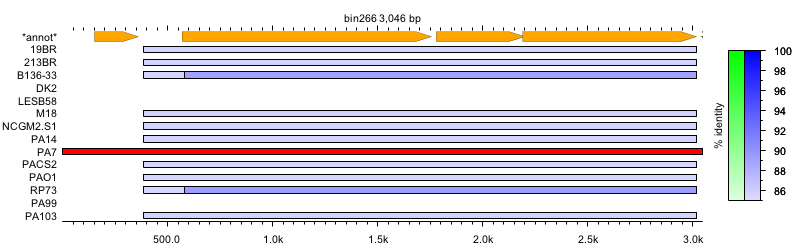

Supplement: Supplementary file 2 — Archive containing output files from ClustAGE analysis of accessory genome sequence files found in Additional file 1. (ZIP 18100 kb) [file 12859_2018_2154_MOESM2_ESM.zip › PA_14genomes_clustage_graphs/bin266_PA7.png]

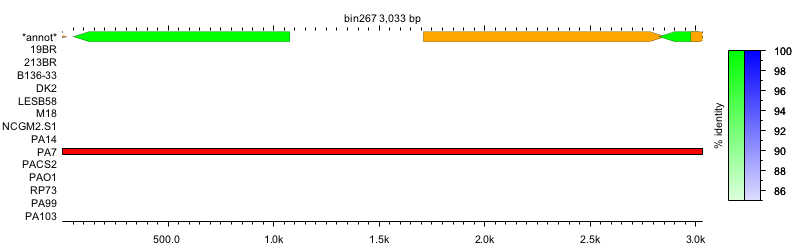

Supplement: Supplementary file 2 — Archive containing output files from ClustAGE analysis of accessory genome sequence files found in Additional file 1. (ZIP 18100 kb) [file 12859_2018_2154_MOESM2_ESM.zip › PA_14genomes_clustage_graphs/bin267_PA7.png]

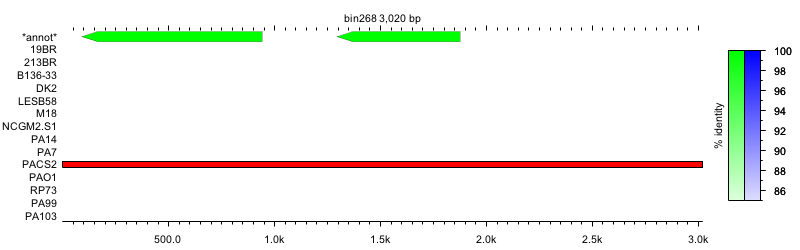

Supplement: Supplementary file 2 — Archive containing output files from ClustAGE analysis of accessory genome sequence files found in Additional file 1. (ZIP 18100 kb) [file 12859_2018_2154_MOESM2_ESM.zip › PA_14genomes_clustage_graphs/bin268_PACS2.png]

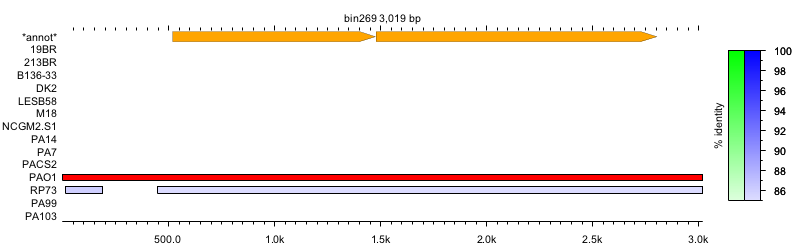

Supplement: Supplementary file 2 — Archive containing output files from ClustAGE analysis of accessory genome sequence files found in Additional file 1. (ZIP 18100 kb) [file 12859_2018_2154_MOESM2_ESM.zip › PA_14genomes_clustage_graphs/bin269_PAO1.png]

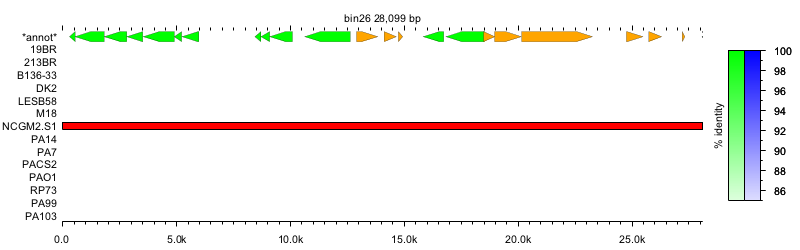

Supplement: Supplementary file 2 — Archive containing output files from ClustAGE analysis of accessory genome sequence files found in Additional file 1. (ZIP 18100 kb) [file 12859_2018_2154_MOESM2_ESM.zip › PA_14genomes_clustage_graphs/bin26_NCGM2.S1.png]

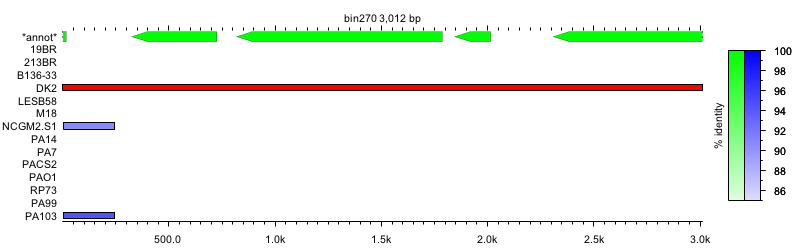

Supplement: Supplementary file 2 — Archive containing output files from ClustAGE analysis of accessory genome sequence files found in Additional file 1. (ZIP 18100 kb) [file 12859_2018_2154_MOESM2_ESM.zip › PA_14genomes_clustage_graphs/bin270_DK2.png]

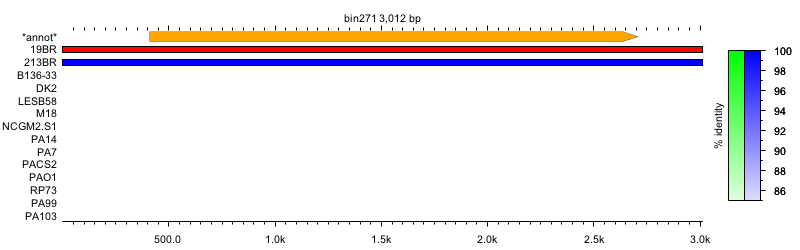

Supplement: Supplementary file 2 — Archive containing output files from ClustAGE analysis of accessory genome sequence files found in Additional file 1. (ZIP 18100 kb) [file 12859_2018_2154_MOESM2_ESM.zip › PA_14genomes_clustage_graphs/bin271_19BR.png]

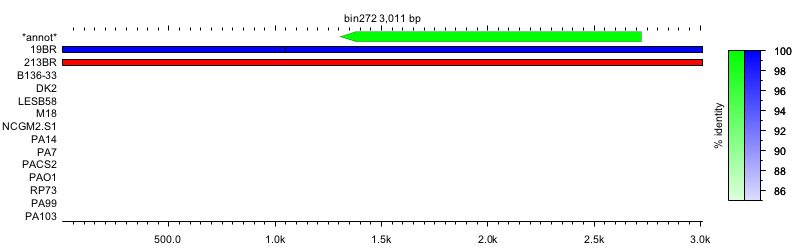

Supplement: Supplementary file 2 — Archive containing output files from ClustAGE analysis of accessory genome sequence files found in Additional file 1. (ZIP 18100 kb) [file 12859_2018_2154_MOESM2_ESM.zip › PA_14genomes_clustage_graphs/bin272_213BR.png]

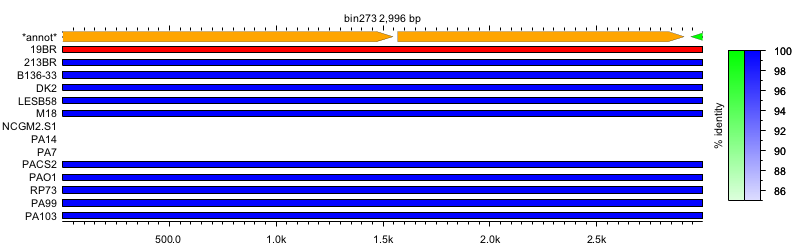

Supplement: Supplementary file 2 — Archive containing output files from ClustAGE analysis of accessory genome sequence files found in Additional file 1. (ZIP 18100 kb) [file 12859_2018_2154_MOESM2_ESM.zip › PA_14genomes_clustage_graphs/bin273_19BR.png]

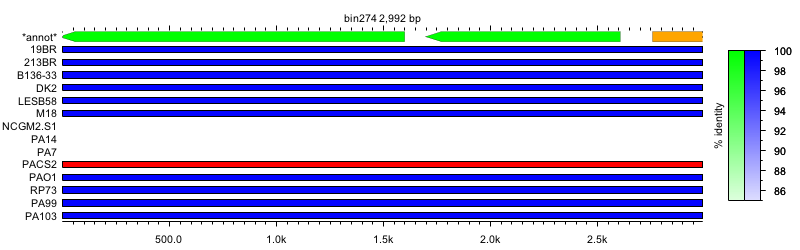

Supplement: Supplementary file 2 — Archive containing output files from ClustAGE analysis of accessory genome sequence files found in Additional file 1. (ZIP 18100 kb) [file 12859_2018_2154_MOESM2_ESM.zip › PA_14genomes_clustage_graphs/bin274_PACS2.png]

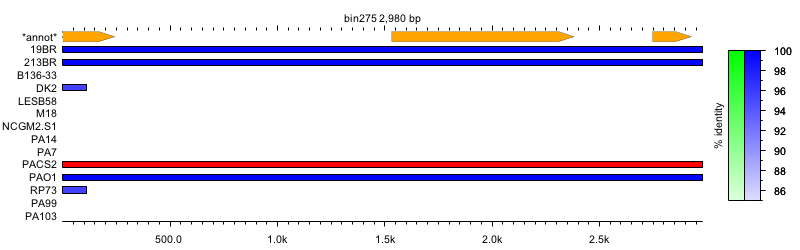

Supplement: Supplementary file 2 — Archive containing output files from ClustAGE analysis of accessory genome sequence files found in Additional file 1. (ZIP 18100 kb) [file 12859_2018_2154_MOESM2_ESM.zip › PA_14genomes_clustage_graphs/bin275_PACS2.png]

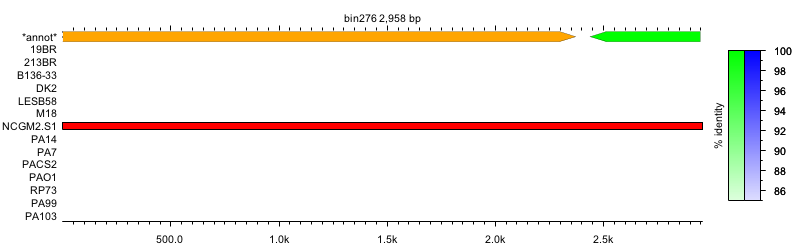

Supplement: Supplementary file 2 — Archive containing output files from ClustAGE analysis of accessory genome sequence files found in Additional file 1. (ZIP 18100 kb) [file 12859_2018_2154_MOESM2_ESM.zip › PA_14genomes_clustage_graphs/bin276_NCGM2.S1.png]

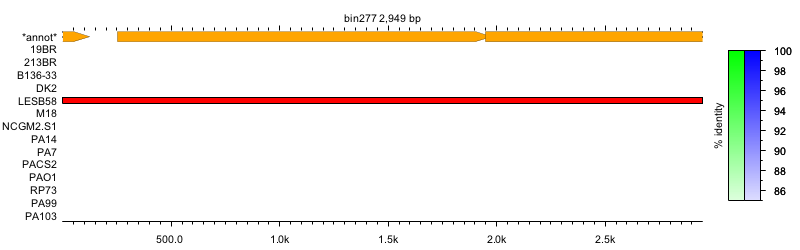

Supplement: Supplementary file 2 — Archive containing output files from ClustAGE analysis of accessory genome sequence files found in Additional file 1. (ZIP 18100 kb) [file 12859_2018_2154_MOESM2_ESM.zip › PA_14genomes_clustage_graphs/bin277_LESB58.png]
