# Supplementary figures and images for: ClustAGE: a tool for clustering and distribution analysis of bacterial accessory genomic elements (part 1 of 2)
Source: BMC Bioinformatics. 2018 Apr 20;19:150. doi: 10.1186/s12859-018-2154-x (PMC5910555; doi:10.1186/s12859-018-2154-x)

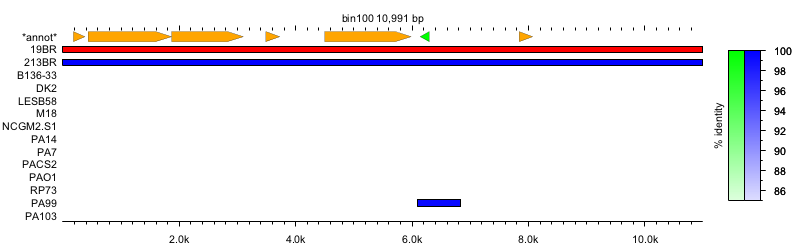

Supplement: Supplementary file 2 — Archive containing output files from ClustAGE analysis of accessory genome sequence files found in Additional file 1. (ZIP 18100 kb) [file 12859_2018_2154_MOESM2_ESM.zip › PA_14genomes_clustage_graphs/bin100_19BR.png]

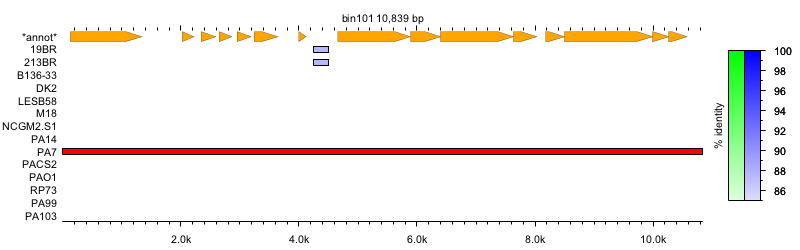

Supplement: Supplementary file 2 — Archive containing output files from ClustAGE analysis of accessory genome sequence files found in Additional file 1. (ZIP 18100 kb) [file 12859_2018_2154_MOESM2_ESM.zip › PA_14genomes_clustage_graphs/bin101_PA7.png]

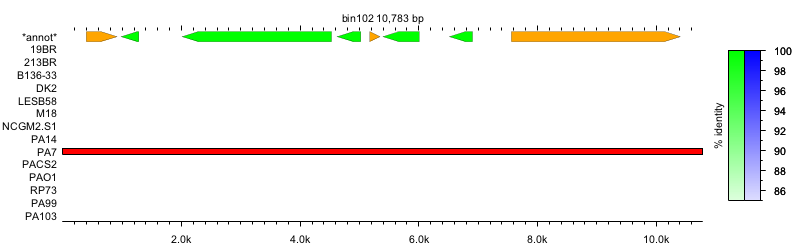

Supplement: Supplementary file 2 — Archive containing output files from ClustAGE analysis of accessory genome sequence files found in Additional file 1. (ZIP 18100 kb) [file 12859_2018_2154_MOESM2_ESM.zip › PA_14genomes_clustage_graphs/bin102_PA7.png]

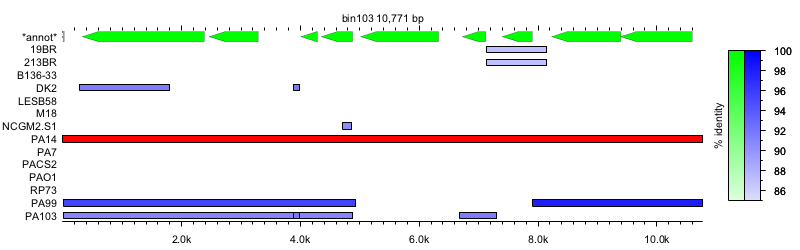

Supplement: Supplementary file 2 — Archive containing output files from ClustAGE analysis of accessory genome sequence files found in Additional file 1. (ZIP 18100 kb) [file 12859_2018_2154_MOESM2_ESM.zip › PA_14genomes_clustage_graphs/bin103_PA14.png]

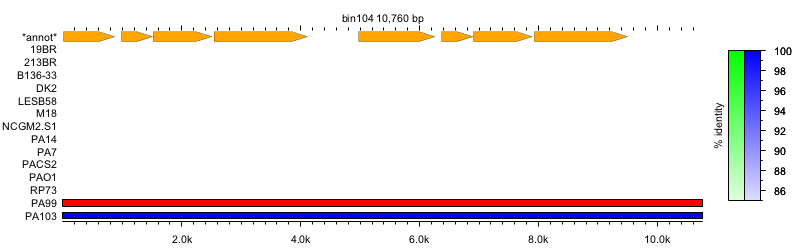

Supplement: Supplementary file 2 — Archive containing output files from ClustAGE analysis of accessory genome sequence files found in Additional file 1. (ZIP 18100 kb) [file 12859_2018_2154_MOESM2_ESM.zip › PA_14genomes_clustage_graphs/bin104_PA99.png]

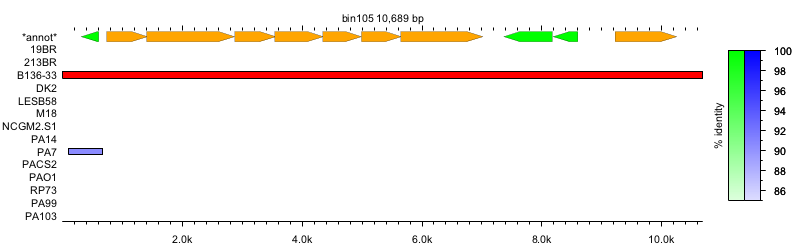

Supplement: Supplementary file 2 — Archive containing output files from ClustAGE analysis of accessory genome sequence files found in Additional file 1. (ZIP 18100 kb) [file 12859_2018_2154_MOESM2_ESM.zip › PA_14genomes_clustage_graphs/bin105_B136-33.png]

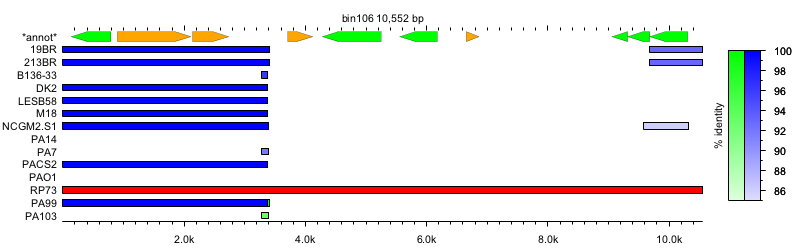

Supplement: Supplementary file 2 — Archive containing output files from ClustAGE analysis of accessory genome sequence files found in Additional file 1. (ZIP 18100 kb) [file 12859_2018_2154_MOESM2_ESM.zip › PA_14genomes_clustage_graphs/bin106_RP73.png]

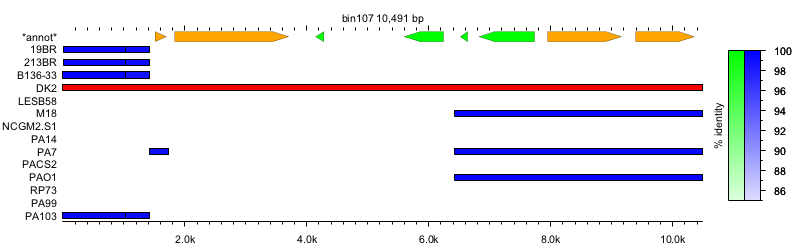

Supplement: Supplementary file 2 — Archive containing output files from ClustAGE analysis of accessory genome sequence files found in Additional file 1. (ZIP 18100 kb) [file 12859_2018_2154_MOESM2_ESM.zip › PA_14genomes_clustage_graphs/bin107_DK2.png]

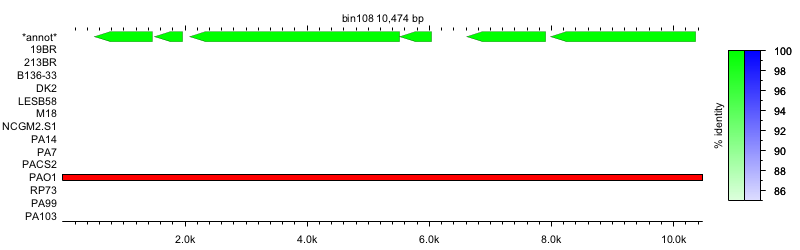

Supplement: Supplementary file 2 — Archive containing output files from ClustAGE analysis of accessory genome sequence files found in Additional file 1. (ZIP 18100 kb) [file 12859_2018_2154_MOESM2_ESM.zip › PA_14genomes_clustage_graphs/bin108_PAO1.png]

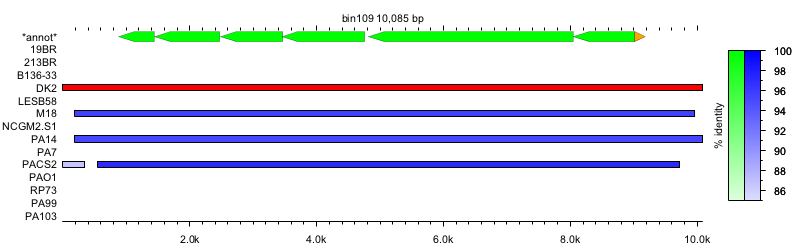

Supplement: Supplementary file 2 — Archive containing output files from ClustAGE analysis of accessory genome sequence files found in Additional file 1. (ZIP 18100 kb) [file 12859_2018_2154_MOESM2_ESM.zip › PA_14genomes_clustage_graphs/bin109_DK2.png]

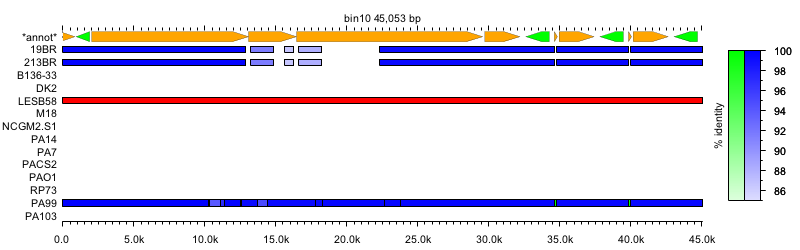

Supplement: Supplementary file 2 — Archive containing output files from ClustAGE analysis of accessory genome sequence files found in Additional file 1. (ZIP 18100 kb) [file 12859_2018_2154_MOESM2_ESM.zip › PA_14genomes_clustage_graphs/bin10_LESB58.png]

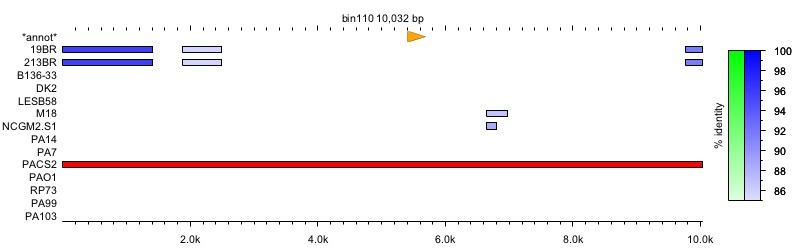

Supplement: Supplementary file 2 — Archive containing output files from ClustAGE analysis of accessory genome sequence files found in Additional file 1. (ZIP 18100 kb) [file 12859_2018_2154_MOESM2_ESM.zip › PA_14genomes_clustage_graphs/bin110_PACS2.png]

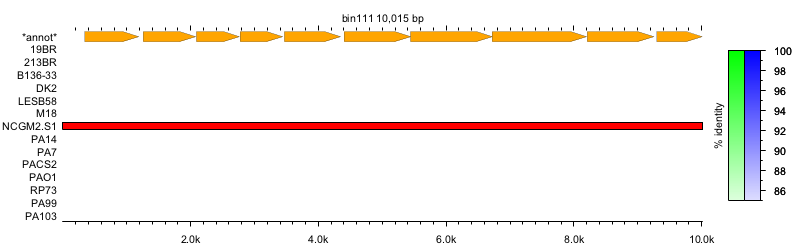

Supplement: Supplementary file 2 — Archive containing output files from ClustAGE analysis of accessory genome sequence files found in Additional file 1. (ZIP 18100 kb) [file 12859_2018_2154_MOESM2_ESM.zip › PA_14genomes_clustage_graphs/bin111_NCGM2.S1.png]

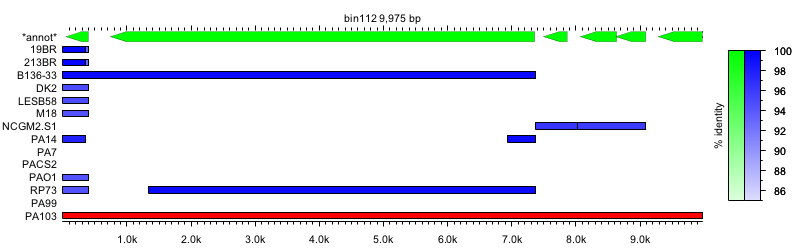

Supplement: Supplementary file 2 — Archive containing output files from ClustAGE analysis of accessory genome sequence files found in Additional file 1. (ZIP 18100 kb) [file 12859_2018_2154_MOESM2_ESM.zip › PA_14genomes_clustage_graphs/bin112_PA103.png]

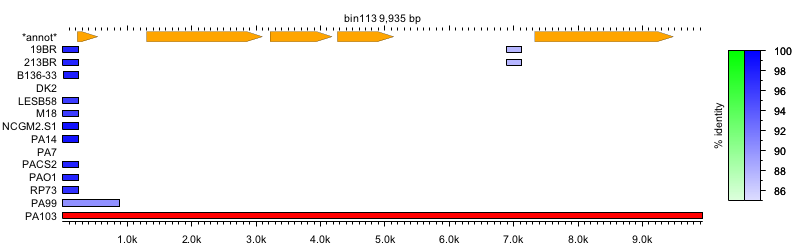

Supplement: Supplementary file 2 — Archive containing output files from ClustAGE analysis of accessory genome sequence files found in Additional file 1. (ZIP 18100 kb) [file 12859_2018_2154_MOESM2_ESM.zip › PA_14genomes_clustage_graphs/bin113_PA103.png]

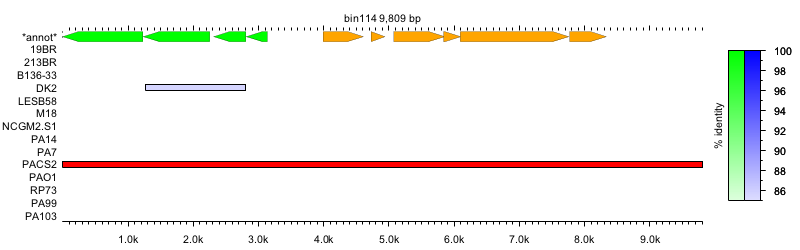

Supplement: Supplementary file 2 — Archive containing output files from ClustAGE analysis of accessory genome sequence files found in Additional file 1. (ZIP 18100 kb) [file 12859_2018_2154_MOESM2_ESM.zip › PA_14genomes_clustage_graphs/bin114_PACS2.png]

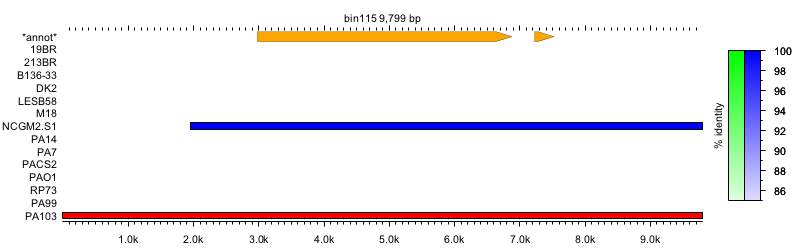

Supplement: Supplementary file 2 — Archive containing output files from ClustAGE analysis of accessory genome sequence files found in Additional file 1. (ZIP 18100 kb) [file 12859_2018_2154_MOESM2_ESM.zip › PA_14genomes_clustage_graphs/bin115_PA103.png]

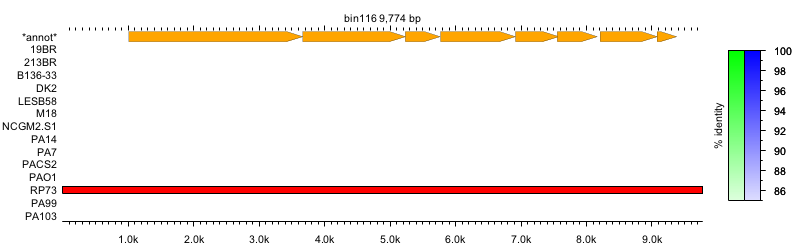

Supplement: Supplementary file 2 — Archive containing output files from ClustAGE analysis of accessory genome sequence files found in Additional file 1. (ZIP 18100 kb) [file 12859_2018_2154_MOESM2_ESM.zip › PA_14genomes_clustage_graphs/bin116_RP73.png]

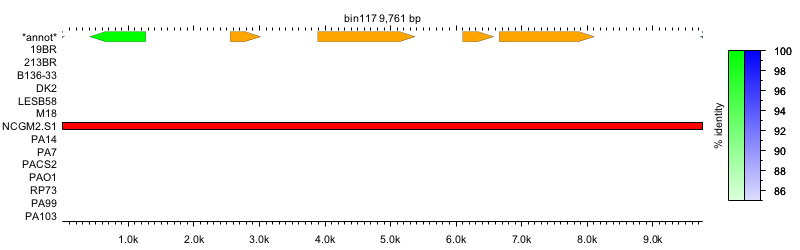

Supplement: Supplementary file 2 — Archive containing output files from ClustAGE analysis of accessory genome sequence files found in Additional file 1. (ZIP 18100 kb) [file 12859_2018_2154_MOESM2_ESM.zip › PA_14genomes_clustage_graphs/bin117_NCGM2.S1.png]

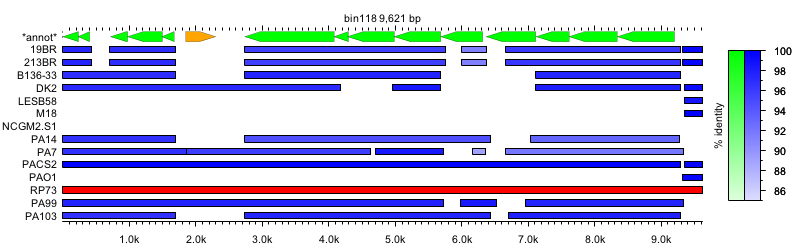

Supplement: Supplementary file 2 — Archive containing output files from ClustAGE analysis of accessory genome sequence files found in Additional file 1. (ZIP 18100 kb) [file 12859_2018_2154_MOESM2_ESM.zip › PA_14genomes_clustage_graphs/bin118_RP73.png]

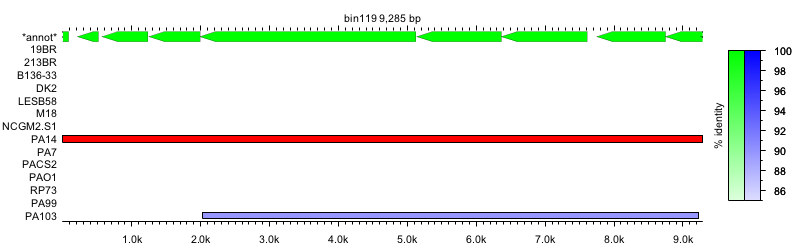

Supplement: Supplementary file 2 — Archive containing output files from ClustAGE analysis of accessory genome sequence files found in Additional file 1. (ZIP 18100 kb) [file 12859_2018_2154_MOESM2_ESM.zip › PA_14genomes_clustage_graphs/bin119_PA14.png]

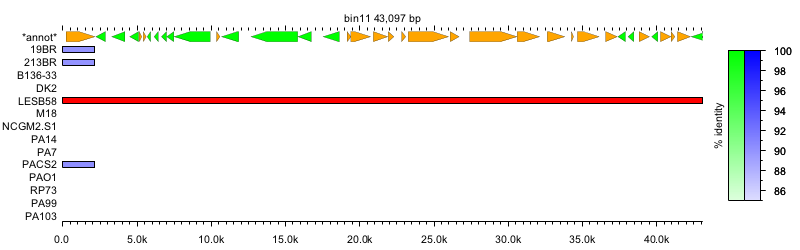

Supplement: Supplementary file 2 — Archive containing output files from ClustAGE analysis of accessory genome sequence files found in Additional file 1. (ZIP 18100 kb) [file 12859_2018_2154_MOESM2_ESM.zip › PA_14genomes_clustage_graphs/bin11_LESB58.png]

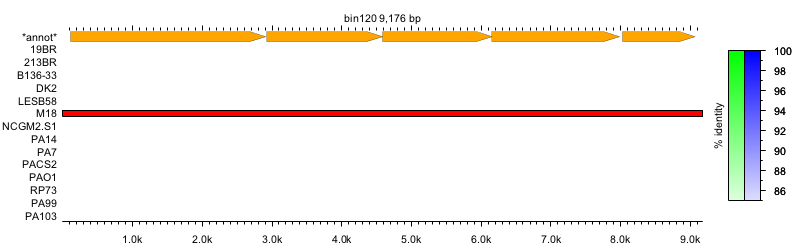

Supplement: Supplementary file 2 — Archive containing output files from ClustAGE analysis of accessory genome sequence files found in Additional file 1. (ZIP 18100 kb) [file 12859_2018_2154_MOESM2_ESM.zip › PA_14genomes_clustage_graphs/bin120_M18.png]

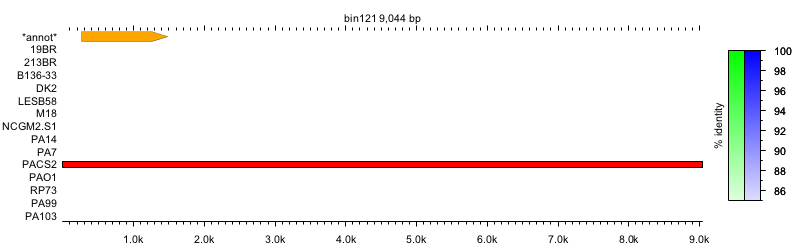

Supplement: Supplementary file 2 — Archive containing output files from ClustAGE analysis of accessory genome sequence files found in Additional file 1. (ZIP 18100 kb) [file 12859_2018_2154_MOESM2_ESM.zip › PA_14genomes_clustage_graphs/bin121_PACS2.png]

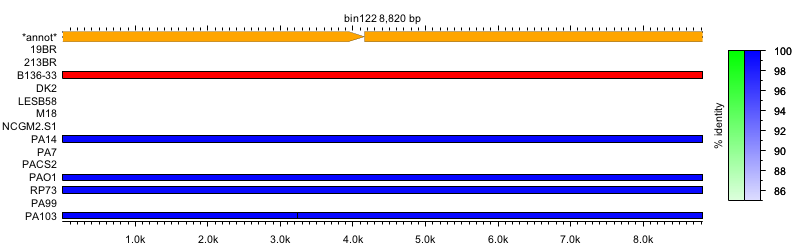

Supplement: Supplementary file 2 — Archive containing output files from ClustAGE analysis of accessory genome sequence files found in Additional file 1. (ZIP 18100 kb) [file 12859_2018_2154_MOESM2_ESM.zip › PA_14genomes_clustage_graphs/bin122_B136-33.png]

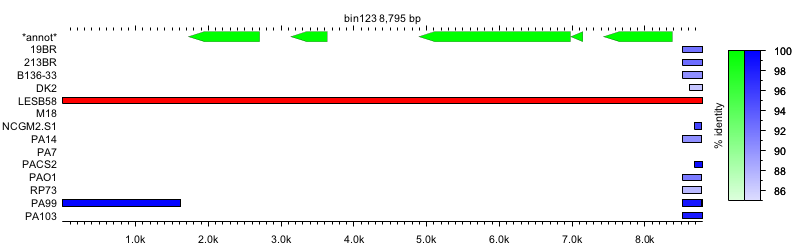

Supplement: Supplementary file 2 — Archive containing output files from ClustAGE analysis of accessory genome sequence files found in Additional file 1. (ZIP 18100 kb) [file 12859_2018_2154_MOESM2_ESM.zip › PA_14genomes_clustage_graphs/bin123_LESB58.png]

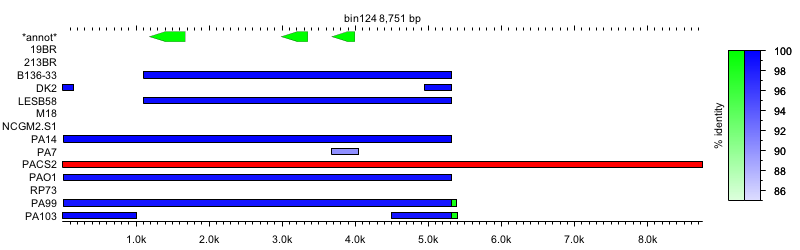

Supplement: Supplementary file 2 — Archive containing output files from ClustAGE analysis of accessory genome sequence files found in Additional file 1. (ZIP 18100 kb) [file 12859_2018_2154_MOESM2_ESM.zip › PA_14genomes_clustage_graphs/bin124_PACS2.png]

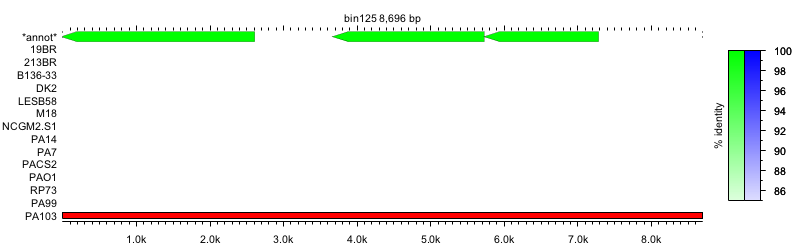

Supplement: Supplementary file 2 — Archive containing output files from ClustAGE analysis of accessory genome sequence files found in Additional file 1. (ZIP 18100 kb) [file 12859_2018_2154_MOESM2_ESM.zip › PA_14genomes_clustage_graphs/bin125_PA103.png]

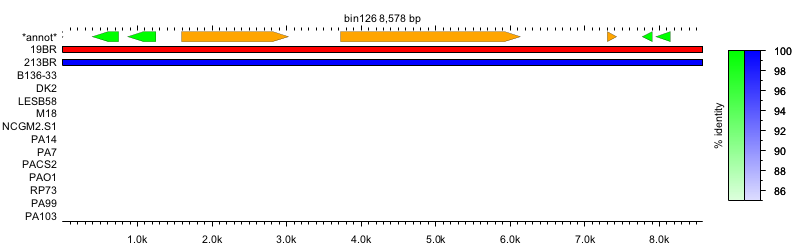

Supplement: Supplementary file 2 — Archive containing output files from ClustAGE analysis of accessory genome sequence files found in Additional file 1. (ZIP 18100 kb) [file 12859_2018_2154_MOESM2_ESM.zip › PA_14genomes_clustage_graphs/bin126_19BR.png]

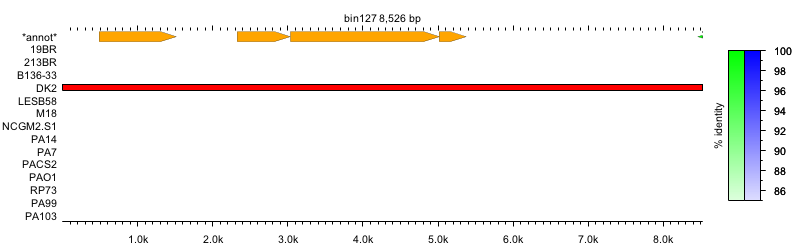

Supplement: Supplementary file 2 — Archive containing output files from ClustAGE analysis of accessory genome sequence files found in Additional file 1. (ZIP 18100 kb) [file 12859_2018_2154_MOESM2_ESM.zip › PA_14genomes_clustage_graphs/bin127_DK2.png]

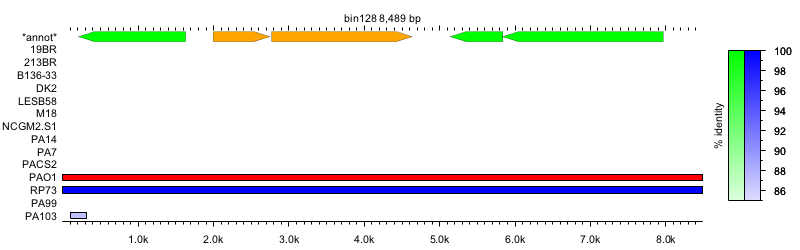

Supplement: Supplementary file 2 — Archive containing output files from ClustAGE analysis of accessory genome sequence files found in Additional file 1. (ZIP 18100 kb) [file 12859_2018_2154_MOESM2_ESM.zip › PA_14genomes_clustage_graphs/bin128_PAO1.png]

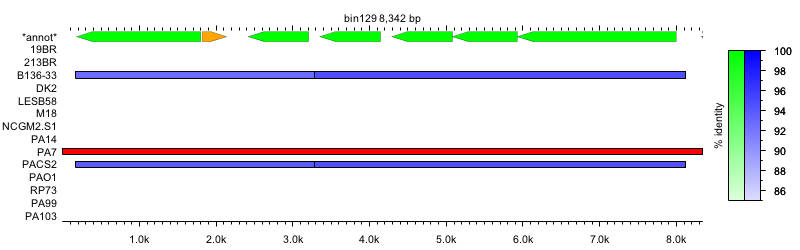

Supplement: Supplementary file 2 — Archive containing output files from ClustAGE analysis of accessory genome sequence files found in Additional file 1. (ZIP 18100 kb) [file 12859_2018_2154_MOESM2_ESM.zip › PA_14genomes_clustage_graphs/bin129_PA7.png]

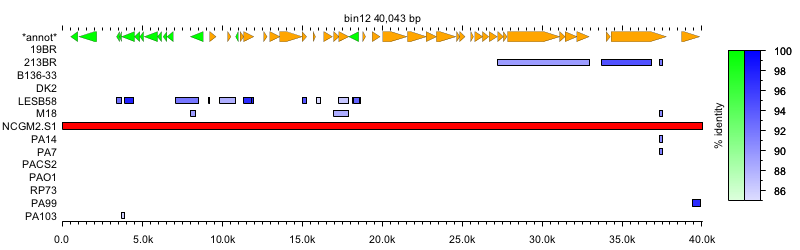

Supplement: Supplementary file 2 — Archive containing output files from ClustAGE analysis of accessory genome sequence files found in Additional file 1. (ZIP 18100 kb) [file 12859_2018_2154_MOESM2_ESM.zip › PA_14genomes_clustage_graphs/bin12_NCGM2.S1.png]

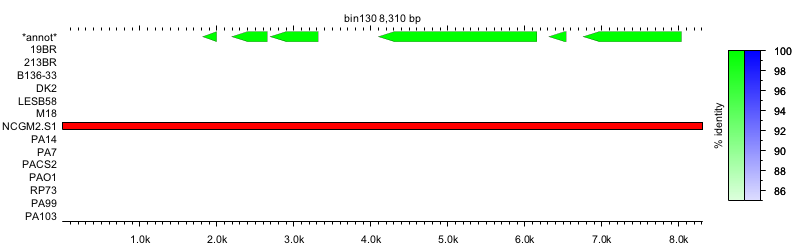

Supplement: Supplementary file 2 — Archive containing output files from ClustAGE analysis of accessory genome sequence files found in Additional file 1. (ZIP 18100 kb) [file 12859_2018_2154_MOESM2_ESM.zip › PA_14genomes_clustage_graphs/bin130_NCGM2.S1.png]

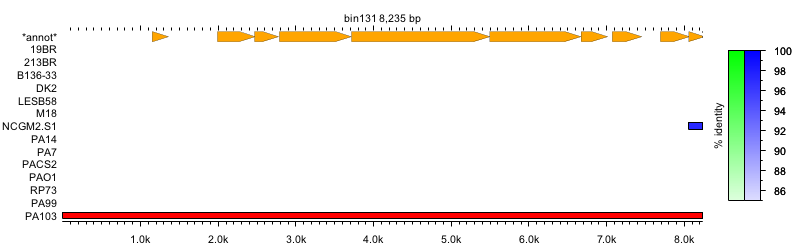

Supplement: Supplementary file 2 — Archive containing output files from ClustAGE analysis of accessory genome sequence files found in Additional file 1. (ZIP 18100 kb) [file 12859_2018_2154_MOESM2_ESM.zip › PA_14genomes_clustage_graphs/bin131_PA103.png]

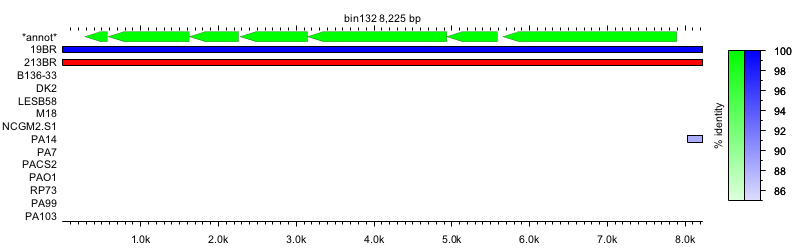

Supplement: Supplementary file 2 — Archive containing output files from ClustAGE analysis of accessory genome sequence files found in Additional file 1. (ZIP 18100 kb) [file 12859_2018_2154_MOESM2_ESM.zip › PA_14genomes_clustage_graphs/bin132_213BR.png]

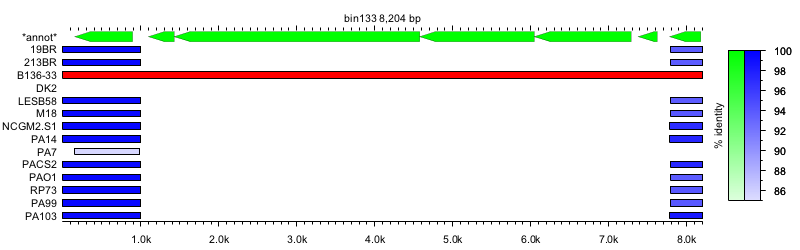

Supplement: Supplementary file 2 — Archive containing output files from ClustAGE analysis of accessory genome sequence files found in Additional file 1. (ZIP 18100 kb) [file 12859_2018_2154_MOESM2_ESM.zip › PA_14genomes_clustage_graphs/bin133_B136-33.png]

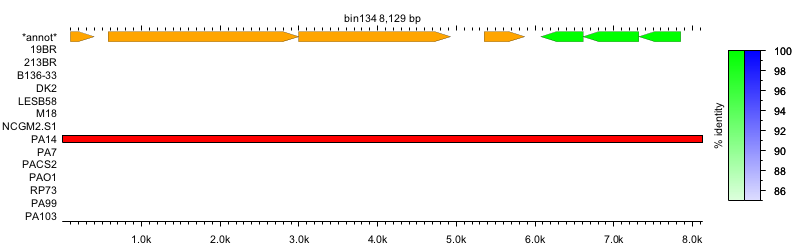

Supplement: Supplementary file 2 — Archive containing output files from ClustAGE analysis of accessory genome sequence files found in Additional file 1. (ZIP 18100 kb) [file 12859_2018_2154_MOESM2_ESM.zip › PA_14genomes_clustage_graphs/bin134_PA14.png]

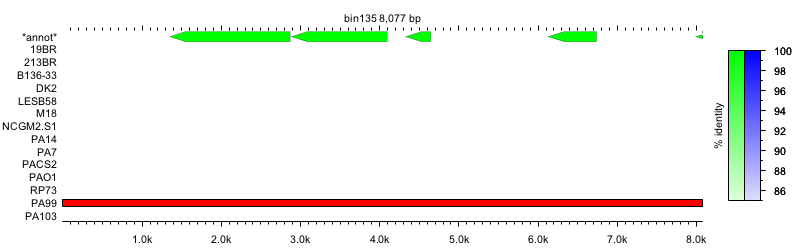

Supplement: Supplementary file 2 — Archive containing output files from ClustAGE analysis of accessory genome sequence files found in Additional file 1. (ZIP 18100 kb) [file 12859_2018_2154_MOESM2_ESM.zip › PA_14genomes_clustage_graphs/bin135_PA99.png]

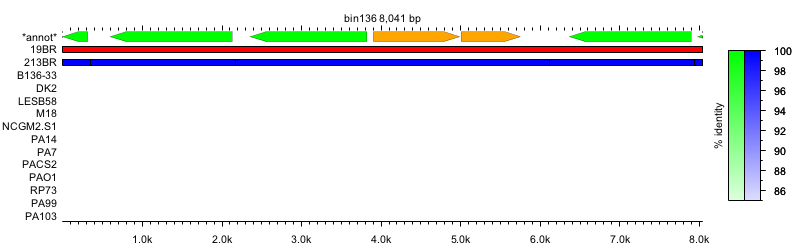

Supplement: Supplementary file 2 — Archive containing output files from ClustAGE analysis of accessory genome sequence files found in Additional file 1. (ZIP 18100 kb) [file 12859_2018_2154_MOESM2_ESM.zip › PA_14genomes_clustage_graphs/bin136_19BR.png]

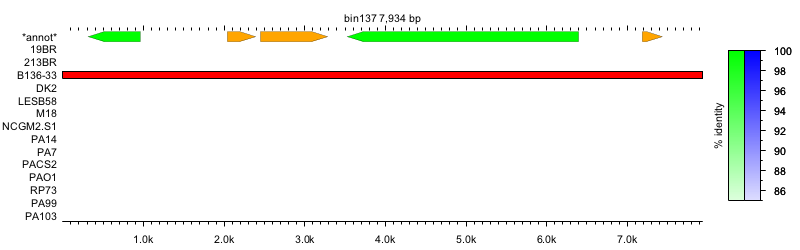

Supplement: Supplementary file 2 — Archive containing output files from ClustAGE analysis of accessory genome sequence files found in Additional file 1. (ZIP 18100 kb) [file 12859_2018_2154_MOESM2_ESM.zip › PA_14genomes_clustage_graphs/bin137_B136-33.png]

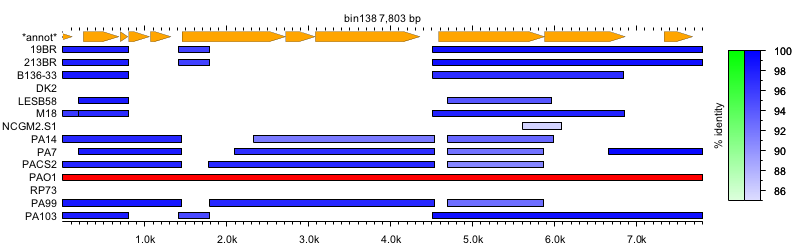

Supplement: Supplementary file 2 — Archive containing output files from ClustAGE analysis of accessory genome sequence files found in Additional file 1. (ZIP 18100 kb) [file 12859_2018_2154_MOESM2_ESM.zip › PA_14genomes_clustage_graphs/bin138_PAO1.png]

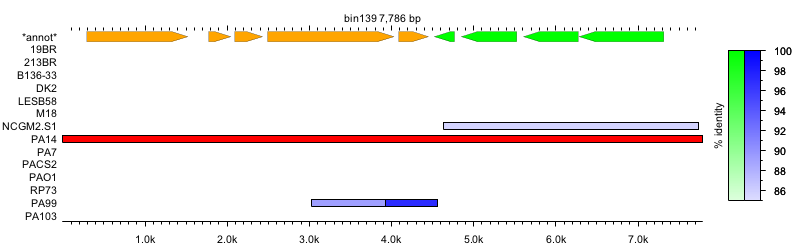

Supplement: Supplementary file 2 — Archive containing output files from ClustAGE analysis of accessory genome sequence files found in Additional file 1. (ZIP 18100 kb) [file 12859_2018_2154_MOESM2_ESM.zip › PA_14genomes_clustage_graphs/bin139_PA14.png]

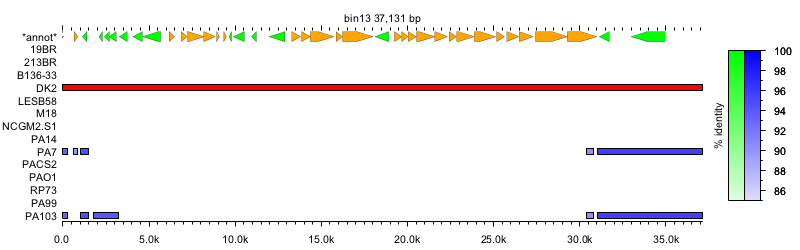

Supplement: Supplementary file 2 — Archive containing output files from ClustAGE analysis of accessory genome sequence files found in Additional file 1. (ZIP 18100 kb) [file 12859_2018_2154_MOESM2_ESM.zip › PA_14genomes_clustage_graphs/bin13_DK2.png]

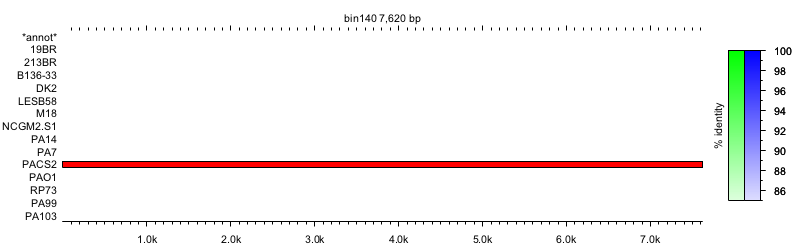

Supplement: Supplementary file 2 — Archive containing output files from ClustAGE analysis of accessory genome sequence files found in Additional file 1. (ZIP 18100 kb) [file 12859_2018_2154_MOESM2_ESM.zip › PA_14genomes_clustage_graphs/bin140_PACS2.png]

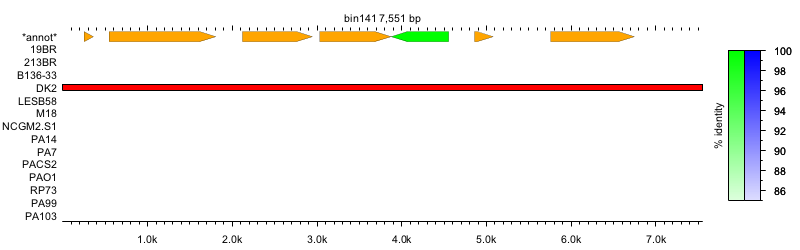

Supplement: Supplementary file 2 — Archive containing output files from ClustAGE analysis of accessory genome sequence files found in Additional file 1. (ZIP 18100 kb) [file 12859_2018_2154_MOESM2_ESM.zip › PA_14genomes_clustage_graphs/bin141_DK2.png]

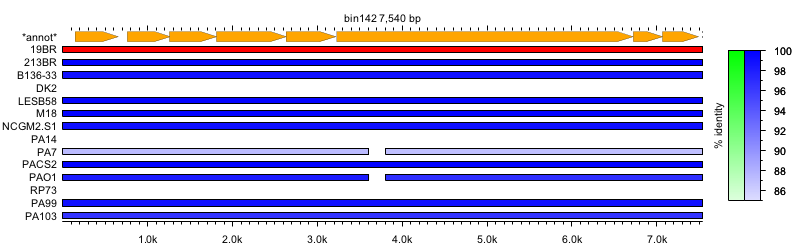

Supplement: Supplementary file 2 — Archive containing output files from ClustAGE analysis of accessory genome sequence files found in Additional file 1. (ZIP 18100 kb) [file 12859_2018_2154_MOESM2_ESM.zip › PA_14genomes_clustage_graphs/bin142_19BR.png]

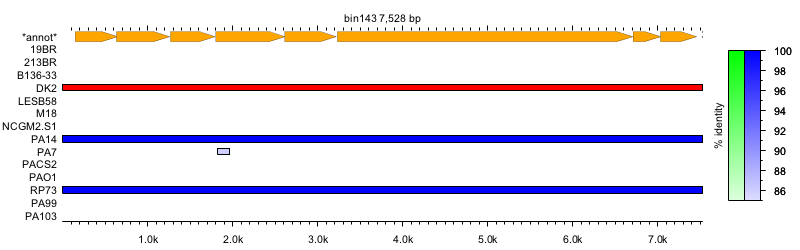

Supplement: Supplementary file 2 — Archive containing output files from ClustAGE analysis of accessory genome sequence files found in Additional file 1. (ZIP 18100 kb) [file 12859_2018_2154_MOESM2_ESM.zip › PA_14genomes_clustage_graphs/bin143_DK2.png]

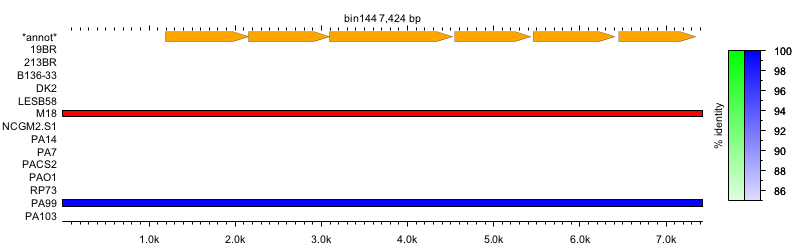

Supplement: Supplementary file 2 — Archive containing output files from ClustAGE analysis of accessory genome sequence files found in Additional file 1. (ZIP 18100 kb) [file 12859_2018_2154_MOESM2_ESM.zip › PA_14genomes_clustage_graphs/bin144_M18.png]

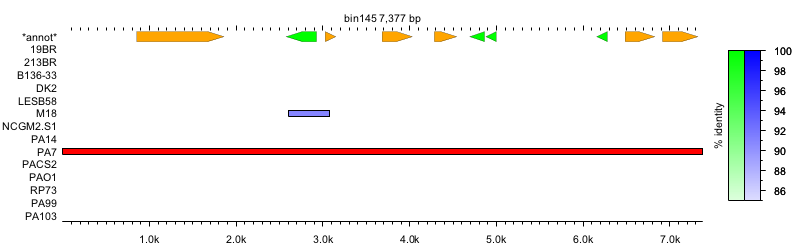

Supplement: Supplementary file 2 — Archive containing output files from ClustAGE analysis of accessory genome sequence files found in Additional file 1. (ZIP 18100 kb) [file 12859_2018_2154_MOESM2_ESM.zip › PA_14genomes_clustage_graphs/bin145_PA7.png]

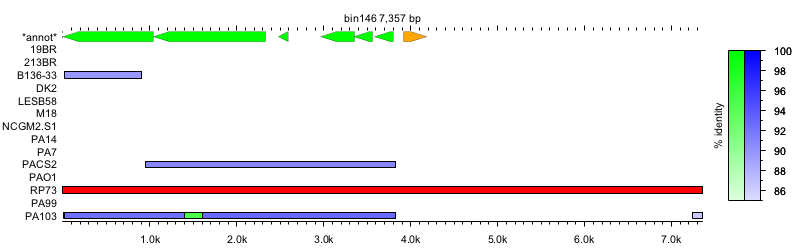

Supplement: Supplementary file 2 — Archive containing output files from ClustAGE analysis of accessory genome sequence files found in Additional file 1. (ZIP 18100 kb) [file 12859_2018_2154_MOESM2_ESM.zip › PA_14genomes_clustage_graphs/bin146_RP73.png]

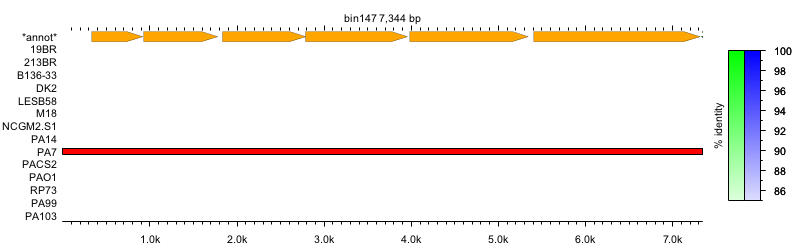

Supplement: Supplementary file 2 — Archive containing output files from ClustAGE analysis of accessory genome sequence files found in Additional file 1. (ZIP 18100 kb) [file 12859_2018_2154_MOESM2_ESM.zip › PA_14genomes_clustage_graphs/bin147_PA7.png]

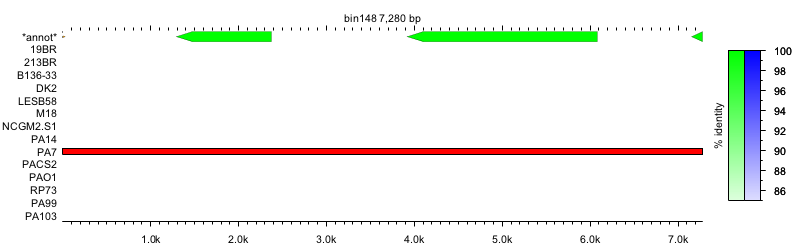

Supplement: Supplementary file 2 — Archive containing output files from ClustAGE analysis of accessory genome sequence files found in Additional file 1. (ZIP 18100 kb) [file 12859_2018_2154_MOESM2_ESM.zip › PA_14genomes_clustage_graphs/bin148_PA7.png]

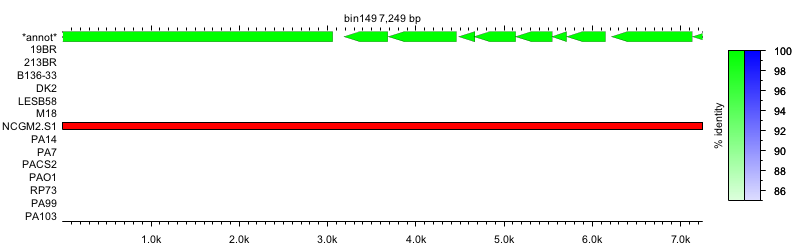

Supplement: Supplementary file 2 — Archive containing output files from ClustAGE analysis of accessory genome sequence files found in Additional file 1. (ZIP 18100 kb) [file 12859_2018_2154_MOESM2_ESM.zip › PA_14genomes_clustage_graphs/bin149_NCGM2.S1.png]

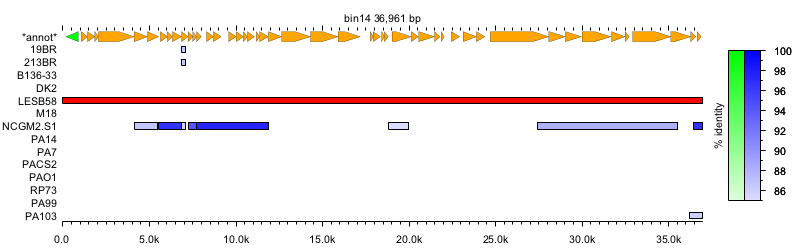

Supplement: Supplementary file 2 — Archive containing output files from ClustAGE analysis of accessory genome sequence files found in Additional file 1. (ZIP 18100 kb) [file 12859_2018_2154_MOESM2_ESM.zip › PA_14genomes_clustage_graphs/bin14_LESB58.png]

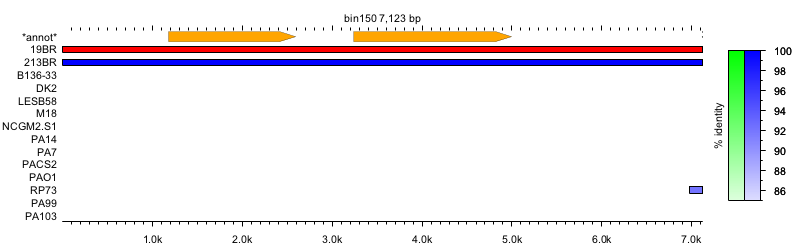

Supplement: Supplementary file 2 — Archive containing output files from ClustAGE analysis of accessory genome sequence files found in Additional file 1. (ZIP 18100 kb) [file 12859_2018_2154_MOESM2_ESM.zip › PA_14genomes_clustage_graphs/bin150_19BR.png]

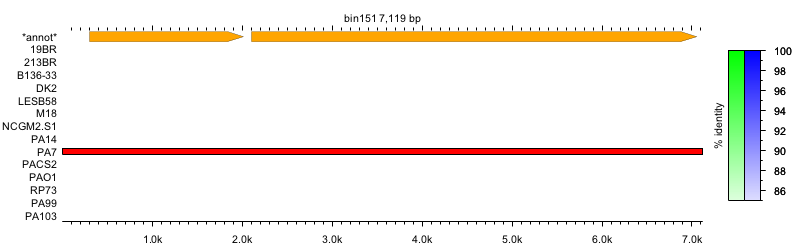

Supplement: Supplementary file 2 — Archive containing output files from ClustAGE analysis of accessory genome sequence files found in Additional file 1. (ZIP 18100 kb) [file 12859_2018_2154_MOESM2_ESM.zip › PA_14genomes_clustage_graphs/bin151_PA7.png]

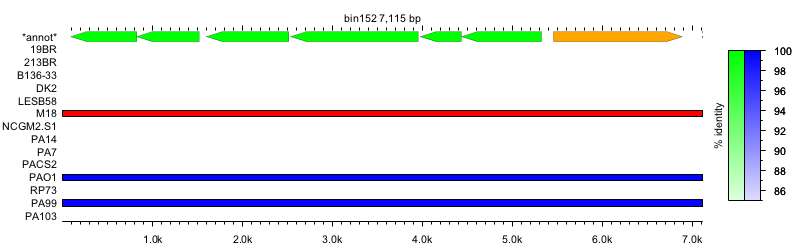

Supplement: Supplementary file 2 — Archive containing output files from ClustAGE analysis of accessory genome sequence files found in Additional file 1. (ZIP 18100 kb) [file 12859_2018_2154_MOESM2_ESM.zip › PA_14genomes_clustage_graphs/bin152_M18.png]

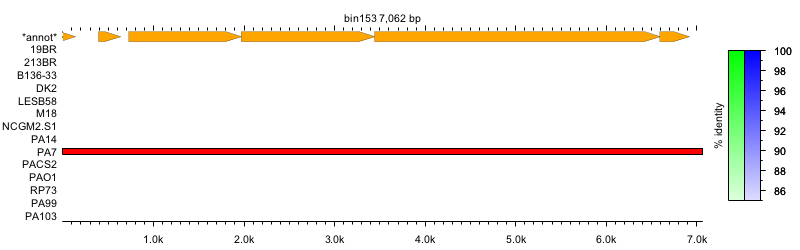

Supplement: Supplementary file 2 — Archive containing output files from ClustAGE analysis of accessory genome sequence files found in Additional file 1. (ZIP 18100 kb) [file 12859_2018_2154_MOESM2_ESM.zip › PA_14genomes_clustage_graphs/bin153_PA7.png]

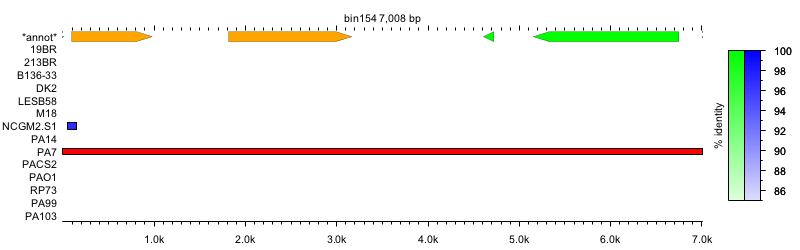

Supplement: Supplementary file 2 — Archive containing output files from ClustAGE analysis of accessory genome sequence files found in Additional file 1. (ZIP 18100 kb) [file 12859_2018_2154_MOESM2_ESM.zip › PA_14genomes_clustage_graphs/bin154_PA7.png]

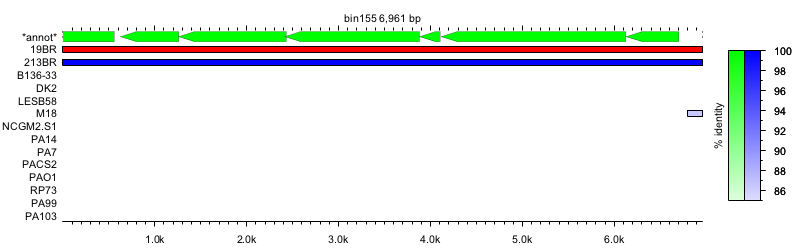

Supplement: Supplementary file 2 — Archive containing output files from ClustAGE analysis of accessory genome sequence files found in Additional file 1. (ZIP 18100 kb) [file 12859_2018_2154_MOESM2_ESM.zip › PA_14genomes_clustage_graphs/bin155_19BR.png]

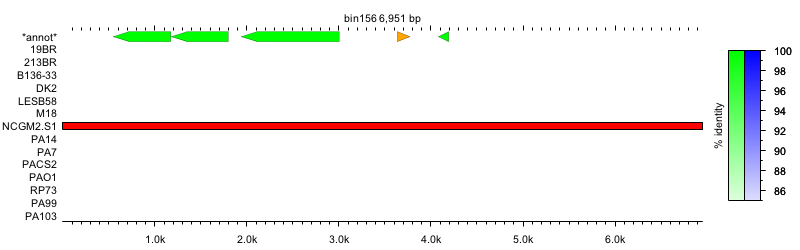

Supplement: Supplementary file 2 — Archive containing output files from ClustAGE analysis of accessory genome sequence files found in Additional file 1. (ZIP 18100 kb) [file 12859_2018_2154_MOESM2_ESM.zip › PA_14genomes_clustage_graphs/bin156_NCGM2.S1.png]

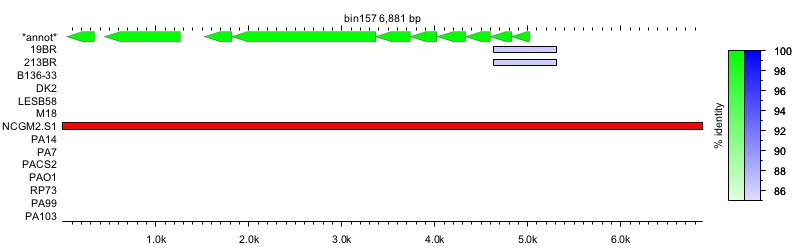

Supplement: Supplementary file 2 — Archive containing output files from ClustAGE analysis of accessory genome sequence files found in Additional file 1. (ZIP 18100 kb) [file 12859_2018_2154_MOESM2_ESM.zip › PA_14genomes_clustage_graphs/bin157_NCGM2.S1.png]

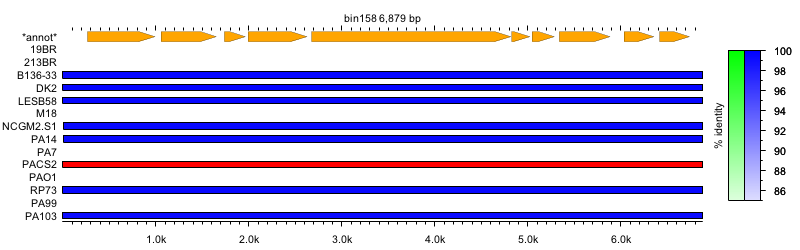

Supplement: Supplementary file 2 — Archive containing output files from ClustAGE analysis of accessory genome sequence files found in Additional file 1. (ZIP 18100 kb) [file 12859_2018_2154_MOESM2_ESM.zip › PA_14genomes_clustage_graphs/bin158_PACS2.png]

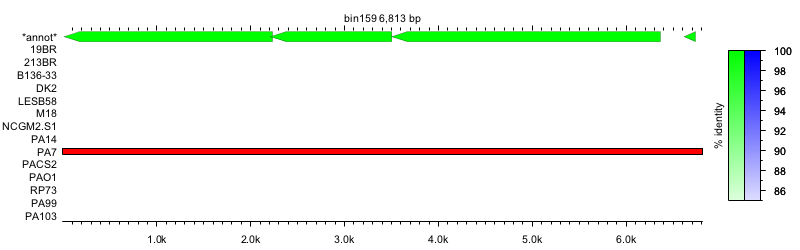

Supplement: Supplementary file 2 — Archive containing output files from ClustAGE analysis of accessory genome sequence files found in Additional file 1. (ZIP 18100 kb) [file 12859_2018_2154_MOESM2_ESM.zip › PA_14genomes_clustage_graphs/bin159_PA7.png]

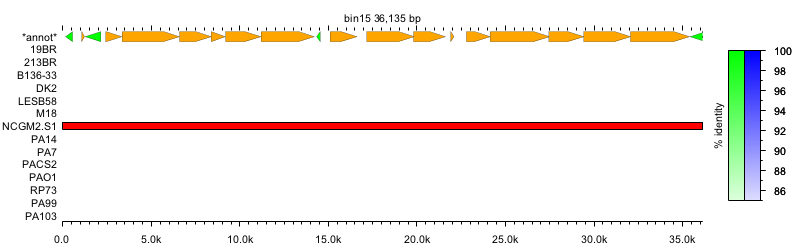

Supplement: Supplementary file 2 — Archive containing output files from ClustAGE analysis of accessory genome sequence files found in Additional file 1. (ZIP 18100 kb) [file 12859_2018_2154_MOESM2_ESM.zip › PA_14genomes_clustage_graphs/bin15_NCGM2.S1.png]

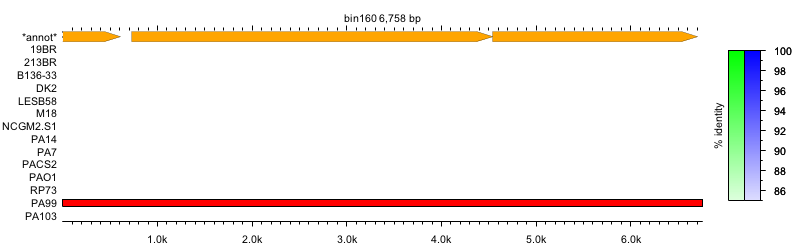

Supplement: Supplementary file 2 — Archive containing output files from ClustAGE analysis of accessory genome sequence files found in Additional file 1. (ZIP 18100 kb) [file 12859_2018_2154_MOESM2_ESM.zip › PA_14genomes_clustage_graphs/bin160_PA99.png]

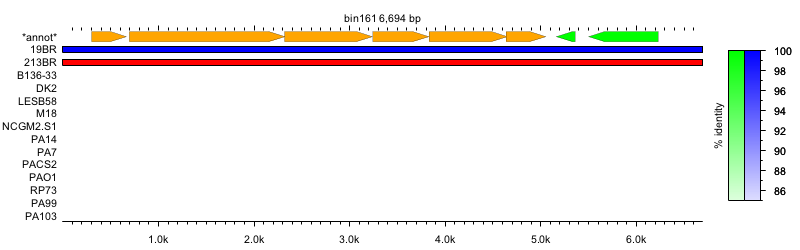

Supplement: Supplementary file 2 — Archive containing output files from ClustAGE analysis of accessory genome sequence files found in Additional file 1. (ZIP 18100 kb) [file 12859_2018_2154_MOESM2_ESM.zip › PA_14genomes_clustage_graphs/bin161_213BR.png]

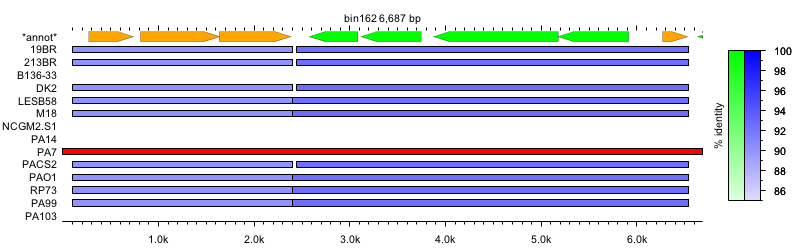

Supplement: Supplementary file 2 — Archive containing output files from ClustAGE analysis of accessory genome sequence files found in Additional file 1. (ZIP 18100 kb) [file 12859_2018_2154_MOESM2_ESM.zip › PA_14genomes_clustage_graphs/bin162_PA7.png]

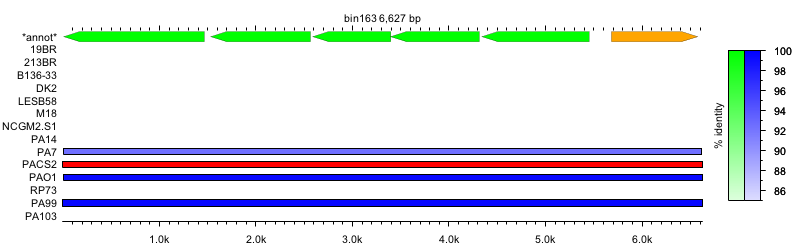

Supplement: Supplementary file 2 — Archive containing output files from ClustAGE analysis of accessory genome sequence files found in Additional file 1. (ZIP 18100 kb) [file 12859_2018_2154_MOESM2_ESM.zip › PA_14genomes_clustage_graphs/bin163_PACS2.png]

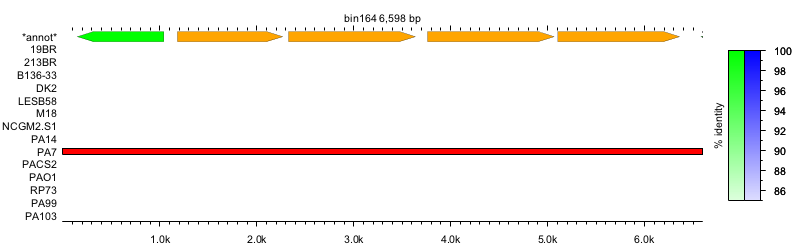

Supplement: Supplementary file 2 — Archive containing output files from ClustAGE analysis of accessory genome sequence files found in Additional file 1. (ZIP 18100 kb) [file 12859_2018_2154_MOESM2_ESM.zip › PA_14genomes_clustage_graphs/bin164_PA7.png]

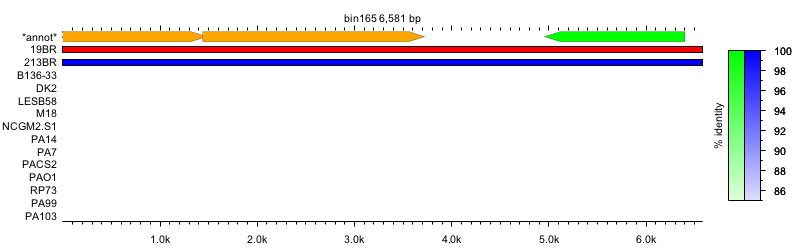

Supplement: Supplementary file 2 — Archive containing output files from ClustAGE analysis of accessory genome sequence files found in Additional file 1. (ZIP 18100 kb) [file 12859_2018_2154_MOESM2_ESM.zip › PA_14genomes_clustage_graphs/bin165_19BR.png]

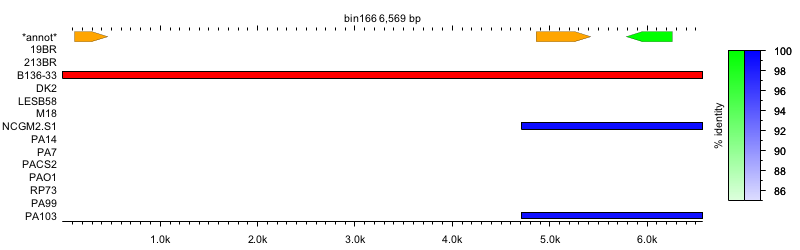

Supplement: Supplementary file 2 — Archive containing output files from ClustAGE analysis of accessory genome sequence files found in Additional file 1. (ZIP 18100 kb) [file 12859_2018_2154_MOESM2_ESM.zip › PA_14genomes_clustage_graphs/bin166_B136-33.png]

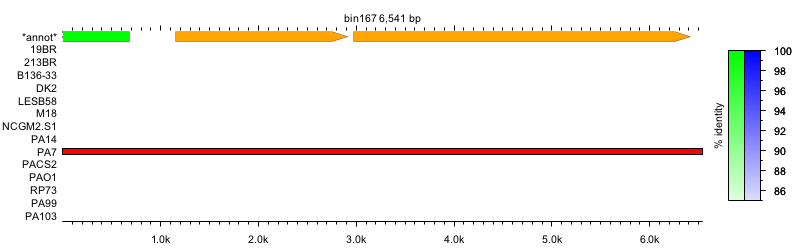

Supplement: Supplementary file 2 — Archive containing output files from ClustAGE analysis of accessory genome sequence files found in Additional file 1. (ZIP 18100 kb) [file 12859_2018_2154_MOESM2_ESM.zip › PA_14genomes_clustage_graphs/bin167_PA7.png]

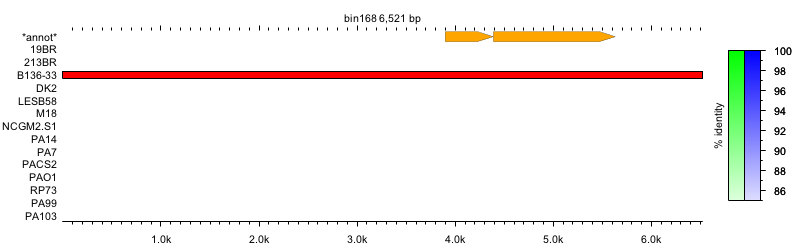

Supplement: Supplementary file 2 — Archive containing output files from ClustAGE analysis of accessory genome sequence files found in Additional file 1. (ZIP 18100 kb) [file 12859_2018_2154_MOESM2_ESM.zip › PA_14genomes_clustage_graphs/bin168_B136-33.png]

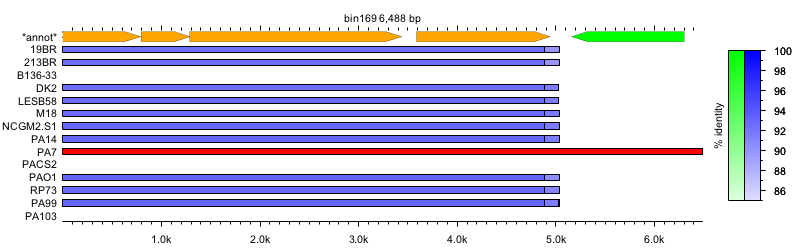

Supplement: Supplementary file 2 — Archive containing output files from ClustAGE analysis of accessory genome sequence files found in Additional file 1. (ZIP 18100 kb) [file 12859_2018_2154_MOESM2_ESM.zip › PA_14genomes_clustage_graphs/bin169_PA7.png]

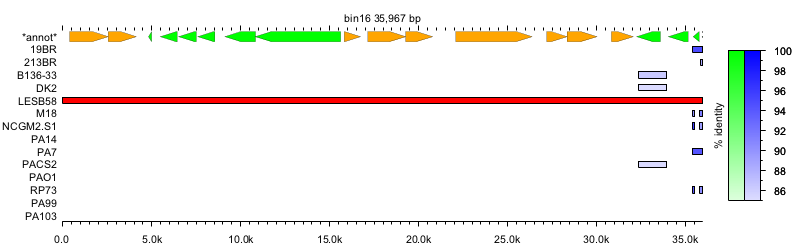

Supplement: Supplementary file 2 — Archive containing output files from ClustAGE analysis of accessory genome sequence files found in Additional file 1. (ZIP 18100 kb) [file 12859_2018_2154_MOESM2_ESM.zip › PA_14genomes_clustage_graphs/bin16_LESB58.png]

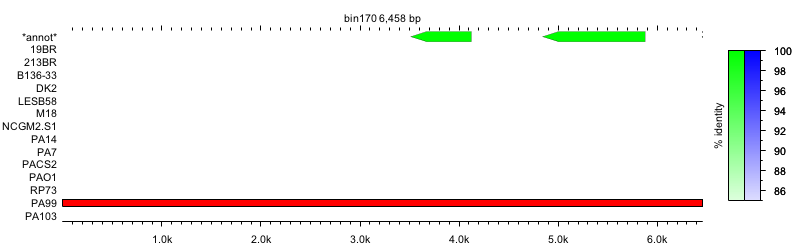

Supplement: Supplementary file 2 — Archive containing output files from ClustAGE analysis of accessory genome sequence files found in Additional file 1. (ZIP 18100 kb) [file 12859_2018_2154_MOESM2_ESM.zip › PA_14genomes_clustage_graphs/bin170_PA99.png]

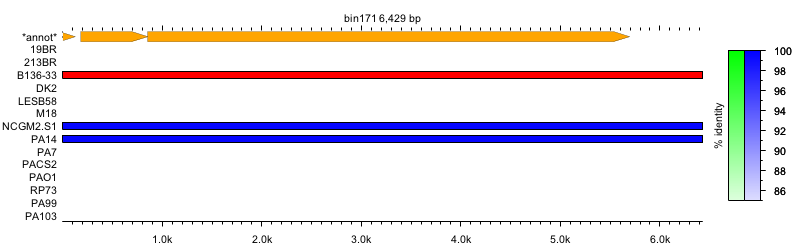

Supplement: Supplementary file 2 — Archive containing output files from ClustAGE analysis of accessory genome sequence files found in Additional file 1. (ZIP 18100 kb) [file 12859_2018_2154_MOESM2_ESM.zip › PA_14genomes_clustage_graphs/bin171_B136-33.png]

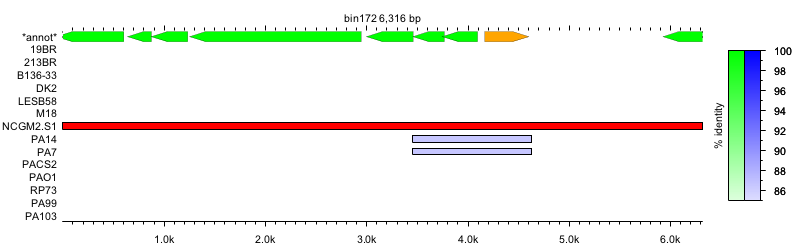

Supplement: Supplementary file 2 — Archive containing output files from ClustAGE analysis of accessory genome sequence files found in Additional file 1. (ZIP 18100 kb) [file 12859_2018_2154_MOESM2_ESM.zip › PA_14genomes_clustage_graphs/bin172_NCGM2.S1.png]

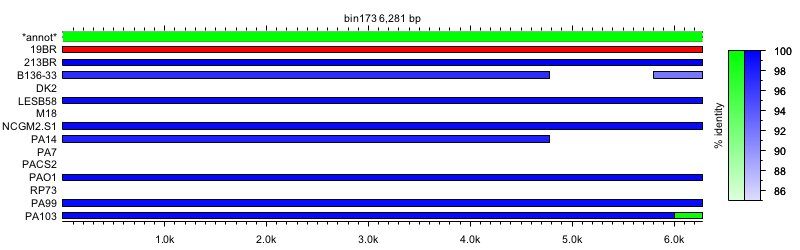

Supplement: Supplementary file 2 — Archive containing output files from ClustAGE analysis of accessory genome sequence files found in Additional file 1. (ZIP 18100 kb) [file 12859_2018_2154_MOESM2_ESM.zip › PA_14genomes_clustage_graphs/bin173_19BR.png]

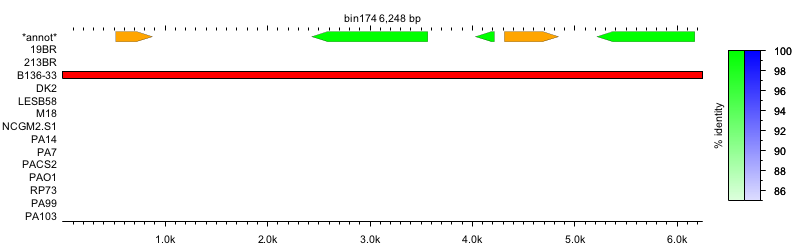

Supplement: Supplementary file 2 — Archive containing output files from ClustAGE analysis of accessory genome sequence files found in Additional file 1. (ZIP 18100 kb) [file 12859_2018_2154_MOESM2_ESM.zip › PA_14genomes_clustage_graphs/bin174_B136-33.png]

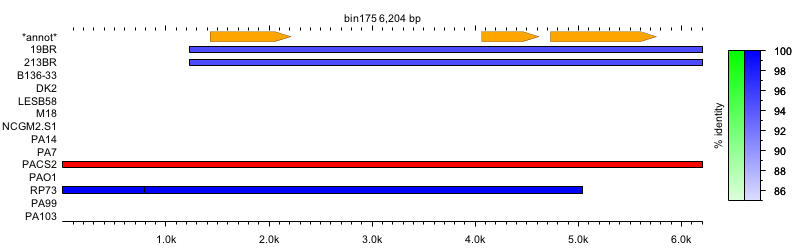

Supplement: Supplementary file 2 — Archive containing output files from ClustAGE analysis of accessory genome sequence files found in Additional file 1. (ZIP 18100 kb) [file 12859_2018_2154_MOESM2_ESM.zip › PA_14genomes_clustage_graphs/bin175_PACS2.png]

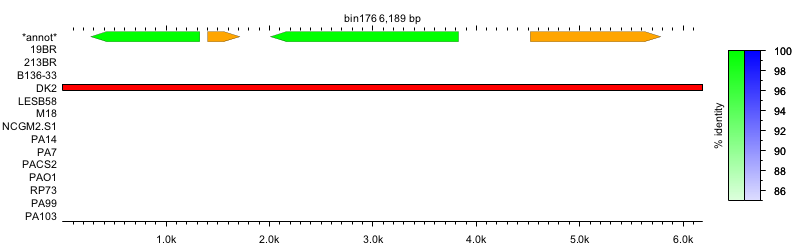

Supplement: Supplementary file 2 — Archive containing output files from ClustAGE analysis of accessory genome sequence files found in Additional file 1. (ZIP 18100 kb) [file 12859_2018_2154_MOESM2_ESM.zip › PA_14genomes_clustage_graphs/bin176_DK2.png]

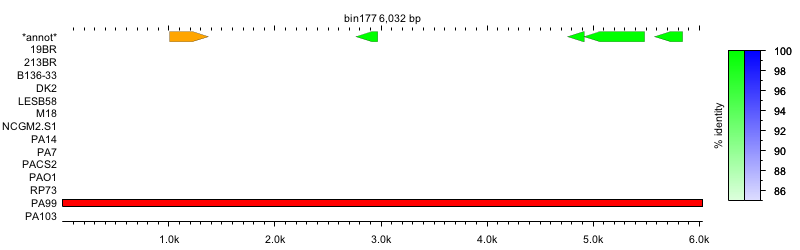

Supplement: Supplementary file 2 — Archive containing output files from ClustAGE analysis of accessory genome sequence files found in Additional file 1. (ZIP 18100 kb) [file 12859_2018_2154_MOESM2_ESM.zip › PA_14genomes_clustage_graphs/bin177_PA99.png]

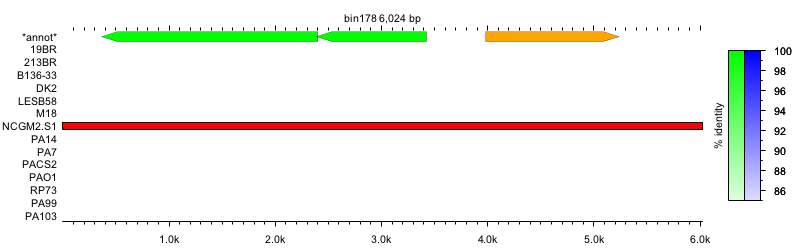

Supplement: Supplementary file 2 — Archive containing output files from ClustAGE analysis of accessory genome sequence files found in Additional file 1. (ZIP 18100 kb) [file 12859_2018_2154_MOESM2_ESM.zip › PA_14genomes_clustage_graphs/bin178_NCGM2.S1.png]

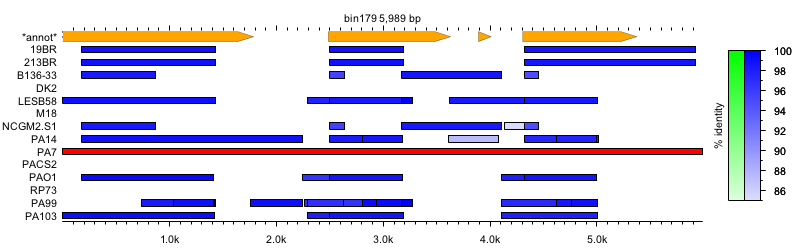

Supplement: Supplementary file 2 — Archive containing output files from ClustAGE analysis of accessory genome sequence files found in Additional file 1. (ZIP 18100 kb) [file 12859_2018_2154_MOESM2_ESM.zip › PA_14genomes_clustage_graphs/bin179_PA7.png]

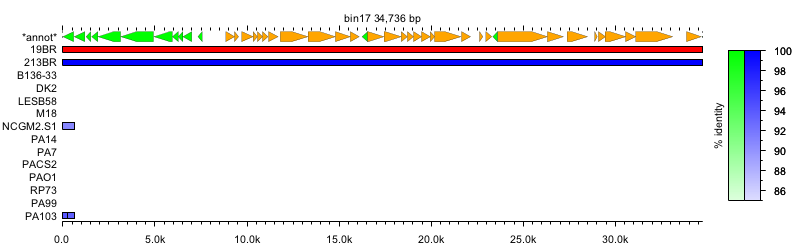

Supplement: Supplementary file 2 — Archive containing output files from ClustAGE analysis of accessory genome sequence files found in Additional file 1. (ZIP 18100 kb) [file 12859_2018_2154_MOESM2_ESM.zip › PA_14genomes_clustage_graphs/bin17_19BR.png]

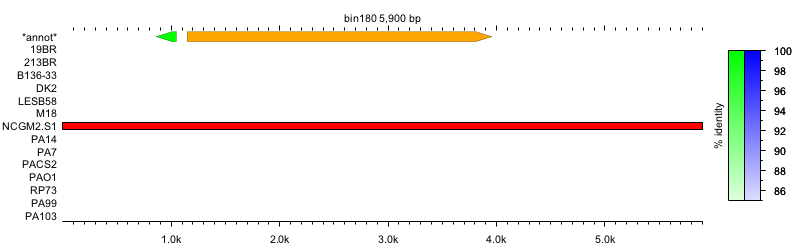

Supplement: Supplementary file 2 — Archive containing output files from ClustAGE analysis of accessory genome sequence files found in Additional file 1. (ZIP 18100 kb) [file 12859_2018_2154_MOESM2_ESM.zip › PA_14genomes_clustage_graphs/bin180_NCGM2.S1.png]

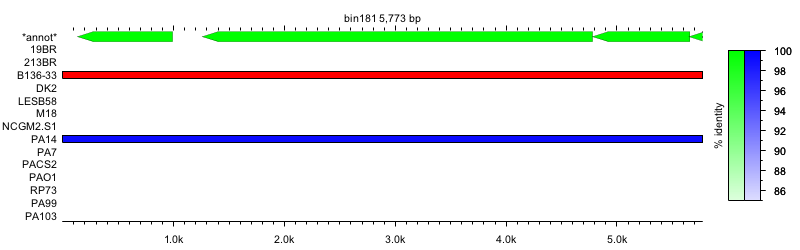

Supplement: Supplementary file 2 — Archive containing output files from ClustAGE analysis of accessory genome sequence files found in Additional file 1. (ZIP 18100 kb) [file 12859_2018_2154_MOESM2_ESM.zip › PA_14genomes_clustage_graphs/bin181_B136-33.png]

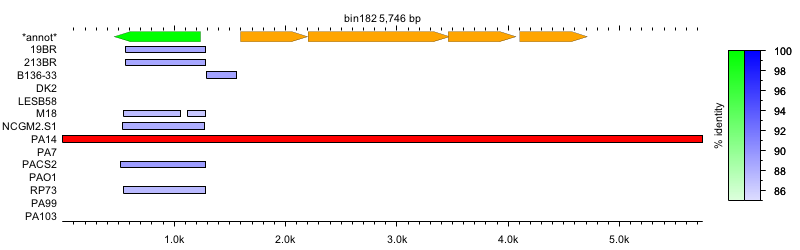

Supplement: Supplementary file 2 — Archive containing output files from ClustAGE analysis of accessory genome sequence files found in Additional file 1. (ZIP 18100 kb) [file 12859_2018_2154_MOESM2_ESM.zip › PA_14genomes_clustage_graphs/bin182_PA14.png]

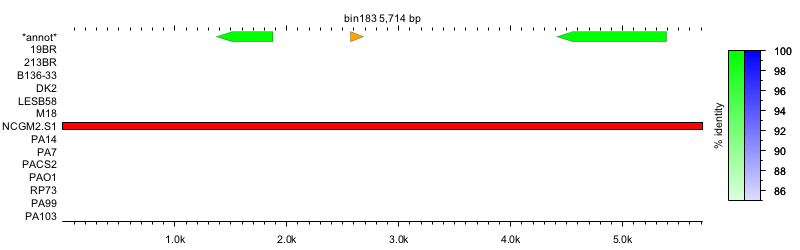

Supplement: Supplementary file 2 — Archive containing output files from ClustAGE analysis of accessory genome sequence files found in Additional file 1. (ZIP 18100 kb) [file 12859_2018_2154_MOESM2_ESM.zip › PA_14genomes_clustage_graphs/bin183_NCGM2.S1.png]

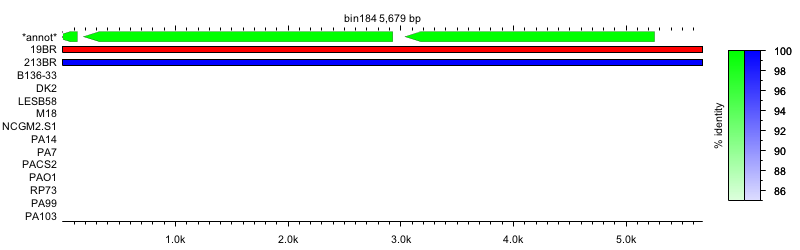

Supplement: Supplementary file 2 — Archive containing output files from ClustAGE analysis of accessory genome sequence files found in Additional file 1. (ZIP 18100 kb) [file 12859_2018_2154_MOESM2_ESM.zip › PA_14genomes_clustage_graphs/bin184_19BR.png]

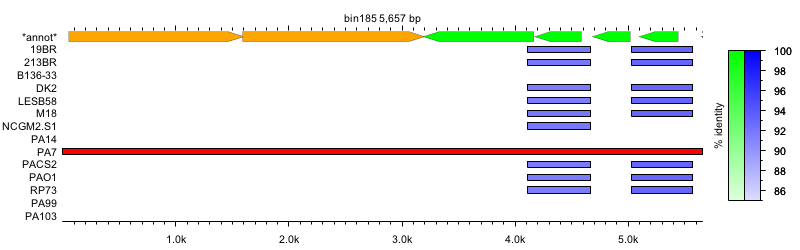

Supplement: Supplementary file 2 — Archive containing output files from ClustAGE analysis of accessory genome sequence files found in Additional file 1. (ZIP 18100 kb) [file 12859_2018_2154_MOESM2_ESM.zip › PA_14genomes_clustage_graphs/bin185_PA7.png]

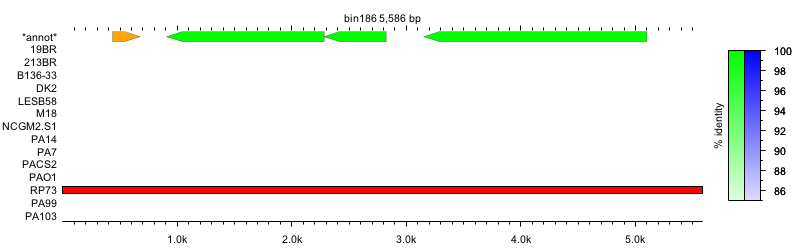

Supplement: Supplementary file 2 — Archive containing output files from ClustAGE analysis of accessory genome sequence files found in Additional file 1. (ZIP 18100 kb) [file 12859_2018_2154_MOESM2_ESM.zip › PA_14genomes_clustage_graphs/bin186_RP73.png]

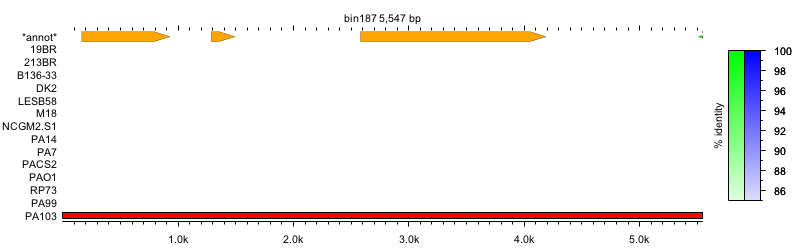

Supplement: Supplementary file 2 — Archive containing output files from ClustAGE analysis of accessory genome sequence files found in Additional file 1. (ZIP 18100 kb) [file 12859_2018_2154_MOESM2_ESM.zip › PA_14genomes_clustage_graphs/bin187_PA103.png]

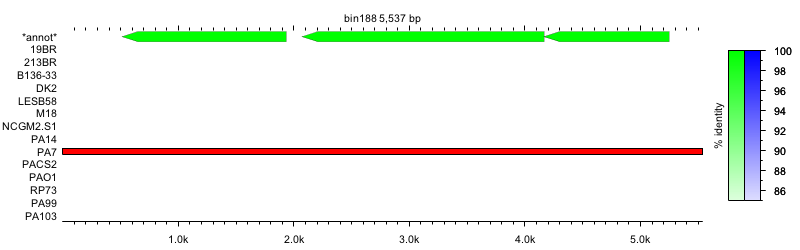

Supplement: Supplementary file 2 — Archive containing output files from ClustAGE analysis of accessory genome sequence files found in Additional file 1. (ZIP 18100 kb) [file 12859_2018_2154_MOESM2_ESM.zip › PA_14genomes_clustage_graphs/bin188_PA7.png]

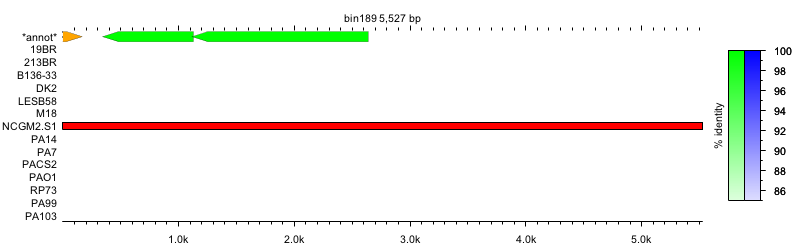

Supplement: Supplementary file 2 — Archive containing output files from ClustAGE analysis of accessory genome sequence files found in Additional file 1. (ZIP 18100 kb) [file 12859_2018_2154_MOESM2_ESM.zip › PA_14genomes_clustage_graphs/bin189_NCGM2.S1.png]

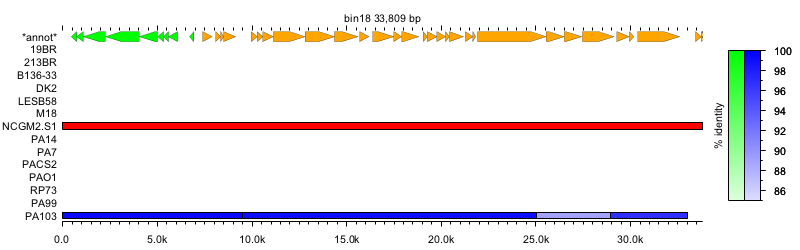

Supplement: Supplementary file 2 — Archive containing output files from ClustAGE analysis of accessory genome sequence files found in Additional file 1. (ZIP 18100 kb) [file 12859_2018_2154_MOESM2_ESM.zip › PA_14genomes_clustage_graphs/bin18_NCGM2.S1.png]

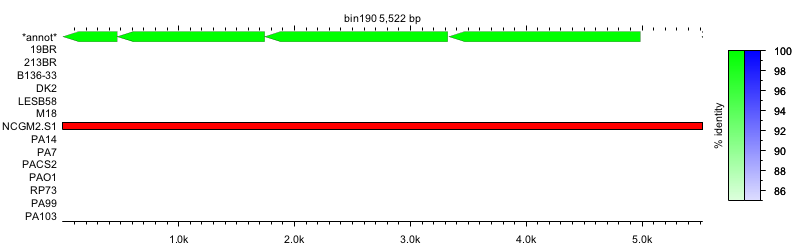

Supplement: Supplementary file 2 — Archive containing output files from ClustAGE analysis of accessory genome sequence files found in Additional file 1. (ZIP 18100 kb) [file 12859_2018_2154_MOESM2_ESM.zip › PA_14genomes_clustage_graphs/bin190_NCGM2.S1.png]
