# Supplementary material for: ClustAGE: a tool for clustering and distribution analysis of bacterial accessory genomic elements
Source: BMC Bioinformatics. 2018 Apr 20;19:150. doi: 10.1186/s12859-018-2154-x (PMC5910555; doi:10.1186/s12859-018-2154-x)
Supplement: Supplementary file 5 — ClustAGE gene distribution analysis. (DOCX 33 kb) [file 12859_2018_2154_MOESM5_ESM.docx]

**Supplemental Methods:**

**ClustAGE gene distribution analysis:**

To determine the relative accuracy of calling the presence or absence of a bin reference gene within the accessory sequence of the other genomes aligned by ClustAGE, presence of orthologous genes between the accessory genomes of all 14 *P. aeruginosa* strains in this analysis was inferred by reciprocal best BLAST hits (RBB) [1,2] and compared to ClustAGE output. Specifically, for each strain the protein sequences of coding sequences identified by AGEnt as being at least 50%, by nucleotide sequence length, present in the accessory genome were extracted. Accessory gene sequences for each strain were then queried against the accessory gene sets of each of the other 13 strains using the blastp function of BLAST+ v2.2.27 [3-5] with a maximum E value cutoff of 1 x 10^-6^ and the “use_sw_tback” option to compute locally optimal Smith-Waterman alignments.

RBB hits were determined using previously-defined criteria [6]. Briefly, alignment results for each query-genome-to-subject-genome comparison were sorted by score from highest to lowest. For each query protein, the first hit where at least 50% of the query gene was covered by the subject gene was considered the best hit. If the next hit against the query protein had the same score and minimum gene coverage, the query protein was given more than one best hit. The same procedure was then performed in the opposite direction, i.e. the subject genome was used as the query and the query genome as the subject. To consider a pair of proteins in two genomes as reciprocal best hits, for each best hit found against a query protein in the forward analysis, this query protein would have to be found as a best hit against the same protein(s) in the opposite analysis. To improve the comparability between RBB results and ClustAGE results, only RBB hits with at least 85% sequence amino acid sequence identity were counted as orthologs. This cutoff was chosen to approximate the minimum 85% nucleotide sequence identity filter for AGE alignments used in the main ClustAGE analyses.

For the purposes of comparison between ClustAGE and RBB results, a gene present in a ClustAGE bin reference AGE was considered to be present within the accessory genome of one of the other 13 strains if at least 50% of the length of the gene was covered by aligned sequence from that strain’s accessory genome. To examine instances where ClustAGE indicated the presence of an orotholog of a gene in the accessory genome of a strain that was not shown in the RBB analysis of that strain pair, the protein sequence of the gene from the bin reference strain was used as the query sequence in a translated BLAST analysis (tblastn) [3,4] against the nucleotide accessory genome sequence of the strain found to be discrepant in the ClustAGE vs. RBB comparison. The query gene was considered to be present in the accessory genome of the comparator strain if at least 50% of the gene sequence length was covered by tblastn alignment.

**Scalability and Computational Efficiency**

To measure the scalability and computational performance of the ClustAGE algorithm, the software was run on test sets of accessory elements from *P. aeruginosa* genomes. A total of 973 sequence and annotation files representing all annotated complete and draft *P. aeruginosa* genomes deposited in the NCBI database as of Oct 26, 2017 were downloaded in genbank flat file format. From this collection, random subsets of 10, 25, 50, 100, 150, and 250 genome sequences were chosen. Each genome subset selection was repeated 5 times for a total of 30 genome sequence sets. The accessory genome component of each sequence in the sets was identified with AGEnt using the core genome sequence of the 12 reference PA genomes generated by Spine. ClustAGE analysis was performed for the 30 accessory genome sets on a Dell PowerEdge R910 server with 2.00 GHz processors and 128 GB of memory running Ubuntu (v14.04.5 LTS) and on an Apple Mac Pro with 2.4 GHz processors and 64 Gb of RAM running OS X El Capitan (v10.11.6) . ClustAGE was run with the following default settings: annotation information included from AGEnt output using ‘--annot’ option, maximum e-value cutoff of 1x10^-6^, minimum sequence identity of 85%, maximum of 100,000 BLAST alignments, minimum accessory element size of 200 bp, minimum alignment length of 100 bp, subelement calculation, minimum subelement size of 1 bp, and no graphical output. As no sequencing read confirmation was performed, each analysis was run on a single processor. Total processing time, i.e. “Elapsed (wall clock) time,” and maximum memory usage, i.e. “Maximum resident set size”, of each analysis was assessed using GNU time v1.7-24 (Linux) or system standard BSD time (OS X).

**Supplemental Results:**

**ClustAGE gene distribution analysis performance:**

In the ClustAGE analysis of the 14 strains of *P. aeruginosa* using a minimum nucleotide sequence identity cutoff of 85%, the bin reference sequences contained a total of 3,260 CDS where at least 50% of the gene, by length, was present within the AGE. To determine the relative accuracy of calling the presence or absence of a bin reference gene within the accessory sequence of the other genomes aligned by ClustAGE, presence of orthologous genes between the accessory genomes of all 14 *P. aeruginosa* strains in this analysis was inferred by reciprocal best Blast hits (RBB) [1,2] and compared to ClustAGE output. For the purposes of comparison, a gene present in a bin reference AGE was considered to be present within the accessory genome of one of the other 13 strains if at least 50% of the length of the gene was covered by aligned sequence from that strain’s accessory genome. In this fashion a total of 42,380 (3,260 x 13) comparisons were made to RBB results. When RBB results without filtering for minimum amino acid sequence identity were compared, the ClustAGE results matched the RBB results for presence or absence of a gene within a strain in 38,113 (89.9%) instances. See table in Additional file 5 for details of results presented below.

Among the comparisons in which ClustAGE and RBB predictions did not agree, there were 3,686 (8.7%) instances where an orthologous CDS pair was predicted by RBB, but less than 50% of the gene was covered by accessory element alignments from that strain in ClustAGE. In total, there were 3,686 (8.7%) instances where this was the case. One likely reason for this high number of missing alignments is that the basic definition of a positive RBB hit is based only on a minimum e-value score of 1 x 10^-6^ and at least 50% gene coverage by length for each pairwise comparison. The default settings of ClustAGE are more conservative, including only alignments with at least 85% nucleotide sequence similarity, therefore alignments of accessory elements containing the apparent RBB orthologs with lower sequence similarity would not be expected to be identified by ClustAGE. When a cutoff of at least 85% amino acid sequence identity in the RBB result was used, the number of discrepant results decreased to 443 (1.0%) and the number of concordant results increased to 41,176 (97.2%). Many genes that were predicted by RBB to have orthologs with high amino acid sequence identity, but were still not identified by ClustAGE alignments were smaller genes found within longer genomic regions aligned by BLAST that had an overall nucleotide sequence identity below the default ClustAGE threshold of 85%.

The converse type of discordant result in comparing ClustAGE alignment results to RBB predictions was instances of alignment of accessory sequence against a gene in ClustAGE, but no orthologous pair of genes between the strains predicted by RBB. The number of discrepancies of this type was 581 (1.4%) instances, which increased to 761 (1.8%) instances when a cutoff of 85% amino acid sequence identity was applied to RBB hits. A possible reason for these discrepancies was that differences in gene annotation methods between the strains would result in either over- or under-prediction of coding sequences in some strains. In these cases, ClustAGE alignment of nucleotide sequences would be accurate, but would not be reflected in RBB results, which depend on amino acid alignments of previously annotated coding sequences between strains. To examine this possibility, the amino acid sequences of the 349 CDS in the 761 instances of ClustAGE alignment without RBB support were used as query sequences in translated BLAST analyses (tblastn) [3,4] against the accessory genome nucleotide sequences of the 14 input strains. A gene was considered present if it covered at least 50% of the query gene with a minimum sequence identity of 85%. In 433 instances a query gene was found in the subject accessory genome sequence by tblastn alignment. This decreased the number of discrepant instances to 328 (0.8%) and increased the concordant results to 41,609 (98.2%) instances. Many of the remaining discrepancies likely arise from instances where ClustAGE alignments with nucleotide sequence identities of ≥ 85% will contain genes with RBB amino acid sequence identities of < 85% as few nucleotide differences can result in a greater proportion of amino acid encoding changes, depending on what position in the codons the base differences occur.

Varying the minimum alignment sequence identity threshold in ClustAGE also resulted in changes in the AGE annotation assignment results. Decreasing the nucleotide sequence alignment identity to 80% resulted in a smaller total number of reference genes in the ClustAGE output bins due to some AGEs that had been separate under an 85% sequence identity cutoff to be clustered together with the lower sequence identity cutoff. With the more permissive ClustAGE cutoff, there was also a greater proportion of gene assignments matching RBB results. This increase in concordance with RBB is due primarily to a decrease in the number of RBB ortholog predictions not confirmed by ClustAGE, but a concomitant increase in ClustAGE results not supported by RBB hits is also seen reflecting an increase in non-specific alignments. Conversely, the more restrictive 90% sequence identity threshold for ClustAGE resulted in fewer gene assignments concordant with RBB, again due primarily to an increase in the number of RBB ortholog predictions not confirmed by ClustAGE despite a decrease in ClustAGE alignments not confirmed by RBB.

**References:**

Rfdafasfja;sldfjear

1. Tatusov RL, Koonin EV, Lipman DJ: **A genomic perspective on protein families**. *Science* 1997, **278**(5338):631-637.

2. Bork P, Dandekar T, Diaz-Lazcoz Y, Eisenhaber F, Huynen M, Yuan Y: **Predicting function: from genes to genomes and back**. *J Mol Biol* 1998, **283**(4):707-725.

3. Altschul SF, Gish W, Miller W, Myers EW, Lipman DJ: **Basic local alignment search tool**. *J Mol Biol* 1990, **215**(3):403-410.

4. Altschul SF, Madden TL, Schaffer AA, Zhang J, Zhang Z, Miller W, Lipman DJ: **Gapped BLAST and PSI-BLAST: a new generation of protein database search programs**. *Nucleic Acids Res* 1997, **25**(17):3389-3402.

5. Camacho C, Coulouris G, Avagyan V, Ma N, Papadopoulos J, Bealer K, Madden TL: **BLAST+: architecture and applications**. *BMC Bioinformatics* 2009, **10**:421.

6. Ward N, Moreno-Hagelsieb G: **Quickly finding orthologs as reciprocal best hits with BLAT, LAST, and UBLAST: how much do we miss?** *PLoS One* 2014, **9**(7):e101850.
