# Supplementary material for: ClustAGE: a tool for clustering and distribution analysis of bacterial accessory genomic elements
Source: BMC Bioinformatics. 2018 Apr 20;19:150. doi: 10.1186/s12859-018-2154-x (PMC5910555; doi:10.1186/s12859-018-2154-x)
Supplement: Supplementary file 7 — ClustAGE computational performance. Randomly selected sets of accessory genomic elements from identified from Pseudomonas aeruginosa whole genome sequences were analyzed by ClustAGE. Analyses were performed on a server platform running Ubuntu (Linux, blue) and on a desktop computer running OS X (Mac, orange). Time to completion of ClustAGE analysis (solid lines) and maximum memory usage (dashed lines) were measured for each analysis. Each point represents the average of 5 replicate analyses at each number of input genomes. Error bars represent the standard error of the mean. (PDF 72 kb) [file 12859_2018_2154_MOESM7_ESM.pdf]

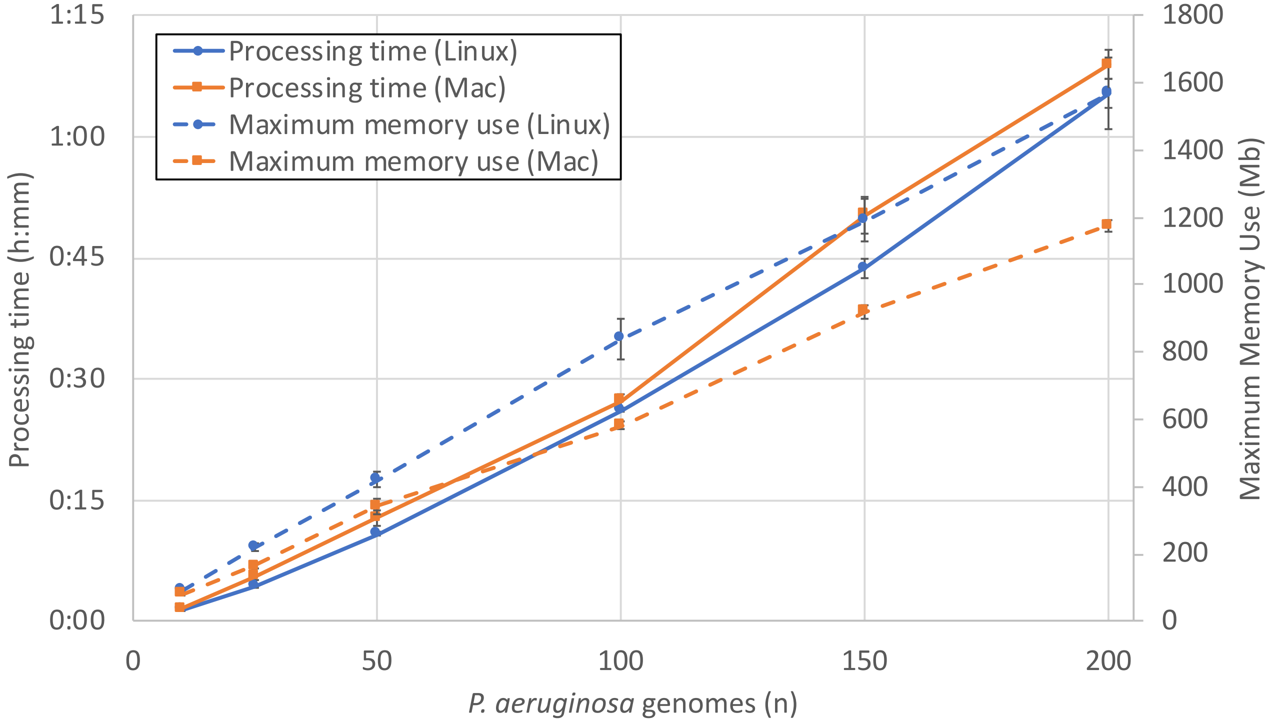

Supplemental Figure 1: ClustAGE computational performance. Randomly selected sets of accessory genomic elements from identified from *Pseudomonas aeruginosa* whole genome sequences were analyzed by ClustAGE. Analyses were performed on a server platform running Ubuntu (Linux, blue) and on a desktop computer running OS X (Mac, orange). Time to completion of ClustAGE analysis (solid lines) and maximum memory usage (dashed lines) were measured for each analysis. Each point represents the average of 5 replicate analyses at each number of input genomes. Error bars represent the standard error of the mean.
